# Antibiotic use and hygiene interact to influence the distribution of antimicrobial-resistant bacteria in low-income communities in Guatemala

**DOI:** 10.1038/s41598-020-70741-4

**Published:** 2020-08-13

**Authors:** Brooke M. Ramay, Mark A. Caudell, Celia Cordón-Rosales, L. Diego Archila, Guy H. Palmer, Claudia Jarquin, Purificación Moreno, John P. McCracken, Leah Rosenkrantz, Ofer Amram, Sylvia Omulo, Douglas R. Call

**Affiliations:** 1 Center for Health Studies, Universidad del Valle de Guatemala, 181 Avenida 11-95, Guatemala City, 01015 Guatemala; 2 Paul G. Allen School for Global Animal Health, Washington State University, Pullman, WA 99164 USA; 3 Food and Agriculture Organization of the United Nations, United Nations Complex, UN Avenue, Gigiri, PO Box: 30470, Nairobi, 00100 Kenya; 4 Nelson Mandela African Institution of Science and Technology, P.O.BOX 447, Arusha, Tanzania; 5 Department of Geography, Simon Fraser University, Burnaby, BC V5A 1S6 Canada; 6 Elson S. Floyd College of Medicine, Washington State University, PO Box 1495, Spokane, WA USA

**Keywords:** Diseases, Public health

## Abstract

To examine the effects of poor sanitation and hygiene on the prevalence of antimicrobial-resistant bacteria, we surveyed households in two rural and two urban communities in Guatemala (N = 196 randomly selected households). One adult (≥ 18-years old) and, when available, one child (≤ 5 years-old) provided a stool sample. Up to 48 presumptive *Escherichia coli* isolates were collected from each stool sample (n = 21,256 total) and were subjected to breakpoint assays for ten antibiotics. Mixed-effects logistic models were used to identify potential factors influencing the likelihood of harboring antibiotic-resistant bacteria. For nine out of ten antibiotics, the odds of detecting resistant bacteria decreased by ~ 32% (odds ratios, OR 0.53–0.8, P < 0.001) for every unit of improvement of a hygiene scale. Hygiene differences between households had a greater impact on prevalence compared to antibiotic use differences. The likelihood of detecting resistant isolates was lower for five antibiotics among households that boiled raw milk before consumption (OR 0.31–0.69), and higher for nine antibiotics in urban households (OR > 1.89–9.6). Poor hygiene conditions likely obscure effects of individual antibiotic use, presumably due to enhanced microbial transmission. Consequently, efforts to improve antibiotic stewardship should be coupled with improving hygiene conditions.

## Introduction

Antimicrobial-resistant infections contribute to hundreds-of-thousands of deaths worldwide^1^. To address this threat, public and private organizations are engaged in programs to improve surveillance for antimicrobial-resistant organisms, to improve antibiotic stewardship, and to identify novel therapeutics, all of which are considered priority actions for combating antimicrobial resistance (AMR)^2–4^. The strategies adopted to limit AMR will vary globally, particularly across countries with different degrees of wealth and development. The most effective intervention will likely depend on prevailing conditions. For example, high-income countries and low-income countries can differ in antimicrobial resistance patterns, antimicrobial use practices, access to healthcare services (human and animal), sanitation and regulation infrastructures^5–9^.

Studies conducted within high-income countries, particularly those concerning healthcare facilities^10–12^ and the agricultural sector^13–15^, have associated reductions in antimicrobial use and improved stewardship with decreases in the prevalence of antimicrobial resistance. In contrast, studies examining global patterns of AMR find that antibiotic use is not positively correlated with resistance in all cases and may instead be correlated with indicators of transmission, including access to clean water^5^ and open defecation^6^. This lack of association between antibiotic use and AMR in some settings may result from conditions in less-wealthy countries where the prevalence of antimicrobial resistance is more closely correlated with general transmission of bacteria^16^. Collignon et al. recently demonstrated that at a country-scale, several factors including poor infrastructure likely contribute to the overall prevalence of antibiotic-resistant pathogenic *Escherichia coli* in a country^5^. At a local scale, it has been postulated that if the frequency of transmission is high, the specific effect of antibiotic use on the prevalence of resistant bacteria can be overshadowed^16,17^, possibly due to transmission decoupling antibiotic selection events from the distribution of antimicrobial-resistant bacteria. If correct, we should observe a stronger correlation between antibiotic use practices and the prevalence of antibiotic resistant bacteria when transmission is limited.

To test how antibiotic use and transmission factors interact to influence the distribution of antimicrobial-resistant bacteria in communities where these factors vary, we studied the distribution of antibiotic-resistant *Escherichia coli* in four communities from the western highlands and lowlands of Guatemala. Study communities were identified to represent presumed variance in transmission rates between urban and rural areas determined by differences in sanitation, hygiene and population density. Highland and lowland communities were selected to maximize variation in the presumed prevalence of illnesses that drive antibiotic consumption and associated healthcare practices when experiencing vector-borne febrile illness, diarrhea, and respiratory illness. If there is a significant interaction between antibiotic use and transmission factors, this has important implications for how public health policies should direct resources to limit the prevalence of antibiotic-resistant bacteria in these communities.

## Methods

### Study design, population, and sample size

**Figure 1:**
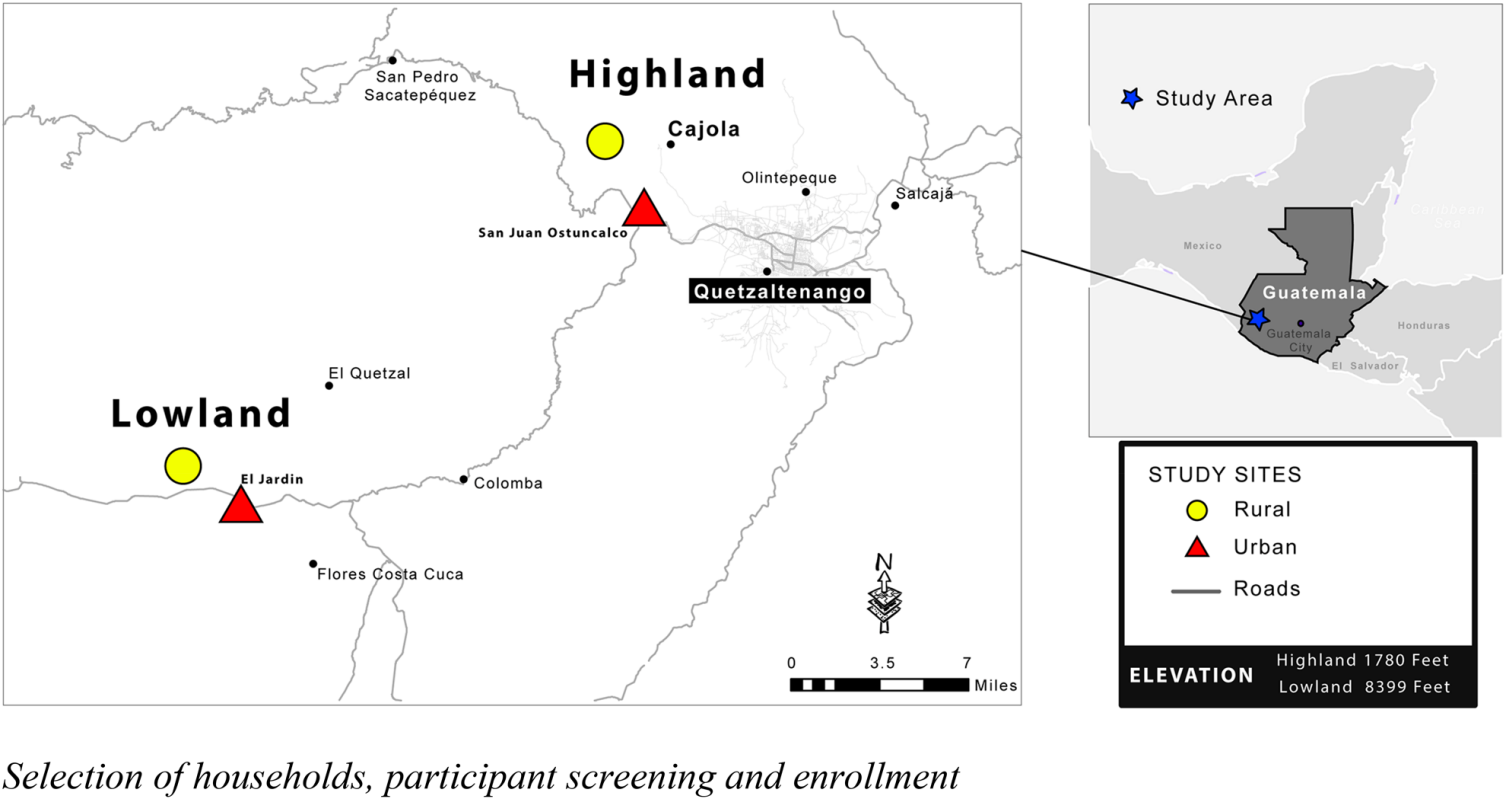
Map of selected communities. Base layers for map were downloaded from © OpenStreetMap contributors https://www.vdsgeo.com/osm-data.aspx and licensed under Creative Commons Attribution-ShareAlike 2.0. The map was created using ESRI ArcGIS. "Release 10." Redlands, CA: Environmental Systems Research Institute.

We executed a repeated-measures study that included a cross-sectional questionnaire and stool sample collection in October/November of 2017 (phase 1) and March/April of 2018 (phase 2). Within the department of Quetzaltenango (pop. ≈155,000), two municipalities were selected by convenience (from a total of 24) to represent differences in climate, altitude, and ethnic cultural practices; all presumed to result in differences in the transmission and incidence of infection and resulting antibiotic use (Fig. 1). The municipality of San Juan Ostuncaclo is situated in the highlands and is primarily inhabited by the “Maya-Mam” indigenous ethnic group. The urban city center of San Juan Ostuncaclo was paired with the rural community of Monrovia predominantly inhabited by Maya-Mam small-holder farmers who speak “Mam”^18^. We selected two communities in the lowland municipality of Coatepeque (average elevation 149 m): the urban community of El Jardin and the peri-urban (“rural”) community of La Unión. In both lowland communities, predominant inhabitants include the Spanish-speaking Mestizo. Both the lowland and highland municipalities have high rates of poverty, although markedly higher in the highlands community (73% in San Juan Ostuncalco, and 43% in Coatepeque^19^).

### Selection of households, participant screening and enrollment

We consulted several data sets to generate lists for random selection of households. In 2016, the Ministry of Health and Social Assistance (MSPAS) in collaboration with the Universidad del Valle de Guatemala, carried out a census of households in both lowland communities. 2017 MSPAS census information was available for Monrovia. Census information was not available for San Juan Ostuncalco. Instead, we used Google Earth satellite imagery^20^ to identify city-blocks within the urban center that included rooftops. We then randomly selected 25% of these blocks. Each door/gate for each block was approached to document the number of households behind each entrance^21^. Households were subsequently numbered and random selection was used to identify households for potential enrollment. Following selection, household representatives, participants and child guardians were taken through informed consent in Spanish or Mam by bilingual field technicians. Informed consent was obtained from participants and from parents and child guardians of child participants. One adult and one child five years old or younger (if available) were randomly selected (“participants”) from the list of household residents. The study protocol was approved by Washington State University in Pullman Institutional Review Board (15895-001), the Universidad del Valle de Guatemala-Center for Health Studies Ethics committee (159-01-2017), and the Guatemalan Ministry of Health Ethics Committee (10-2017). All research was performed in accordance with the relevant guidelines and regulations of these institutions.

Survey instruments were based on similar studies^17,22^ and included components from water sanitation and health (“WASH”) defining poor sanitation as meeting one of the following conditions set out by the Joint Monitoring Programme for Water Supply, Sanitation and Hygiene (JMP) of WHO and UNICEF: unimproved JMP toilet (pit latrine without slab/open/blind well), shared toilet, toilet not cleaned within the last four weeks, or the last time a child passed stool it was not disposed of in toilet^23^. See Supplement pgs. 2–4 for more information on selection criteria and questionnaire development.

Stool collection kits and explicit instructions for stool collection were given to study participants enrolled in phase 1, the same participants were approached for a second stool sample collection in phase 2. Participants were instructed to pass stool into any clean and dry container, directly into the study-kit stool collection container, or onto the Kraft paper provided in the study kit (see Supplemental “Instructions for Stool Collection”). Households were contacted by study personnel within 24 h of initial study contact to collect stool samples. These were then transported in cooler boxes with ice packs and stored under refrigeration for a maximum of 5 days until they were shipped to a reference laboratory at the regional hospital in Quetzaltenango where isolates of presumptive *E. coli* were obtained. Isolates were then tested for their susceptibility to ten antibiotics by using breakpoint assays^16,17,24^. For these assays, bacteria are grown on agar plates with fixed concentrations of antibiotic and they are considered “resistant” when growth occurs, or susceptible when growth does not occur. See Supplement pgs. 3–5 for information on isolation of *E. coli*, breakpoint assays, and validation of assays relative to classic agar diffusion assays.

### Statistical analysis

Selected independent variables were based upon results from reviews of antimicrobial resistance in low- and middle-income countries (e.g., the WHO Global Action Plan^25^) and from our own ethnographic work (i.e., recurrent practices we observed that likely impacted transmission/selection). Included variables represent the general categories of antibiotic use and access, hygiene and sanitation, sickness, and demographics (Table 1). See Supplement pgs. 5–6 for additional information on variable selection.

**Table 1:** Description of variables entered into multivariate models.

Variable name	Questionnaire/study design definition	Categories
**Independent measures**		
**AMR risk models**		
Adult_child	Participant is a child or an adult	0-Child 1-Adult
Rural_urban	Sample is from rural or urban community	0-Rural 1-Urban
Diarrhea	A binary variable indicating whether the household reported diarrhea in the past 14 days (phase 1) and/or diarrhea between study periods (phase 2)	0 = No 1 = Yes
Boiled milk	Whether the household boiled milk before consuming	0 = No 1 = Yes

	Scaled variable	Scale range
Antibiotic use scale	Created by summing three question categories 1) ever used antibiotics; 2) antibiotics used in last 14 days (phase 1); 3) antibiotics used between study phases. A smaller number of individuals answered yes to all study questions, so the scale was collapsed into three categories (0, 1, 2) with Level (0) = No to all three questions Level (1) = Yes to one of the three questions Level (2) = Yes to two or more of the questions	Scale 0–2
Household hygiene scale	A linear scale indicating increasing levels of household sanitation including whether feces were present on floor (reverse-coded), whether the floor was dirt (reverse-coded), whether the household had a clothes washer, an improved toilet, a private toilet, whether river water was used in household (reverse coded), ownership of animals (reverse coded), whether protozoa were detected in fecal samples (reverse-coded) and whether trash was disposed in an appropriate location	Scale 1–9

Model specification proceeded by entering independent variables into a mixed-effects logistic regression model that was clustered at the individual level. We specified models for all resistance phenotypes and a multidrug resistance phenotype (MDR), which we defined as resistance to three or more antibiotic classes. Results were summarized as odds ratios (OR), which we converted to percentage change in odds to facilitate interpretation (e.g., an OR of 1.45 equals a 45% increase in the odds of exhibiting prevalence). When specifying interactions between antibiotic use and hygiene/sanitation, continuous variables entered into interactions were first mean-centered to limit multicollinearity^26^. To minimize attention to potentially spurious correlations, we restricted our inferences to variables that were significantly associated (*P* < 0.05) with resistance to ≥ 3 antimicrobial resistance phenotypes. Model fit was good to adequate and tests of model fit are provided in Supplement pg 6 and Table S2.

## Results

### Descriptive results

A total of 196 households across all four communities (Table 2) participated in the study. Stool samples were obtained from 195 adults and 78 children accounting for 273 individuals from 196 households provided 480 stool samples collected during the study period. The average age of adult participants was 41 years (± 17 sd), and the average age of children was 2 years (± 1) (Table 3).

**Table 2:** Number of presumptive *E. coli* isolates across locations and by adult (≥ 18-years old) and children (≤ 5-years old).

	Number of households	Adults	Adult isolates	Children	Children isolates	Total isolates
San Juan Ostuncalco, highland, urban	49	49	3,975	22	1,645	5,620
Monrovia, highland, rural	50	50	4,362	32	2,600	6,962
El Jardin, lowland, urban	48	48	3,952	7	472	4,424
La Unión, lowland, rural	49	48	3,076	17	1,174	4,250
Total	196	195	15,365	78	5,891	21,256

**Table 3:** Characteristics of the study population, n = number of individuals; ±  = standard deviation.

	Highlands	Lowlands	Total
	n = 153	n = 120	n = 273
**Demographics**			
Proportion of adult participants in study population	65%	80%	71%
Average age of adults	40 (± 16)	42 (± 17)	41 (± 16)
Average age of children	2 (± 1)	2 (± 1)	2 (± 1)
Females	74%	78%	74%
Indigenous ethnicity	72%	9%	44%
Adult participant: no formal education	31%	20%	27%
Adult participant: literate (reads)	47%	68%	57%
**Household characteristics**			
JMP unimproved toilet^a^	11%	23%	16%
Shared toilet, not on premise	53%	57%	55%
> 3 people per sleeping room^b^	33% (48/146)	43% (31/91)	37% (87/237)
Dirt floors	30%	32%	30%
Household consumes any type of milk	45%	57%	50%
Household consumes raw milk only	37%	28%	33%
Raw milk boiled before consumption	37%	22%	30%
Milk stored^c^ in household: raw milk, boiled	11% (6/56)	35% (9/26)	18% (15/82)
Milk stored^c^ in household: packaged milk	38% (5/13)	79% (24/34)	61% (29/47)
**Antibiotic use by participant**			
Ever used antibiotics	41%	86%	61%
Used antibiotics in the past 14 days	0% (0/70)	11% (8/75)	5% (8/145)
Used antibiotics between study phases^b^	11% (9/84)	30% (14/46)	18% (23/130)

^a^Pit latrine without a slab or platform, hanging latrines or bucket latrines.

^b^Underreporting, total number of responses indicated in parenthesis.

^c^“Stored” means that the milk was not consumed immediately upon purchase.

The majority of enrolled households in these communities were characterized as having poor sanitation (89%, n = 237/265), including 16% of households having an JMP unimproved toilet and about half of households sharing a toilet that was not located on the premises (55%). Unimproved toilets were more common in rural areas (29%) versus urban areas (2%). More toilets were reported as shared and located outside the household in rural areas 73% compared with 33% in urban households. Thirty-seven percent of households were defined as “overcrowded” (> 3 people per sleeping room). Lowland regions had higher overall report of antibiotic use (see Table 3), and higher reported prevalence of diarrhea in phase 1 versus highland communities (17% versus 1%).

### Finding 1: The relative distribution of antimicrobial-resistant bacteria differed between locations, ages, and sexes

**Figure 2:**
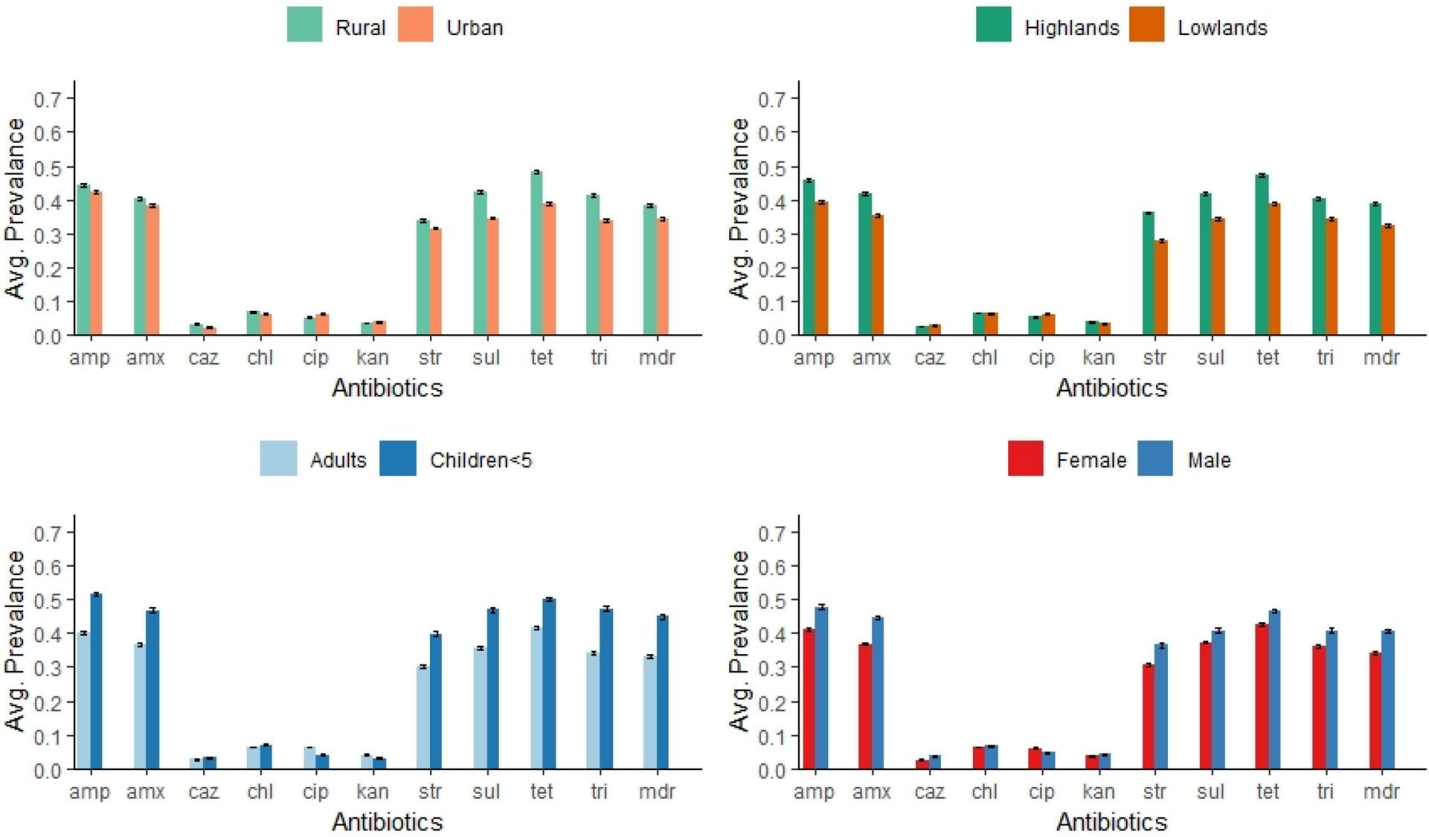
Mean (+ standard error) of antimicrobial resistant bacteria isolated from fecal samples collected from different locations, ages, and gender. Coatepeque and San Juan Ostuncalco, 2017–2018. Antibiotics included amp (ampicillin), amx (amoxicillin), caz (ceftazidime), chl (chloramphenicol), cip, (ciprofloxacin) and kan (kanamycin), str, (streptomycin), sul (sulfamethoxazole), tet (tetracycline), tri (trimethoprim). MDR indicates resistance to three or more classes of antibiotics. Standard errors account for within subject correlation.

A total of 21,256 g-negative, lactose-fermenting bacteria (presumptive *E. coli*) were isolated from 273 individuals sampled. The prevalence of resistance to ampicillin, amoxicillin, streptomycin, sulfamethoxazole, trimethoprim, tetracycline, and the MDR resistance phenotype was about four times higher (> 30%) compared to ceftazidime, chloramphenicol, ciprofloxacin and kanamycin (< 8%). Breakpoint and disc diffusion assay results indicated the two methods provided a consistent estimate for the proportion of resistant strains across ten different antibiotics (n = 99 isolates; *r* = 0.99; see Supplement Fig. S1). In general, the mean prevalence of antimicrobial-resistant bacteria was higher in rural compared to urban areas, the highlands compared to the lowlands, in children relative to adults, and for men relative to women (Fig. 2).

### Finding 2: Antimicrobial resistance was associated with increasing frequency of antibiotic use, household hygiene levels, milk consumption, and diarrhea

Antibiotic use was associated with a higher likelihood of harboring detectable antimicrobial-resistant bacteria, but almost exclusively in households classified as Level 2 in the antibiotic use scale (Table 1) which reported consuming the most antimicrobials frequently. This group comprised 11% of the sampled population (see Table 1 for variable definition). For these individuals there was a ≈50–113% increase in the likelihood of detecting bacteria resistant to all antibiotics with the exception of kanamycin, which was associated with an 83% decrease (Table 4). In contrast, lower levels of antibiotic use (≈50% of the sampled population) were associated with ≈50–70% lower odds of detecting bacteria resistant to chloramphenicol, ciprofloxacin, and kanamycin but were associated with 54% increased odds of detecting bacteria resistant to sulfamethoxazol.

**Table 4:** Multivariate analysis of the correlates of antibiotic resistance.

Antibiotics	Level 1^a,b^ AB use	Level 2 AB use	Household hygiene scale	Participant had diarrhea	Household boiled milk	Adult/child Adult = 1	Rural/urban Urban = 1
Ampicillin	0.89 (0.68–1.18)	1.53*** (1.21–1.95)	0.69*** (0.63–0.77)	1.04 (0.86–1.25)	0.82 (0.52–1.29)	0.42*** (0.26–0.67)	2.10*** (1.31–3.37)
Amoxicillin	1.05 (0.80–1.38)	1.72*** (1.35–2.18)	0.77*** (0.70–0.84)	0.96 (0.80–1.15)	0.69* (0.45–1.06)	0.46*** (0.30–0.72)	1.56* (1.00–2.44)
Ceftazidime	0.91 (0.48–1.73)	0.65* (0.39–1.07)	0.58*** (0.46–0.73)	3.34*** (2.12–5.26)	0.38** (0.16–0.89)	0.77 (0.35–1.70)	2.78** (1.14–6.77)
Chloramphenicol	0.23*** (0.13–0.39)	1.82*** (1.17–2.82)	0.69*** (0.59–0.80)	0.27*** (0.18–0.40)	0.40*** (0.21–0.75)	1.21 (0.64–2.29)	3.31*** (1.67–6.55)
Ciprofloxacin	0.54** (0.31–0.93)	2.10*** (1.34–3.32)	0.51*** (0.41–0.63)	1.28 (0.90–1.82)	0.31*** (0.14–0.70)	2.25** (1.01–5.03)	5.96*** (2.50–14.22)
Kanamycin	0.28*** (0.15–0.51)	0.17*** (0.09–0.31)	1.31*** (1.10–1.55)	5.75*** (3.69–8.96)	0.85 (0.44–1.65)	1.24 (0.63–2.43)	1.25 (0.61–2.59)
Streptomycin	1.28* (0.96–1.72)	1.81*** (1.41–2.31)	0.80*** (0.72–0.88)	0.70*** (0.57–0.85)	0.73 (0.45–1.18)	0.40*** (0.24–0.65)	1.35 (0.83–2.22)
Sulfamethoxazole	1.54*** (1.15–2.07)	1.53*** (1.21–1.95)	0.56*** (0.50–0.62)	1.49*** (1.23–1.80)	0.93 (0.55–1.59)	0.45*** (0.26–0.78)	1.70* (0.99–2.93)
Tetracycline	0.96 (0.73–1.27)	1.59*** (1.26–2.01)	0.75*** (0.68–0.82)	0.92 (0.76–1.12)	0.62** (0.39–0.97)	0.52*** (0.33–0.83)	1.11 (0.69–1.77)
Trimethroprim	0.95 (0.72–1.27)	1.23 (0.96–1.57)	0.69*** (0.62–0.76)	1.14 (0.95–1.38)	0.93 (0.59–1.47)	0.43*** (0.27–0.69)	1.44 (0.90–2.31)
MDR	0.95 (0.71–1.27)	1.67*** (1.30–2.13)	0.62*** (0.56–0.69)	1.05 (0.87–1.27)	0.66 (0.40–1.11)	0.36*** (0.21–0.62)	2.21*** (1.31–3.74)

Across all antibiotics, the number of observations is 21,256 and the number of groups (individuals) is 273. Coefficients are provided with 95% confidence intervals. See Table 1 for variable definitions.

^a^***P < 0.01, **P < 0.05, *P < 0.1

^b^See Table 1 for definitions.

^c^Constant indicates the predicted mean odds ratio (OR) when all variables are 0.

For household hygiene, every unit increase in the hygiene scale (i.e., better hygiene) was associated with a ≈30–50% decrease in odds of detecting resistance to all antibiotics (Table 4). The sole exception to this trend was kanamycin, where increasing household hygiene was associated with a 31% increase in odds of detecting kanamycin resistant bacteria. Individuals who reported a diarrheal episode had substantially higher odds of having bacteria resistant to ceftazidime (234%), kanamycin (475%) and sulfamethoxazole (49%), but significantly reduced odds for detecting bacteria resistant to chloramphenicol (73%) and streptomycin (70%). Households that reported drinking cow or goat milk and boiling it before consumption had lower odds of resistance than households who (1) drank raw milk (2) drank powdered milk (3) drank boxed (packaged) milk (4) did not drink milk at all. These households were associated with a ≈ 40–70% decrease in odds of having bacteria resistant to ceftazidime, chloramphenicol, ciprofloxacin, or tetracycline.

Adults had ≈ 60% reduced odds of harboring bacteria exhibiting resistance to most antibiotics compared to children. This significant difference did not hold for the less frequently encountered resistance phenotypes including ceftazidime, chloramphenicol and kanamycin. Adults had significantly higher odds of (125%) of exhibiting resistance to ciprofloxacin. For participants residing in urban areas, there was a significantly increased odds of detecting bacteria resistant to ampicillin (110%), ceftazidime (178%), chloramphenicol (231%), ciprofloxacin (496%) and the MDR phenotype (121%) (Table 4).

### Finding 3: Hygiene levels are strongly associated with prevalence of antimicrobial-resistant bacteria, but antibiotic use is also associated when sanitation is good

**Figure 3:**
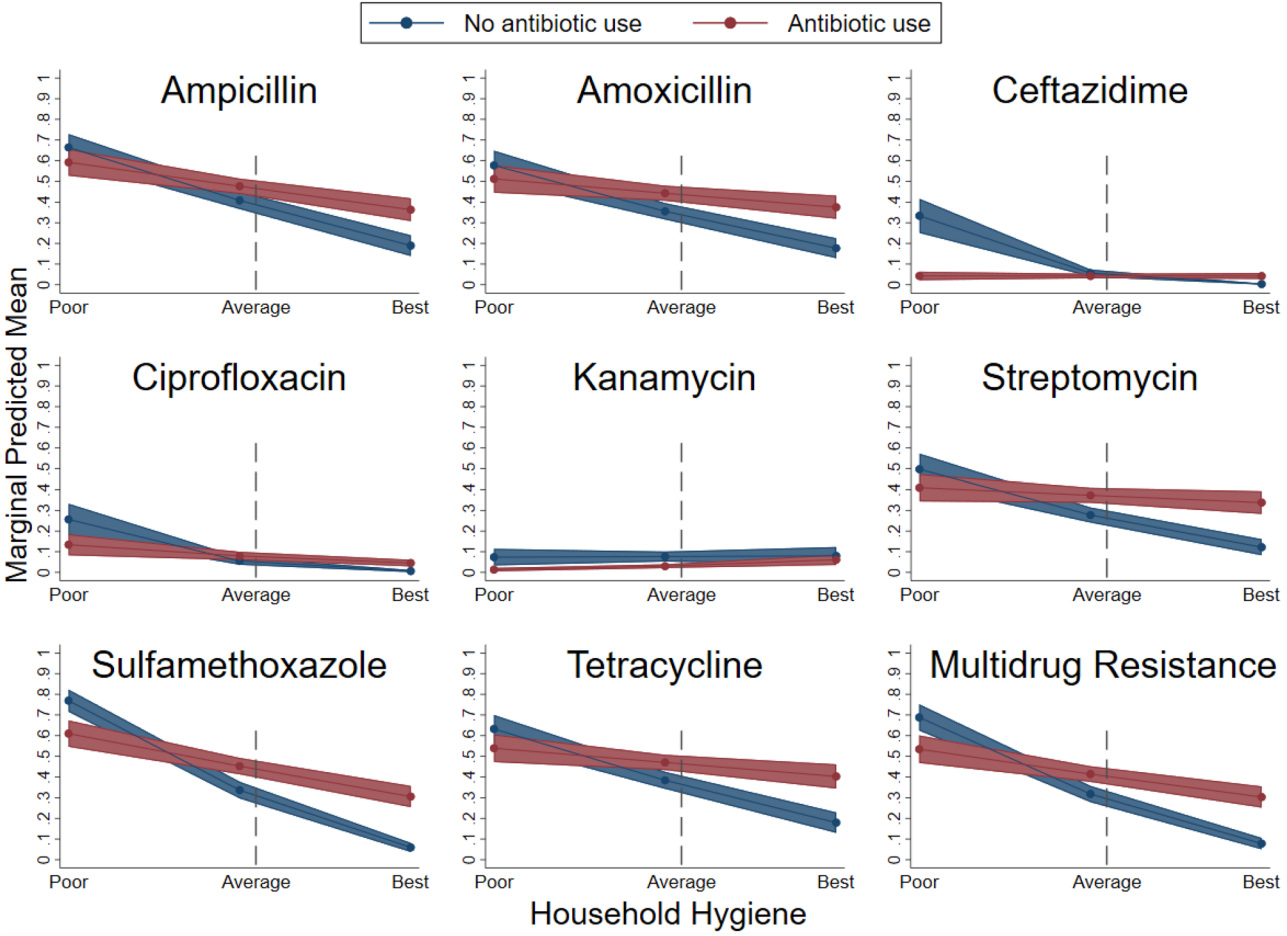
Impact of antibiotic use on resistance across household hygiene levels. The dashed vertical line indicates the average household hygiene score. Chloramphenicol and trimethoprim are not shown because the interaction was not significant.

To assess how prevalence of antibiotic-resistant bacteria is affected by antibiotic use across household hygiene levels, interaction terms were entered into the resistance models (see Supplement Table S2). Significant interactions (*P* < 0.05) between antibiotic use and hygiene/sanitation were found for all antibiotics except for chloramphenicol and trimethoprim. To facilitate interpretation, we plotted the relationship between hygiene and antibiotic resistance separately for individuals that reported using antibiotics (n = 104 individuals) and for those who reported not using antibiotics (n = 173 individuals) (Fig. 3). At average household hygiene levels (≈ 6), there was little difference in predicted resistance levels between users and non-users of antibiotics. For households with the best hygiene, those reporting antibiotic use had a higher predicted probability of resistant bacteria compared to non-users where the opposite occurred for the worst hygiene conditions. Confidence in the separation between antibiotic users and non-users was greater for high hygiene levels compared with low hygiene levels, as indicated by consistently non-overlapping 95% confidence intervals. For non-users, the differences between marginal predictions at the lowest and highest levels of hygiene can be interpreted as the effect of hygiene while holding variables (other than antibiotic use) constant at their means. This effect, across the majority of antibiotics, results in a 50% decrease in predicted probabilities from the lowest to highest levels of hygiene. For example, isolates from non-users in households with the lowest levels of hygiene have a predicted probability of 70% for exhibiting resistance to ampicillin while this probability decreases to about 25% in non-user households with the best hygiene levels. In contrast, for households that reported using antibiotics, the decrease in probabilities across low and high hygiene levels was approximately 25%, indicating that the effects of hygiene on the probability of detecting antimicrobial-resistant bacteria are moderated by antibiotic use.

## Discussion

We found that each unit of improvement in the household hygiene index was associated with a ≈30–50% reduction in the likelihood of detecting antimicrobial-resistant bacteria within people. Importantly, as hygiene improved, the effects of using antibiotics became increasingly apparent. That is, poor hygiene modifies the effects of antibiotic use, but this modification is not a strong issue when hygiene conditions are very good. This is consistent with studies in diverse contexts indicating that hygiene may play an important role in the distribution and persistence of antibiotic resistant bacteria within communities in low- and middle-income countries^5,6,16,17,27^. An immediate implication of this interaction is that efforts to improve antibiotic stewardship, including control of unregulated access to antibiotics, may have little immediate impact on the overall prevalence of antimicrobial resistant bacteria when poor hygiene conditions are prevalent.

Given the considerable increase in odds in detecting antimicrobial-resistant bacteria across our household hygiene scale, we surmise that bacterial transmission is the primary mechanism influencing the prevalence of antimicrobial-resistant bacteria. We also found that individuals who reported boiling their raw milk before consumption exhibited a significantly lower prevalence of bacteria that were resistant to amoxicillin, ceftazidime, chloramphenicol, ciprofloxacin and tetracycline compared to those who consumed raw milk, drank powdered or packaged milk, and those that reported not consuming any milk. These results, specifically in relation to households who consumed raw milk, are consistent with an earlier report showing that Maasai pastoralists in Tanzania who boiled their milk exhibited reduced prevalence of antimicrobial-resistant bacteria^17^. Hygiene practices may also account for the higher predicted resistances for those who used powdered or packaged milk. About one-third of households reported not treating their water and mixing this with powdered milk would increase the risk of transmission and sickness. Packaged milk was reported to be stored for longer periods compared to fresh milk, which could also increase the risk of transmission and sickness if sub-standard storage practices (e.g., lack of refrigeration) are used. The higher prevalence of resistance in households reporting no milk consumption is more challenging to explain. It may be that households reporting no milk consumption are consuming other higher risk alternatives which could also be related to the ability to purchase milk. To examine this further, we generated a correlation matrix and found a weak positive correlation between milk consumption and wealth (*r* = 0.13) and between milk consumption and household hygiene (*r* = 0.13) (see supplement Table S3). Further study is likely needed to understand the constraints and substitutions that impact milk consumption in these communities. While the relationship between milk consumption and resistance is clearly complex, the different environments and cultures for which milk handling practices appear important (e.g., Guatemalan and Tanzanian communities), suggests that milk hygiene practices may play an important role in the transmission and persistence of antimicrobial resistance within low-income communities. However, further study is needed to determine the extent of improvement that might be achieved through mitigation of milk hygiene practices.

As with other studies conducted in low- and middle-income countries, we document a positive association between living in more urban areas and antimicrobial resistance. And as with other low- and middle-income countries, poor access to clean water, poor hygiene and sanitation conditions are evident in Guatemala although the extent of these issues differ based on urban and rural settings^28,29^. For the present study, compared to rural households, individuals living in urban areas had a ~ 170% increase in the odds of harboring bacteria resistant to ampicillin, amoxicillin, ceftazidime, chloramphenicol, ciprofloxacin and the MDR phenotype. It is worth emphasizing that the relationship between urban living and the odds of detecting antimicrobial-resistant bacteria only emerges after controlling for antibiotic use, hygiene and sanitation, and age differences. Without controlling for these differences, the prevalence of resistant bacteria is seemingly higher for most antibiotics in rural areas than in urban areas (see Fig. 2). After controlling for hygiene and antimicrobial use, this relationship becomes inverted, suggesting that there are likely other factors contributing to a higher prevalence of antimicrobial resistance in urban areas.

Antibiotic use had little measurable impact in sub-optimal hygiene conditions. The overall prevalence of antibiotic-resistance phenotypes is consistent with the relative availability and cost of antibiotics sold without a prescription in these communities (e.g., sold at shops called *tiendas*). Medications in Guatemala are subsidized by the government through the Ministry of Health system and the National Social Security health care system, yet frequent stock-outs force most Guatemalans to purchase medications out-of-pocket at private pharmacies^29^. In these establishments, medications can be up to 20 times the international listed price [e.g., costing up to 15 days wages for third-generation cephalosporins^30^]. In contrast, amoxicillin and tetracycline are considerably more affordable and widely available in *tiendas*, perhaps reflecting antibiotic use in these communities where the average prevalence of resistance to amoxicillin and tetracycline was approximately 40%.

We also detected several cases where factors including antibiotic use, hygiene, and diarrheal episodes were correlated with both an increase and a decrease in the odds of harboring detectable levels of antimicrobial-resistant bacteria. For example, while antibiotic use was mostly correlated with higher odds of detecting resistance bacteria, it was correlated with lower odds of detecting bacteria resistant to Kanamycin (see Table 4). There are likely two mechanisms underlying these observations. First, some resistance traits may have increased in prevalence despite the absence of commensurate use of corresponding antibiotics (e.g., diarrheal episodes and resistance to chloramphenicol). These changes always occurred in the context of similar changes in other antibiotic-resistance phenotypes, and one likely explanation is co-selection that occurs when the genes encoding these resistance traits are genetically linked. In essence, selection for one resistance phenotype co-selects for any linked traits. In some cases, it is also possible that antibiotic use can “filter” a population, by favoring strains that have the associated resistance gene and this would simultaneously increase the prevalence of any other genetic resistance genes found with these strains (i.e., “co-selection”). At the same time there would be a decrease in the prevalence of strains that do not harbor a resistance gene for the antibiotic being used. A potential example of this is the relationship between the likelihood of detecting antimicrobial resistance with recent episodes of diarrhea, where prevalence of resistance to ceftazidime, kanamycin and sulfamethoxazole increased, but prevalence of resistance to chloramphenicol and streptomycin decreased.

As with any epidemiological study, we are limited to identifying correlated variables for largely uncontrolled systems, making clear cause-and-effect relationships difficult to identify due to confounded variables. An example of this is the apparent higher prevalence of resistance in rural households shown in the univariate comparison (Fig. 2) vs. the statistically higher likelihood of detecting resistant bacteria in urban households once several other variables are controlled. Furthermore, our analysis of antibiotic use may be compromised by limitations of participant recall^31^, and the commensurate limitations on the ability to gather accurate data about the magnitude and frequency of antibiotic use. In addition, while we modeled household hygiene and sanitation as a linear variable, hygiene and sanitation are clearly complex phenomena that include many interacting factors so that an increase in one factor (handwashing frequency) likely does not reflect the same impact on the prevalence of antimicrobial resistance as another (e.g., improved toilet). Nevertheless, we argue our composite measure represents a measure of of household hygiene and sanitation with changes in the scale reflective of general increases and decreases in household hygiene and sanitation.

The robust relationship between hygiene and resistance in the sampled Guatemalan communities, along with the interaction between hygiene and antibiotic use, provides important implications for the efficacy of stewardship efforts globally when aggregate hygiene levels are compromised. In such cases, investment in infrastructure to improve hygiene can be easily justified as a tool to limit the proliferation of antimicrobial resistance in communities across the globe. As this study highlights, assigning priorities and subsequent development of targeted strategies will require analysis of a greater spectrum of living conditions, using cross-cultural investigations developed and implemented by interdisciplinary teams from the natural and social sciences.

### Ethics committee approval

The study protocol was approved by Washington State University in Pullman Institutional Review Board (15895-001), the Universidad del Valle de Guatemala-Center for Health Studies Ethics committee (159-01-2017), and the Guatemalan Ministry of Health Ethics Committee (10-2017).

## Supplementary information

Supplementary file 1.

Supplementary file 2.

Supplementary file 3.

Supplementary file 4.

Supplementary file 5.
