# Supplementary material for: Antibiotic use and hygiene interact to influence the distribution of antimicrobial-resistant bacteria in low-income communities in Guatemala
Source: Sci Rep. 2020 Aug 13;10:13767. doi: 10.1038/s41598-020-70741-4 (PMC7426860; doi:10.1038/s41598-020-70741-4)
Supplement: Supplementary file 1 [file 41598_2020_70741_MOESM1_ESM.pdf]

Antibiotic use and hygiene interact to influence the distribution of antimicrobial-resistant bacteria in low-income communities in Guatemala

Brooke M. Ramay[¥1,2], Mark, A. Caudell[¥2,3,], Celia Cordon-Rosales*[1], L. Diego Archila[1], Guy H. Palmer[2,4], Claudia Jarquin[1], Purificación Moreno[1], John P. McCracken[1], Leah Rozenkrantz[5], Ofer Amram[2,6], Sylvia Omulo[2], Douglas R. Call[2,4]

Brooke M. Ramay, Pharm D[¥]

1) Center for Health Studies, Universidad del Valle de Guatemala,

181 Avenida 11-95 Guatemala City, Guatemala, 01015

2) Paul G. Allen School for Global Animal Health, Washington State University

Pullman, WA 99164, United States

Mark, A. Caudell, PhD[¥]

2) Paul G. Allen School for Global Animal Health, Washington State University

Pullman, WA 99164, United States

3)Food and Agriculture Organization of the United Nations, United Nations Complex

UN Avenue, Gigiri, Nairobi. PO Box: 30470, 00100

Celia Cordon-Rosales, B.S.*

1) Center for Health Studies, Universidad del Valle de Guatemala,

18 Avenida 11-95 Guatemala City, Guatemala, 01015

L. Diego Archila, PhD

1) Center for Health Studies, Universidad del Valle de Guatemala,

18 Avenida 11-95 Guatemala City, Guatemala, 01015

Guy H. Palmer PhD
2) Paul G. Allen School for Global Animal Health, Washington State University
Pullman, WA 99164, United States

4) Nelson Mandela African Institution of Science and Technology, P.O.BOX 447 Arusha, Tanzania

Claudia Jarquin MSc
1) Center for Health Studies, Universidad del Valle de Guatemala,
18 Avenida 11-95 Guatemala City, Guatemala, 01015

Purificación Moreno MSc
1) Center for Health Studies, Universidad del Valle de Guatemala,
18 Avenida 11-95 Guatemala City, Guatemala, 01015

John P. McCracken DrPH
1) Center for Health Studies, Universidad del Valle de Guatemala,
18 Avenida 11-95 Guatemala City, Guatemala, 01015

Leah Rozenkrantz PhD
5) Department of Geography, Simon Fraser University, Burnaby V5A 1S6, British Columbia, Canada

Ofer AmramPhD
2) Paul G. Allen School for Global Animal Health, Washington State University
Pullman, WA 99164, United States

6) Elson S. Floyd College of Medicine, Washington State University, Spokane, PO Box 1495, WA, USA

Sylvia Omulo, PhD

2) Paul G. Allen School for Global Animal Health, Washington State University
Pullman, WA 99164, United States

Douglas R. Call PhD
2) Paul G. Allen School for Global Animal Health, Washington State University
Pullman, WA 99164, United States

4) Nelson Mandela African Institution of Science and Technology, P.O.BOX 447 Arusha, Tanzania

¥ Authors contributed equally
*corresponding author: ccordon@ces.uvg.edu.gt, (502) 2329-8452

Antibiotic use and hygiene interact to influence the distribution of antimicrobial-resistant bacteria in low-income communities in Guatemala

Household representative questionnaire Phase 1, UVG-WSU AMR study

Recruitment:

I. Hi, my name is ______ and I am here today with my workmate ______ we are here today because we are doing a study on antibiotic resistance. Would it be possible to speak with the head of household? Are you interested in hearing more about the study?
   a. Yes-Continue to question III
   b. No, the head of household is not here- go to question II
   c. No I do not want to hear anything else about the study -End

II. Because the head of household is not here currently, can we speak with a household representative?
   a. Yes- Identify the representative of the household in the absence of the head of household and continue with the representative of household in question III.
   b. No- It is not possible to speak with the household representative in the absence of the head of household
   c. No, there is no one here that represents the household- Ask if you can come back in one hour or make an appointment to return to speak with the head of household.

III. Do you mind if we take a minute of your time to see if you can participate in the study?
   a. Unwilling- End
   b. Willing- continue to read the introductory paragraph from informed consent to give potential participant more information about study and proceed to screening questions

Antibiotic use and hygiene interact to influence the distribution of antimicrobial-resistant bacteria in low-income communities in Guatemala

Household representative questionnaire Phase 1, UVG-WSU AMR study

Census and household screening

1. How many adults (> 18 years) live in this house?

2. For each adult identified in question 1, indicate the name of each person.

3. How many children from 0 to 4 years old live in this house?

4. For each child 0-4 years old in question 3, indicate the name of each child.

   (Explain that the Tablet will choose an adult and a child to ask the questionnaire. When selecting the participants, explain the study and ask for their consent. If the selected people are not there at the time, ask at what time you can find them and explain which will).

5. Has the selected adult lived in the household for at least one year?
   a. Yes
   b. No- Select another adult to participate.

6. Will the selected adult live in the same household for at least one more year?
   a. Yes
   b. No-Select another adult to participate.

7. Was informed consent obtained for the adult?
   a. Yes, proceed to the adult questionnaire at the end of the household representative questionnaire
   b. No, adult was not available - (ask at what time you can return). Ask for phone number.
   c. No, adult not interested in participating - (The adult will not be included in the study, perform the random adult selection process again.)

8. Has the selected child lived in the house for at least one year?
   a. Yes
   b. No- Select another child to participate.

9. Will the selected child live in the same household for at least one more year?
   a. Yes
   b. No- Select another child to participate.

10. Was informed consent obtained for the child?
    a. Yes, proceed to the child/guardian questionnaire after completing the household representative questionnaire
    b. No, legal guardian and or child were not available (field team will return).
    c. No, no interest in participating - Will not include the child in the study, perform the child randomization process again.

Antibiotic use and hygiene interact to influence the distribution of antimicrobial-resistant bacteria in low-income communities in Guatemala

Household representative questionnaire Phase 1, UVG-WSU AMR study

Demographic information

1. Code/identification (xxx-xx-2017), including GPS point, phone number

2. Location
    a. Coatepeque (drop down list)
        a. List Municipality
            2.a.a.1. List community
    b. San Juan Ostuncalco (drop down list)
        a. List Municipality
            2.b.a.1. List Community
3. Sex
    a. Male
    b. Female

4. How old are you?
    whole number in years

5. Is the household representative the head of Household
    a. Yes (go to Q7)
    b. No (go to Q6)

6. Relationship to head of household
    a. Mother/father
    b. Wife/husband
    c. Brother/sister
    d. Brother in law/sister in law
    e. Son/daughter
    f. Grandson/Granddaughter

7. Self-reported ethnicity
    a. Indigenous (go to Q8)
    b. Ladino
    c. Other, Specify
    d. Unknown
    e. Refused to answer

8. Specify self-reported indigenous group
    a. Xinca
    b. Mam
    c. Q'eqchi
    d. Other

9. What language do your household members usually speak at home?
    a. Spanish
    b. Xinca
    c. Mam
    d. Q'eqchi
    e. Other

f. refused

10. Do you know how to read and write?
    a. Yes
    b. No
    c. Refused

11. What is the highest level of education that you have completed?
    a. No formal education (has not been to school)
    b. Less than primary education complete
    c. Primary education complete
    d. Secondary education complete
    e. Higher than secondary education complete
    f. Other, specify___
    g. Refused

12. Does your household have: *(All items are direct questions)*

Item	Yes	No	Don't know	Refused
Cell phone				
TV				
Transportation				
A car or truck?				
Appliances				
A radio?				
A refrigerator?				
A microwave?				
A clothes dryer?				
A clothes washer				
Communication				
A household telephone?				

13. What is the monthly family income?
    a. <Q1,000.00
    b. Q1,001 to Q3,000
    c. Q3,001 to Q5,000
    d. d.>Q5,000.00
    e. Does not know
    f. Refuses to answer

Antibiotic use and hygiene interact to influence the distribution of antimicrobial-resistant bacteria in low-income communities in Guatemala

Household representative questionnaire Phase 1, UVG-WSU AMR study

II. Environmental factors: Water sources, construction of household structure, waste management

INTERVIEWER READ: Now I would like to ask you a few questions about water sources in your household, construction materials, and waste management. I would like to start by asking you questions about the drinking water available in your household.

14. Study personnel responds: is the questionnaire being conducted in the rainy season?
    a. Yes (go to question 15)
    b. No (go to question 16)

15. In the rainy season, what is the main source of drinking water for members of your household? (CHECK ONE, the main source, and proceed to question 18)
    a. Bottled water or sachet/bag water (“agua pura”)
    b. Tanker-truck
    c. Rainwater collection—private
    d. Rainwater collection—public
    e. Surface water (river, dam, lake, pond, stream, canal, irrigation channels)
    f. Piped water into dwelling (private tap)
    g. Piped water to yard/plot (private tap)
    h. Public tap/standpipe (public tap)
    i. Tubewell/borehole—private
    j. Tubewell/borehole—public
    k. Protected dug well–private
    l. Protected dug well–public
    m. Unprotected dug well—private
    n. Unprotected dug well—public
    o. Protected spring—private
    p. Protected spring—public
    q. Unprotected spring—private
    r. Unprotected spring—public
    s. Water from a tank/drum
    t. Other (specify) ____________________
    u. Don’t know

16. Study personnel responds: Is the questionnaire being conducted in the dry season?
    a. Yes (go to question 20)
    b. No (go to question 21)

17. In the dry season, what is the main source of drinking water for members of your household? (CHECK ONE)
    a. Bottled water or sachet/bag water (“agua pura”)
    b. Tanker-truck
    c. Rainwater collection—private
    d. Rainwater collection—public
    e. Surface water (river, dam, lake, pond, stream, canal, irrigation channels)
    f. Piped water into dwelling (private tap)
    g. Piped water to yard/plot (private tap)
    h. Public tap/standpipe (public tap)
    i. Tubewell/borehole—private

j. Tubewell/borehole—public
k. Protected dug well–private
l. Protected dug well–public
m. Unprotected dug well—private
n. Unprotected dug well—public
o. Protected spring—private
p. Protected spring—public
q. Unprotected spring—private
r. Unprotected spring—public
s. Water from a tank/drum
t. Other (specify) ____________________
u. Don't know

18. Do you use containers dedicated specifically for drinking water in the household?
    a. Yes (Go to Q19)
    b. No (Go to Q25)
    c. Don't know (Go to Q25)
    d. Refused (Go to Q25)

19. How are containers stored when they are **filled** with water?
    a. Covered,
    b. Uncovered

20. Where are containers stored when they are **filled** with water?
    a. Inside
    b. Outside

21. How are these containers stored when they are **empty**?
    a. Covered
    b. Uncovered

22. Where are these containers stored when they are **empty**
    a. Inside
    b. Outside

23. Are the storage containers cleaned before they are used?
    a. Yes (go to 24)
    b. No (go to 25)
    c. Don't know (go to 25)
    d. Refused (go to 25)

24. How are the storage containers cleaned? (mark all that apply)
    a. Soap and Water
    b. Disinfectant
    c. Soap, water and disinfectant
    d. The container is boiled in water
    e. Don´t know
    f. Refused

25. Do you ever treat your water in any way to make it safer to drink? (We are not asking about treating your water for mosquitoes.)
    a. Yes (go to 26)
    b. No (go to 29)
    c. Don't know (go to 29)
    d. Refused (go to 29)

26. IF YES TO 25: What do you usually do to the water to make it safer to drink? (Only check more than one response if several methods are usually used together. for example, cloth filtration and chlorine)
    a. Boil
    b. Add bleach/chlorine
    c. Add iodine
    d. Strain it through a cloth
    e. Use a water filter (ceramic, sand, composite, etc.)
    f. Solar disinfection / SODIS
    g. Let it stand and settle
    h. Add a flocculent
    i. Other (specify) ____________________
    j. Don't know
    k. Refused

27. When was the last time you treated your drinking water using this method? (CHECK ONE)
    a. Today
    b. Yesterday
    c. More than one day ago but less than one week ago
    d. One to four weeks ago
    e. More than one month ago
    f. Other (specify) ____________________
    g. Don't know/don't remember
    h. Refused

28. How often do you treat your drinking water using this method? (CHECK ONE)
    a. Always (for all drinking water consumed in the household)
    b. Usually
    c. Sometimes
    d. Never
    e. Don't know/don't remember
    f. Refused

We have finished talking about water to take, now let's talk about water used for activities in the house.

29. Do you use the river/creek water in your house?
    a. Yes (go to Q30)
    b. No (go to Q31)
    c. Don't know (go to Q31)
    d. Refused (go to Q31)

30. What do you use the river/creek water for? (select all that apply)
    a. To cook
    b. To wash the dishes
    c. To wash clothes
    d. To bathe
    e. To water the garden
    f. Don't know
    g. Refused

31. What kind of toilet facility or latrine, if any, do members of your household use most often? (CHECK ONE)
    a. Flush or pour/flush toilet or latrine
    b. Pit latrine with slab
    c. Pit latrine without slab/open pit
    d. No facilities or bush or field or plastic bag
    e. Composting/dry toilet or latrine
    f. Service or bucket latrine (where feces are manually removed)
    g. Ventilated improved pit latrine (VIP) Shared toilet facilities Hanging toilet/hanging latrine
    h. Other
    i. Don't know
    j. Refused

32. If flush or pour/flush toilet or latrine: Where is the waste flushed? (CHECK ONE)
    a. Flushed to piped sewer system
    b. Flushed to septic tank
    c. Flushed to pit latrine
    d. No Drainage/overflows to the floor
    e. Flushed to elsewhere
    f. Flushed to unknown place/not sure/don't know where
    g. Don´t know
    h. Other____

33. Where is the toilet/latrine located? (CHECK ONE)
    a. Inside or attached to dwelling
    b. Elsewhere on premises
    c. Outside premises
    d. Don't know
    e. Refused
    f. Other___

34. Do you share this toilet/latrine or defecation field with other households?
    a. Yes (go to Q35)
    b. No (go to Q 36)
    c. Don´t know (go to Q36)
    d. Refused (go to Q36)

35. Is this toilet/latrine or defecation field used as a public place to go to the bathroom?
    a. Yes

b. No
c. Don´t know
d. Refused

36. Is the toilet/latrine ever cleaned?
    a. Yes (go to Q 37)
    b. No (go to Q 38)
    c. Don´t know (go to Q 38)
    d. Refused (go to Q 38)

37. If the toilet/latrine is ever cleaned, when was the toilet/latrine last cleaned?
    a. Today
    b. Yesterday
    c. More than 1 day ago but less than 1 week ago
    d. 1-4 weeks ago
    e. More than one month ago
    f. Don´t know
    g. Refused

38. The last time [NAME OF YOUNGEST CHILD] passed stool, what was done to dispose of the stools? (CHECK ONE)
    a. Child used toilet/latrine
    b. Put/rinsed into toilet or latrine
    c. Put/rinsed into drain or ditch
    d. Thrown into garbage
    e. Buried
    f. Left in the open in yard
    g. Left in the open outside premises
    h. Other
    i. Don’t know
    j. Refused

39. OBSERVE—WHAT IS THE <u>Main</u> material of the household floor? (CHECK ONE)
    a. Natural floor – earth/sand
    b. Finished floor – ceramic tiles
    c. Finished floor – cement
    d. Natural floor – dung
    e. Natural floor – straw/saw dust
    f. Rudimentary floor – wood planks
    g. Rudimentary floor – palm/bamboo
    h. Finished floor – parquet or polished wood
    i. Finished floor – vinyl or asphalt strips
    j. Finished floor – carpet
    k. Other (specify) ________________________________

40. Observe: Is there animal excrement on the floor of the house?
    a. Yes
    b. No
    c. Not possible to determine

41. OBSERVE—WHAT IS THE Main material of the household roof? (CHECK ONE)
    a. No roof
    b. Natural roofing – thatch/palm leaf
    c. Natural roofing – sod
    d. Rudimentary roofing – rustic mat
    e. Rudimentary roofing – palm/bamboo
    f. Rudimentary roofing – wood planks
    g. rudimentary roofing – carton/cardboard
    h. Finished roofing – metal
    i. Finished roofing – wood
    j. Finished roofing – calamine/cement fiber
    k. Finished roofing – ceramic tiles
    l. Finished roofing – cement/concrete
    m. Finished roofing – roofing shingles
    n. finished roofing – plastic
    o. finished roofing – LAMINA OF ASBESTOS (DURALITE)
    p. Other (specify) _______________________________

42. OBSERVE—WHAT IS Main material of the household walls? (CHECK ONE)
    a. No walls
    b. Natural walls – cane/palm/trunk
    c. Natural walls – dirt
    d. Rudimentary walls – MUD BRICK
    e. Rudimentary walls – bamboo with mud
    f. Rudimentary walls – stone with mud
    g. Rudimentary walls – uncovered adobe
    h. Rudimentary walls – plywood
    i. Rudimentary walls – carton/cardboard
    j. Rudimentary walls – reused wood
    k. Finished walls – cement
    l. Finished walls – stone with lime/cement
    m. Finished walls – bricks
    n. Finished walls – cement blocks
    o. Finished walls – covered adobe
    p. Finished walls – wood planks/shingles
    q. Other (specify) _______________________________

43. How many households does the GPS door share?
    Whole number

44. How many bedrooms does the household of the respondent have?
    Whole number

45. Is the kitchen separated from the sleeping quarters
    a. Yes
    b. No

46. Trash disposal- How do you dispose of the trash in your household? (can choose more than one)
    a. It is burned
    b. It is burned as fuel
    c. It is recycled
    d. It is buried
    e. It is collected to be taken to the trash-dump
    f. Doesn´t know
    g. Refuses to answer

47. Do you use Wood stove, gas or both to cook?
    a. Wood stove
    b. Gas
    c. Both
    d. Neither (go to Q49)
    e. Doesn´t know (go to Q49)
    f. Refuses to answer

48. If you use a Wood stove, what type of stove do you generally use to cook? choose one
    a. Chimney and stove
    b. Stove over an open fire
    c. Other (specify) ____________________
    d. Don't know
    e. *No answer*

49. Do you wash your cooking/eating utensils before using them?
    a. Yes (go to Q50)
    b. No (go to Q51)
    c. Don't know (go to Q51)
    d. Refuses (go to Q51)

50. How do you wash the dishes?
    a. Soap and Water
    b. Disinfectant
    c. Soap, water and disinfectant
    d. Boiled
    e. Don´t know
    f. Don´t answer

51. What is the approximate age of the person who normally washes the dishes?
    a. 0-5 yo
    b. 6-12 yo
    c. 13-18 yo
    d. 19 or older
    e. Don't know
    f. Refused

III. Household practices: Hand washing

Antibiotic use and hygiene interact to influence the distribution of antimicrobial-resistant bacteria in low-income communities in Guatemala

Household representative questionnaire Phase 1, UVG-WSU AMR study

INTERVIEWER READ: Now I would now like to ask you questions about hygiene. These questions will be primarily about hand washing practices in your household.

52. Please mention all the places that members of this household could wash their hands at in the last two weeks **(CHECK ALL THAT APPLY)**
    a. In or near toilet facility inside household
    b. In or near kitchen on premises
    c. Close to where the animals are penned up
    d. Elsewhere on premises
    e. Outside premises
    f. Other
    g. Never wash hands / there is no place to do it
    h. Don't know
    i. Refused

IV. Household practice including ownership of, Animals, Cooking and diet

Section 3 *INTERVIEWER READ:* Thank you for your time. Now I want to ask you some questions about various conditions in your home. These are questions we ask every household.

53. Do members of this household own any free-ranging domestic animals? *(CHECK ONE)*
    a. Yes (go to Q54)
    b. No (go to Q57)
    c. Don’t know (go to Q57)
    d. Refused

54. *IF YES TO Q54:* What animals are owned? *(CHECK ALL THAT APPLY, use whole numbers and then go to Q55)*
    a. Dogs, ____ indicate quantity
    b. Cats, ____ indicate quantity
    c. Chickens, ____ indicate quantity
    d. Turkeys, ____ indicate quantity
    e. Geese, ____ indicate quantity
    f. Other birds (specify),____________________ indicate quantity
    g. Pigs, ____ indicate quantity
    h. Cows, ____ indicate quantity
    i. Sheep, ____ indicate quantity
    j. Goats ____ indicate quantity
    k. Other animals (specify) __________________ ____ indicate quantity
    l. Don’t know
    m. Refused

In the next section of the survey we will be asking you about medication use, specifically the use of antibiotics in your animals. Antibiotics are defined as medications used to kill bacteria. Bacteria are microorganisms that cause infections. Antibiotics are sold in pharmacies in Guatemala and sometimes in tiendas and supermarkets. The ministry of health also provides antibiotics to those that have a prescription from an MOH doctor. Common antibiotics are amoxicillin and tetracycline (show examples taken from ministry of health and nearest “tienda”) and are often recommended for use after visiting a doctor.

Sometimes, people choose to take antibiotics without a doctor’s or Vet’s recommendation.

55. Have you ever given antibiotics to your animals?
    a. Yes (Go to Q56, specify antibiotic use)
    b. No (Go to Q57)
    c. Doesn´t know (Go to Q57)
    d. Refuses to answer (Go to Q57)

56. Specify antibiotic use

	Medicine 1	Medicine 2	Medicine 3
Name of medicine	Name	Name	Name
• Medicine package bottle seen (yes/no)	Yes/No	Yes/No	Yes/No
• Medicine seen and name legible	Yes/No	Yes/No	Yes/No
•	Yes/No	Yes/No	Yes/No<br>o
Indicate which animal the antibiotic was given to	Dogs<br>Cats<br>Chickens<br>Turkeys<br>Geese<br>Other birds (specify)<br>________________<br>Pigs<br>Cows<br>Sheep<br>Goats<br>Other animals (specify)<br>_________________<br>_<br>Don’t know<br>Refused	Dogs<br>Cats<br>Chickens<br>Turkeys<br>Geese<br>Other birds (specify)<br>________________<br>Pigs<br>Cows<br>Sheep<br>Goats<br>Other animals (specify)<br>_________________<br>_<br>Don’t know<br>Refused	Dogs<br>Cats<br>Chickens<br>Turkeys<br>Geese<br>Other birds (specify)<br>________________<br>Pigs<br>Cows<br>Sheep<br>Goats<br>Other animals (specify)<br>_________________<br>_<br>Don’t know<br>Refused
How often do you give this medicine to your animals?	Number of times per year	Number of times per year	Number of times per year
Do you ever abstain from drinking animal milk or eating meat from the indicated animal after giving them medications?	Yes/No	Yes/No	Yes/No
For how long did you abstain?	No. of days	No. of days	No. of days

57. Do you have a domestic animal that lives freely inside the house?
    a. Yes (go to Q58)
    b. No (go to Q61)

58. Indicate type and quantity of animals that live freely inside the house *(CHECK ALL THAT APPLY)*
    a. Dogs
    b. Cats
    c. Chickens
    d. Turkeys
    e. Geese
    f. Other birds (specify) _______________
    g. Pigs
    h. Cows
    i. Sheep
    j. Goats
    k. Other animals (specify) _________________
    l. Don’t know
    m. Refused

59. Are your animals sometimes kept inside the living quarters (in the day or the night)? *(CHECK ALL THAT APPLY)*
    a. Yes (go to Q60)
    b. No (go to Q61)
    c. Don't know (go to Q61)
    d. Doesn't respond (go to Q61)

60. What animals are sometimes kept inside the living quarters (in the day or the night, where members of the family sleep)? *(CHECK ALL THAT APPLY)*
    a. Dogs
    b. Cats
    c. Chickens
    d. Turkeys
    e. Geese
    f. Other birds (specify) _______________
    g. Pigs
    h. Cows
    i. Sheep
    j. Goats
    k. Other animals (specify) _________________
    l. Don’t know
    m. Refused

61. Do you ever eat meat in this household?
    a. Yes (go to 62)
    b. No (go to Q64)
    c. Don’t know (go to Q64)

d. Refused (go to Q64)

62. What type of meat do you eat in this household? (May select more than one)
    a. Chicken
    b. Pork
    c. Beef
    d. Fish
    e. Rabbit
    f. Turkey
    g. Duck
    h. Other, specify
    i. Don’t know
    j. Refused

63. In a verification list, please indicate where each type of meat selected in Q62 came from:
    a. Raised at home
    b. Purchased at the market
    c. Purchased at the supermarket
    d. Purchased at small grocery store
    e. Purchased at the tienda
    f. Other, specify
    g. Don’t know
    h. Refused

64. Do you drink milk in this household ?
    a. Yes (go to question 65)
    b. No (END)
    c. Don’t know (END)
    d. Refused (go to END)

65. What type of milk do you drink in this household? (CHECK ALL THAT APPLY)
    a. Cow's milk (fresh) (go to Q66)
    b. Powdered milk (go to Q68)
    c. Boxed mil (go to Q68)
    d. Goat's milk (fresh) (go to Q66)
    e. Milk in bag (go to Q68)
    f. Other (go to Q68)

66. Do you treat (or prepare) milk in any way before you drink it?
    a. Yes (go to Q67)
    b. No (go to Q68)
    c. Do not know (go to Q68)
    d. Refuses to respond (go to Q68)
67. Do you boil the milk before taking it?
    a. Yes (go to Q68)
    b. No (go to Q68)
    c. Do not know (go to Q68)
    d. Refuses to respond (go to Q68)

68. Do you store milk in this household?
    a. Yes (go to Q69)
    b. No (END)
    c. Don´t know (END)
    d. No response (END)

69. Where do you store the milk?
    a. In the refrigerator
    b. Not in the refrigerator?
    c. Don't know
    d. No response

70. What type of container do you use to store your milk?
    a. In your original disposable container (purchased at the store / supermarket) (END)
    b. In a plastic container (go to Q71)
    c. In a container made of metal (go to Q71)
    d. In a glass container (go to Q71)
    e. In a bag (go to Q71)
    f. Don't know (END)
    g. Don't answer (END)

71. Is it a container that is specifically used for milk?
    a. Yes (go to Q72)
    b. No (END)
    c. Don't' know (END)
    d. No response (END)

72. Do you wash the container before putting milk in it?
    a. Yes (go to Q73)
    b. No (END)
    c. Don't know (END)
    d. Don't answer (END)

73. How do you wash the container? (choose all that apply)
    a. Soap and Water
    b. Disinfectant
    c. Soap, water and disinfectant
    d. Boiled
    e. Don´t know
    f. Don´t answer

--End

Antibiotic use and hygiene interact to influence the distribution of antimicrobial-resistant bacteria in low-income communities in Guatemala

Household representative questionnaire Phase 2, UVG-WSU AMR study

Recruitment:

I. Hi, my name is ______ and I am here today with my workmate ______. We are here today because we are conducting the second phase of an antimicrobial resistance study. Your household was included in the first phase of this study. Would it be possible to speak with _______(the name of the representative of the household who we interviewed previously)?
   a. Yes-Continue to question 1
   b. No, the head of household is not here, determine time to come back to speak with the person who was previously interviewed
   c. No I do not want to participate in the second phase of the study -End

Antibiotic use and hygiene interact to influence the distribution of antimicrobial-resistant bacteria in low-income communities in Guatemala

Household representative questionnaire Phase 2, UVG-WSU AMR study

Questions regarding participant selection criteria

1. How many people in total live in this household (including children, elderly people, handicapped people, etc.)?

2. Please list the people who live in this household, including their gender and the age of each

    a. Person I-X
        A. Male/Female
        B. Age (numeric from 0 to 100)

Antibiotic use and hygiene interact to influence the distribution of antimicrobial-resistant bacteria in low-income communities in Guatemala

Household representative questionnaire Phase 2, UVG-WSU AMR study

Questionnaire for the Community-based Assessment of Antibiotic Resistant *Escherichia coli* in Quetzaltenango, Guatemala

I. DEMOGRAPHIC INFORMATION OF HOUSEHOLD REPRESENTATIVE

1. Code/identification (xxx-xx-2017), including GPS point, phone number

2. Location
   a. Coatepeque (drop down list)
      a. List Municipality
         2.a.a.1. List community
   b. San Juan Ostuncalco (drop down list)
      a. List Municipality
         2.b.a.1. List Community

3. Does your household have: *(All items are direct questions)*

Item	Yes	No	Don't know	Refused
Electricity				
Computer				

II. Animal Ownership

4. Do members of this household own any free-ranging domestic animals? *(CHECK ONE)*
   a. Yes (go to Q5)
   b. No (go to Q8)
   c. Don't know (go to Q8)
   d. Refused

5. What animals are owned? *(CHECK ALL THAT APPLY, use whole numbers and then go to Q6)*
   a. Dogs, ____ indicate quantity
   b. Cats, ____ indicate quantity
   c. Chickens, ____ indicate quantity
   d. Turkeys, ____ indicate quantity
   e. Geese, ____ indicate quantity
   f. Other birds (specify),____________________ indicate quantity
   g. Pigs, ____ indicate quantity
   h. Cows, ____ indicate quantity
   i. Sheep, ____ indicate quantity
   j. Goats ____ indicate quantity
   k. Other animals (specify) __________________ ____ indicate quantity
   l. Don't know
   m. Refused

In the next section of the survey we will be asking you about medication use, specifically the use of antibiotics in your animals. Antibiotics are defined as medications used to kill bacteria. Bacteria are microorganisms that cause infections. Antibiotics are sold in

pharmacies in Guatemala and sometimes in tiendas and supermarkets. The ministry of health also provides antibiotics to those that have a prescription from an MOH doctor. Common antibiotics are amoxicillin and tetracycline (show examples taken from ministry of health and nearest "tienda") and are often recommended for use after visiting a doctor. Sometimes, people choose to take antibiotics without a doctor's or Vet's recommendation.

6. In the last 6 months have you given antibiotics to your animals?
   a. Yes (Go to Q7, specify antibiotic use)
   b. No (Go to Q8)
   c. Doesn´t know (Go to Q8)
   d. Refuses to answer (Go to Q8)

7. Specify antibiotic use

	Medicine 1	Medicine 2	Medicine 3
Name of medicine	Name	Name	Name
• Medicine package bottle seen (yes/no)<br>• Medicine seen and name legible	Yes/No	Yes/No	Yes/No
	Yes/No	Yes/No	Yes/No
	Yes/No	Yes/No	Yes/No<br>o
Indicate which animal the antibiotic was given to	Dogs<br>Cats<br>Chickens<br>Turkeys<br>Geese<br>Other birds (specify)<br>_________________<br>Pigs<br>Cows<br>Sheep<br>Goats<br>Other animals (specify)<br>__________________<br>_<br>Don't know<br>Refused	Dogs<br>Cats<br>Chickens<br>Turkeys<br>Geese<br>Other birds (specify)<br>_________________<br>Pigs<br>Cows<br>Sheep<br>Goats<br>Other animals (specify)<br>__________________<br>_<br>Don't know<br>Refused	Dogs<br>Cats<br>Chickens<br>Turkeys<br>Geese<br>Other birds (specify)<br>_________________<br>Pigs<br>Cows<br>Sheep<br>Goats<br>Other animals (specify)<br>__________________<br>_<br>Don't know<br>Refused
How often do you give this medicine to your animals?	Number of times per year	Number of times per year	Number of times per year
Do you ever abstain from drinking animal milk or eating meat from the indicated animal	Yes/No	Yes/No	Yes/No

after giving them medications?			
For how long did you abstain?	No. of days	No. of days	No. of days

8. Do you have a domestic animal that lives freely inside the house?
    a. Yes (go to Q9)
    b. No – End of questionnaire

9. Indicate type and quantity of animals that live freely inside the house *(CHECK ALL THAT APPLY)*
    a. Dogs
    b. Cats
    c. Chickens
    d. Turkeys
    e. Geese
    f. Other birds (specify) ________________
    g. Pigs
    h. Cows
    i. Sheep
    j. Goats
    k. Other animals (specify) __________________
    l. Don't know
    m. Refused

10. Are your animals sometimes kept inside the living quarters (in the day or the night)? *(CHECK ALL THAT APPLY)*
    a. Yes (go to Q11)
    b. No (go to Q12)
    c. Don't know (go to Q11)
    d. Doesn't respond (go to Q12)

11. What animals are sometimes kept inside the living quarters (in the day or the night, where members of the family sleep)? *(CHECK ALL THAT APPLY)*
    a. Dogs
    b. Cats
    c. Chickens
    d. Turkeys
    e. Geese
    f. Other birds (specify) ________________
    g. Pigs
    h. Cows
    i. Sheep
    j. Goats
    k. Other animals (specify) __________________
    l. Don't know
    m. Refused

12. In the last 6 months have you given antibiotics to your that live inside the household?

a. Yes (Go to Q13, specify antibiotic use)
b. No END of questionnaire
c. Doesn´t know END of questionnaire
d. Refuses to answer END of questionnaire

13. Specify antibiotic use

	Medicine 1	Medicine 2	Medicine 3
Name of medicine	Name	Name	Name
• Medicine package bottle seen (yes/no)<br>• Medicine seen and name legible	Yes/No	Yes/No	Yes/No
	Yes/No	Yes/No	Yes/No
	Yes/No	Yes/No	Yes/No<br>o
Indicate which animal the antibiotic was given to	Dogs<br>Cats<br>Chickens<br>Turkeys<br>Geese<br>Other birds (specify)<br>________________<br>Pigs<br>Cows<br>Sheep<br>Goats<br>Other animals (specify)<br>_________________<br>_<br>Don't know<br>Refused	Dogs<br>Cats<br>Chickens<br>Turkeys<br>Geese<br>Other birds (specify)<br>________________<br>Pigs<br>Cows<br>Sheep<br>Goats<br>Other animals (specify)<br>_________________<br>_<br>Don't know<br>Refused	Dogs<br>Cats<br>Chickens<br>Turkeys<br>Geese<br>Other birds (specify)<br>________________<br>Pigs<br>Cows<br>Sheep<br>Goats<br>Other animals (specify)<br>_________________<br>_<br>Don't know<br>Refused
How often do you give this medicine to your animals?	Number of times per year	Number of times per year	Number of times per year
Do you ever abstain from drinking animal milk or eating meat from the indicated animal after giving them medications?	Yes/No	Yes/No	Yes/No
For how long did you abstain?	No. of days	No. of days	No. of days

--End

Antibiotic use and hygiene interact to influence the distribution of antimicrobial-resistant bacteria in low-income communities in Guatemala

Household participant questionnaire, Phase 1 UVG-WSU AMR study

Recruitment:

I. Hi, my name is [Name of person speaking] and I am here today with my workmate [name of workmate]. We are here today because we are doing a study on antibiotic resistance. Would it be possible to speak with [person selected for study] to see if they may be interested in hearing more about the study.
   a. Yes-Continue to II, informed consent
   b. No-[Name of selected participant] is not here. Come back later
   c. No, you cannot speak with that person.

II. Do you mind if we take a minute of your time to determine if you can participate in the study?

   a. Yes- End
   b. No- continue to screening questions

Antibiotic use and hygiene interact to influence the distribution of antimicrobial-resistant bacteria in low-income communities in Guatemala

Household participant questionnaire, Phase 1 UVG-WSU AMR study

Screening Questions, for selected household potential participants: To be carried out by telephone or in person

1. Have you (your child) lived in the current household at least one year?
    a. Yes
    b. No- End

2. Will you (your child) live in the household during the next year (2017-18)?
    a. Yes
    b. No- End

3. Are you (your child) currently ill with acute diarrheal, febrile, or acute respiratory illness?
    a. Yes- End
    b. No

4. Are you (your child) currently taking some sort of medication today?
    a. Yes- End (go to Q5)
    b. No(go to Q6)

5. Can you *show* us which medication you (your child) is taking?
    a. Yes (go to Q6)
    b. No. (go to Q7)

6. Field worker response: Is the current medication an antibiotic?
    a. Yes- End
    b. No (go to Q9)

7. Can you *tell* us which medication you (your child) are taking?
    a. Yes
    b. No, End

8. Field worker response: Is the current medication mentioned an antibiotic?
    a. Yes- End
    b. No (go to Q9)

9. Are you (your child) willing to provide research team with a stool sample?
    a. Yes, proceed with informed consent.
    b. No-End

10. Informed consent granted
    a. Yes
    b. No-End

11. For children less than 5 yo, parental permission for child to participate given?
    a. Yes
    b. No-End

If the selected adult does not pass the screening questions, go to the next randomly selected adult within the household

Antibiotic use and hygiene interact to influence the distribution of antimicrobial-resistant bacteria in low-income communities in Guatemala

Household participant questionnaire, Phase 1 UVG-WSU AMR study

1 DEMOGRAPHIC INFORMATION OF HOUSEHOLD REPRESENTATIVE

1. Code/identification (xxx-xx-2017), GPS point, telephone number

2. Location
    a. Coatepeque (drop down list)
        a. List Municipality
            2.a.a.1. List community
    b. San Juan Ostuncalco (drop down list)
        a. List Municipality
            2.b.a.1. List Community
3. Sex of enrolled participant
    a. Male
    b. Female

4. What is your (your child´s) age (whole number in years)

5. What is your (your child´s) ethnicity
    a. Indigenous (go to Q6)
    b. Ladino
    c. Other
    d. Unknown
    e. Refused to answer

6. Specify indigenous group
    a. Xinca
    b. Mam
    c. Q'eqchi
    d. Other

7. Do you (your child) know how to read and write?
    a. Yes
    b. No

8. What is the highest level of education that you (your child) have completed?
    a. No formal education (never went to school)
    b. Less than primary education complete
    c. Primary education complete
    d. Secondary education complete
    e. Higher than secondary education complete

9. For adult participants, how frequently do you consume alcohol?
    a. Does not drink alcohol
    b. Yearly
    c. Monthly
    d. Weekly

e. Daily
f. Refuses

I. Household practices: Hand washing

INTERVIEWER READ: Now I would now like to ask you questions about hygiene. These questions will be primarily about hand washing practices.

10. Have you (your child) washed your hands with water for any reason today?
    a. Yes (go to 11)
    b. No (go to Q13)
    c. Don't know (go to Q13)
    d. Refused to answer (go to Q13)

11. Where did the water come from that you (your child) used to wash your hands?
    a. With water from a faucet
    b. With standing water from a bucket
    c. Don't know
    d. Refused to answer

12. Did you (your child) use soap?
    a. Yes
    b. No
    c. Don't know
    d. Refused to answer

II. Antibiotic usage history

In the next section of the survey we will be asking you (your child) about medication use, specifically the use of antibiotics. Antibiotics are defined as medications used to kill bacteria. Bacteria are microorganisms that cause infections. Antibiotics are sold in pharmacies in Guatemala and sometimes in tiendas and supermarkets. The ministry of health also provides antibiotics to those that have a prescription from an MOH doctor. Common antibiotics are amoxicillin and tetracycline (show examples taken from ministry of health and nearest "tienda") and are often recommended for use after visiting a doctor. Sometimes, people choose to take antibiotics without a doctor's recommendation.

13. Have you (your child) ever used antibiotics?
    a. Yes (Go to Q14)
    b. No (skip to next section, Q20)
    c. Don't know (skip to next section, Q20)
    d. No response (skip to next section, Q20)

14. Have you (your child) taken antibiotics in the last 14 days:
    a. Yes (go to 15)
    b. No (go to 20)
    c. Don´t know (go to 20)

15. The last time you (your child) took an antibiotic what did you (your child) take it for (may select more than one)?
    a. Congestion
    b. Cough
    c. Head ache
    d. Fever
    e. Vomiting
    f. Diarrhea
    g. Skin wounds
    h. HIV
    i. Zika
    j. Dengue
    k. Chikungunya
    l. TB
    m. Otro
    n. Don't know
    o. Refused

16. Did you (your child) seek medical attention for this illness?
    a. Yes (go to Q17)
    b. No (go to Q18)
    c. Don't know (go to Q18)
    d. Don't respond (go to Q18)

17. Where did you (your child) seek medical attention? (select all that apply)
    a. Puesto de Salud
    b. Centro de Salud
    c. Public Hospital
    d. Private Hospital
    e. Private clinic Clínic
    f. Centro de Convergencia
    g. Community health care worker
    h. Health fair
    i. Pharmacy
    j. Tienda
    k. Natural medicine doctor
    l. From family members and/or friends
    m. Others, specify _____________
    n. Don’t know
    o. Does not answer

18. What medicine(s) did you (your child) use for this particular illness? (ask to be shown medication if still available. encourage “what else” until nothing further is mentioned and check all that apply)
    a. Antibiotics – go to Q19 and list antibiotics
    b. Antihelminthics/dewormers – go to Q20
    c. Antimalarials – go to Q20
    d. Pain-killers – go to Q20
    e. Herbs – go to Q20

f. Other – go to Q20
g. Don't know – go to Q20
h. Refused – go to Q20

Antibiotic use and hygiene interact to influence the distribution of antimicrobial-resistant bacteria in low-income communities in Guatemala

Household participant questionnaire, Phase 1 UVG-WSU AMR study

19. For each antibiotic list the following (then go to 100):

	Medicine 1	Medicine 2	Medicine 3
Name of medicine	Name	Name	Name
• Medicine package bottle seen (yes/no)<br>• Medicine seen and name legible<br>• Name available	Yes/No<br>Yes/No<br>Yes/No<br>Yes/No	Yes/No<br>Yes/No<br>Yes/No<br>Yes/No	Yes/No<br>Yes/No<br>Yes/No<br>Yes/No
Was this medicine prescribed to you (your child) at a health facility?<br>If YES, at which health facility was it recommended/ prescribed?<br>If NO, how did you (your child) know which medicine to buy?	Yes/No/ DK/Refused<br>Centro de salud MSPAS/Puesto de salud/Clínica privada<br>Amgiofamiliar/experiencia previa/ persona en la farmacia/médico/otro	Yes/No/ DK/Refused<br>Centro de salud MSPAS/Puesto de salud/Clínica privada<br>Amgiofamiliar/experiencia previa/ persona en la farmacia/médico/otro	Yes/No/ DK/Refused<br>Centro de salud MSPAS/Puesto de salud/Clínica privada<br>Amgiofamiliar/experiencia previa/ persona en la farmacia/médico/otro
Where did you (your child) get this medicine?	Pharmacy/Corner store(tienda)/Supermarket/MSPAS clinic/Friend or family member	Pharmacy/Corner store(tienda)/Supermarket/MSPAS clinic/Friend or family member	Pharmacy/Corner store(tienda)/Supermarket/MSPAS clinic/Friend or family member
Did you (your child) buy/receive a complete dose of the medicine?	Complete/partial dose/don´t know/refused	Complete/partial dose/don´t know/refused	Complete/partial dose/don´t know/refused
For how many days did you (your child) take this medication?	Number of days	Number of days	Number of days
How frequently do you (your child) take antibiotics?	Every month/Every 3 months/Every 6 months/every year/anytime it is necessary/Dont know/Dont respond	Every month/Every 3 months/Every 6 months/every year/anytime it is necessary/Dont know/Dont respond	Every month/Every 3 months/Every 6 months/every year/anytime it is necessary/Dont know/Dont respond
Did any other person also use this medication?<br>• If yes, who else used the medication	Yes/No<br>Specify	Yes/no<br>Specify	Yes/No<br>Specify

III. Household practices including possession of animals, methods of cooking and diet

20. Do you (your child) ever eat meat?
    a. Yes (go to Q21)
    b. No (go to Q23)
    c. Don’t know (go to Q23)
    d. Refused (go to Q23)

21. What type of meat? (May select more than one)
    a. Chicken

b. Pork
c. Beef
d. Fish
e. Rabbit
f. Turkey
g. Duck
h. Other, specify
i. Don’t know
j. Refused

22. In a verification list, please indicate where each type of meat selected in Q21 came from:
    a. Raised at home
    b. Purchased at the market
    c. Purchased at the supermarket
    d. Purchased at small grocery store
    e. Purchased at the tienda
    f. Other, specify
    g. Don’t know
    h. Refused

23. Do you (your child) drink milk?
    a. Yes (go to question 24)
    b. No (go to Q30)
    c. Don’t know (go to Q30)
    d. Refused (go to Q30)

24. What type of milk do you (your child) drink? (CHECK ALL THAT APPLY)
    a. Cow's milk (fresh) (go to P25)
    b. Powdered milk (go to P29)
    c. Boxed milk (go to P29)
    d. Goat's milk (fresh) (go to P25)
    e. Milk in bag (go to P25)
    f. Other (go to P25)

25. Do you (your child) treat (or prepare) milk in any way before you (your child) drink it?
    a. Yes (go to P26)
    b. No (go to P28)
    c. Do not know (go to P28)
    d. Refuses to respond (go to P28)

26. Do you (your child) boil the milk before taking it?
    a. Yes (go to P27)
    b. No (go to P28)
    c. Do not know (go to P28)
    d. Refuses to respond (go to P28)

27. How much boiled milk do you (your child) drink per week? (Number of whole cups/glasses)
    a. Numerical response
    b. Don´t know

c. Refused

28. How much un-boiled milk do you (your child) drink per week (number of whole cups/glasses)?
    a. Numerical response
    b. Don´t know
    c. Refused

29. How much milk purchased in the store / supermarket do you (your child) drink per week (number of whole cups/glasses)?
    a. Numerical response
    b. Don´t know
    c. Refused

IV. FACTORS RELATED TO TRAVELING

30. In the past 14 days, have you (your child) traveled outside of your municipality (inside your department)?
    a. Yes (go to Q31)
    b. No (go to Q34)

31. To which municipality?
    a. San Carlos Sija
    b. Cabricán
    c. Huitán
    d. Sibilia
    e. Cajolá
    f. Palestina de los Altos
    g. San Miguel Sigüila
    h. Ostuncalco
    i. San mateo
    j. San Francisco La Unión
    k. La Esperanza
    l. Olintepeque
    m. Salcajá
    n. Quetzaltenango
    o. Almolonga
    p. Cantel
    q. Zunil
    r. El Palmar
    s. Concepción Chiquirichapa
    t. San Martin Sacatepéquez
    u. Colomba
    v. Flores Costa Cuca
    w. Coatepeque
    x. Génova

32. How long were you (your child) away from home on this trip?
    a. One week or less
    b. More than one week, but less than a month
    c. One month or more

33. How often do you (your child) go to this location?
    Number (type in number) **times per** unit (week/month/year) drop down

34. In the last 14 days, have you (your child) gone to a department outside of Quetzaltenango?
    a. Yes (go to Q35)
    b. No (go to Q38)
    c. Don't know (go to Q38)
    d. Refused (go to Q38)

35. To which department?
    a. Alta Verapaz
    b. Baja Verapaz
    c. Chimaltenango
    d. Chiquimula
    e. El Petén
    f. El Progreso
    g. El Quiché
    h. Escuintla
    i. Guatemala
    j. Huehuetenango
    k. Izabal
    l. Jalapa
    m. Jutiapa
    n. Quetzaltenango
    o. Retalhuleu
    p. Sacatepéquez
    q. San Marcos
    r. Santa Rosa
    s. Sololá
    t. Suchitepéquez
    u. Totonicapán
    v. Zacapa

36. How long were you (your child) away from home on this trip?
    a. One week or less
    b. More than one week, but less than a month
    c. One month or more

37. How often do you (your child) go to this location?
    Number (type in number) **times per** unit (week/month/year) drop down

38. In the last 14 days, have you (your child) traveled to a country outside of Guatemala?
    a. Yes (go to Q39)
    b. No (go to Q42)

39. To which country?
    a. Mexico
    b. El Salvador

c. Honduras
d. Belize
e. The United States
f. Other, specify

40. In the last trip out of the country, how long were you (your child) away from home?
    a. One week or less
    b. Greater than one week, less than one month
    c. One month or greater

41. How often do you (your child) go to this location?
    Number (type in number) **times per** unit (week/month/year) drop down

V. **Previous illnesses and exposition to hospitals:** We are going to ask you (your child) now about previous episodes you (your child) may have had of diarrhea

42. Have you (your child) had diarrhea in the past 14 days?
    a. Yes (go to Q43)
    b. No (go to Q51)
    c. Don´t know (go to Q51)
    d. Refused to answer (go to Q51)

43. How many days ago did you (your child) have your (their) last episode of diarrhea/loose stool
    Numeric whole numbers
    If this person reports diarrhea today, they may not participate in the study

44. During your (their) worst day of diarrhea/loose stool (IN A 24 HOUR PERIOD) how many loose stools did you (your child) have?
    a. Numeric whole numbers
    b. Don´t know
    c. Refuses

If person had diarrhea:

45. During this episode of diarrhea/loose stools, did you (your child) have any vomiting symptoms?
    a. Yes
    b. No
    c. Don´t know
    d. Refused to answer

46. Did you (your child) seek any type of medical attention outside the home to cure your (their) diarrhea?
    e. Yes (go to Q47)
    f. No(go to Q48)
    g. Don´t know(go to Q48)
    h. Refused to answer(go to Q48)

47. If you (your child) did seek medical attention outside the home where did you (your child) seek help? (mark all that apply)
    a. Health Post
    b. Health Center
    c. Public hospital
    d. Private hospital
    e. Private clinic
    f. Convergence center
    g. Health worker
    h. Medical fair
    i. Pharmacy
    j. Store
    k. Tradition/natural medicine
    l. Family members
    m. Other, specify ________
    n. Refused to answer
    o. Don't know

48. Did you (your child) take any medicine to treat this episode of diarrhea/loose stools?
    a. Yes (go to Q 49)
    b. No (go to Q51)
    c. Don´t know (go to Q51)
    d. Refused to answer (go to Q51)

49. Which medication did you (your child) take
    a. Antibiotics (go to Q50)
    b. Painkillers (go to Q51)
    c. Anthelminthic(go to Q51)
    d. Herbal remedy, specify________(go to Q51)
    e. Other, specify ___________(go to Q51)
    f. Nothing (go to Q51)

50. Did you (your child) already tell us about the antibiotics you (your child) took to treat this diarrhea in Q75?
    a. Yes (go to Q51)
    b. No (go back to Q19 and fill out table, then continue with Q51)
    c. Don't know(go to Q51)
    d. Refused to answer (go to Q51)

51. Do you (your child) work in the hospital?
    a. Yes (continue with following questions, clarifying that we are asking about visits to the hospital outside of work)
    b. No

52. In the past three months have you (your child) visited the hospital for any reason?
    a. Yes (go to Q53)
    b. No (go to Q54)
    c. Refused to answer (go to Q54)
    d. d. Don't know (go to Q54)

53. Why did you (your child) visit the hospital?
    a. Reason unrelated to an illness
    b. Visit a patient who was hospitalized
    c. Healthy visit check up
    d. Outpatient treatment for your (your child´s) illness
    e. Acute treatment of your (your child´s) illness/hospitalization
    f. Other, specify____
    g. Don´t know
    h. Refused to answer

54. In the last three months have you (your child) visited a centro de salud for any reason?
    a. Yes (go to Q55)
    b. No (end)
    c. Refused to answer (end)
    d. Don't know (end)

55. Why did you (your child) go to the centro de salud?
    a. Reason unrelated to an illness
    b. Visit a patient who was hospitalized
    c. Healthy visit check up
    d. Outpatient treatment for illness
    e. Acute illness/hospitalization
    f. Other, specify____
    g. Don´t know
    h. Refused to answer

---END

Antibiotic use and hygiene interact to influence the distribution of antimicrobial-resistant bacteria in low-income communities in Guatemala

Household participant questionnaire, Phase 1 UVG-WSU AMR study

Antibiotic use and hygiene interact to influence the distribution of antimicrobial-resistant bacteria in low-income communities in Guatemala

Household participant questionnaire, Phase 2 UVG-WSU AMR study

Recruitment:

1. Hi, my name is [Name of person speaking] and I am here today with my workmate [name of workmate]. We are here today because we are conducting the second phase of an antimicrobial resistance study. Your household was included in the first phase of this study. Would it be possible to speak with [person selected for study] to see if they are willing to complete the second phase of the study including collection of the fecal sample.
   a. Yes-Continue to 1
   b. No-[Name of selected participant] is not here, determine what time you can return to speak the previously selected participant.
   c. No, the previously selected participant declines to participate in the second phase of the study

Screening Questions, phase 2 participant:

1. Are you (your child) currently ill with symptoms of diarrhea, fever, or severe respiratory illness?
   a. Yes- End
   b. No

2. Are you (your child) currently taking some sort of medication today?
   a. Yes- End (go to Q3)
   b. No(go to Q7)

3. Can you *show* us which medication you (your child) is taking?
   a. Yes (go to Q4)
   b. No. (go to Q5)

4. Field worker response: Is the current medication an antibiotic?
   a. Yes- End
   b. No (go to Q7)

5. Can you *tell* us which medication you (your child) are taking?
   a. Yes (go to Q6)
   b. No, End

6. Field worker response: Is the current medication mentioned an antibiotic?
   a. Yes- End
   b. No (go to Q7)

7. Are you (your child) willing to provide research team with a stool sample?
   a. Yes, proceed with verbal consent for phase 2.
   b. No-End

8. Are you (your child) willing to participate in the second phase of the study?
   a. Yes, proceed with questionnaire and sample collection.
   b. No-End

Antibiotic use and hygiene interact to influence the distribution of antimicrobial-resistant bacteria in low-income communities in Guatemala

Household participant questionnaire, Phase 2 UVG-WSU AMR study

DEMOGRAPHIC INFORMATION

1. Code/identification (xxx-xx-2017), GPS point, telephone number

2. Location
   a. Coatepeque (drop down list)
      a. List Municipality
         2.a.a.1. List community
   b. San Juan Ostuncalco (drop down list)
      a. List Municipality
         2.b.a.1. List Community
3. Sex of enrolled participant
   a. Male
   b. Female

I. Previous illnesses: We are going to ask you now about previous episodes you (your child) may have had of diarrhea, respiratory illness and fever

4. Have you (your child) had diarrhea in the past 14 days?
   a. Yes (go to Q5)
   b. No (go to Q7)
   c. Don´t know (go to Q7)
   d. Refused to answer (go to Q7)

5. Did you (your child) take any medicine to treat this episode of diarrhea/loose stools?
   a. Yes (go to Q 6)
   b. No (go to Q7)
   c. Don´t know (go to Q7)
   d. Refused to answer (go to Q7)

6. Which medication did you (your child) take to treat the diarrhea/loose stools?
   a. Antibiotics
   b. Painkillers
   c. Anthelminthic
   d. Herbal remedy, specify
   e. Other, specify ___________
   f. Nothing

7. Have you (your child) had a fever in the past 14 days?
   a. Yes (go to Q8)
   b. No (go to Q9)
   c. Don´t know (go to Q9)
   d. Refused to answer (go to Q9)

8. Did you (your child) take any medicine to treat this episode of fever?
   a. Yes (go to Q 9)
   b. No (go to Q10)
   c. Don´t know (go to Q10)
   d. Refused to answer (go to Q10)

9. Which medication did you (your child) take to treat the fever?
    a. Antibiotics
    b. Painkillers
    c. Anthelminthic
    d. Herbal remedy, specify
    e. Other, specify ___________
    f. Nothing

10. Have you (your child) had a flu/cold, or a cough with rapid breathing and agitated breathing or difficulty in breathing associated with chest pain in the past 14 days?
    a. Yes (go to Q11)
    b. No (go to Q13)
    c. Don´t know (go to Q13)
    d. Refused to answer (go to Q13)

11. Did you (your child) take any medicine to treat this episode of cold/flu?
    a. Yes (go to Q 12)
    b. No (go to Q13)
    c. Don´t know (go to Q13)
    d. Refused to answer (go to Q13)

12. Which medication did you (your child) take for the cold/flu
    a. Antibiotics
    b. Painkillers
    c. Anthelminthic
    d. Herbal remedy, specify
    e. Other, specify ___________
    f. Nothing

II. Antibiotic usage history

In the next section of the survey we will be asking you (your child) about medication use, specifically the use of antibiotics. Antibiotics are defined as medications used to kill bacteria. Bacteria are microorganisms that cause infections. Antibiotics are sold in pharmacies in Guatemala and sometimes in tiendas and supermarkets. The ministry of health also provides antibiotics to those that have a prescription from an MOH doctor. Common antibiotics are amoxicillin and tetracycline (show examples taken from ministry of health and nearest "tienda") and are often recommended for use after visiting a doctor. Sometimes, people choose to take antibiotics without a doctor's recommendation.

13. Did you (your child) save the bag for medications that we gave you the last time we were here?
    a. Yes, please bring the bag of medications so that we can document what you (your child) have taken (go to Q14)
    b. No (go to Q15)
    c. Don´t know (go to Q15)
    d. No response (go to Q15)

14. Written documentation of medications: Please write down the information from each package of medication:
    a. Brand name
    b. Generic name
    c. Dosage form (Tablet/capsule/injectable/liquid/suppository)
    d. Dose

Now I will ask you (your child) a couple of questions about the antibiotics that are in the bag (INTERVIEWER verifies by checking medications with antibiotic list); if there is no bag or if in the bag there are no antibiotics, I would like to ask you (your child) if you (they) have taken any antibiotics in the last 6 months (show antibiotic album)

15. Have you (your child) taken antibiotics in the past 6 months (Including the antibiotics in the bag)?
    a. Yes (Go to Q16)
    b. No (skip to next section, Q17)
    c. Don't know (skip to next section, Q17)
    d. No response (skip to next section, Q17)

16. For each antibiotic list the following (then go to 100):

	Medicine 1	Medicine 2	Medicine 3
Name of medicine	Name	Name	Name
• Medicine package bottle seen (yes/no)	Yes/No	Yes/No	Yes/No
• Medicine seen and name legible	Yes/No	Yes/No	Yes/No
• Name available	Yes/No<br>Yes/No	Yes/No<br>Yes/No	Yes/No<br>Yes/No
What did you (your child) take the medication for?	a. Congestion<br>b. Cough<br>c. Head ache<br>d. Fever<br>e. Vomiting<br>f. Diarrhea<br>g. Skin wounds<br>h. HIV<br>i. Zika<br>j. Dengue<br>k. Chikunguny a<br>l. TB<br>m. Otro<br>n. Don't know<br>o. Refused	a. Congestion<br>b. Cough<br>c. Head ache<br>d. Fever<br>e. Vomiting<br>f. Diarrhea<br>g. Skin wounds<br>h. HIV<br>i. Zika<br>j. Dengue<br>k. Chikunguny a<br>l. TB<br>m. Otro<br>n. Don't know<br>o. Refused	a. Congestion<br>b. Cough<br>c. Head ache<br>d. Fever<br>e. Vomiting<br>f. Diarrhea<br>g. Skin wounds<br>h. HIV<br>i. Zika<br>j. Dengue<br>k. Chikunguny a<br>l. TB<br>m. Otro<br>n. Don't know<br>o. Refused

Was this medicine prescribed to you (your child) at a health facility?<br>If YES, at which health facility was it recommended/ prescribed?<br>If NO, how did you (your child) know which medicine to buy?	Yes/No/ DK/Refused	Yes/No/ DK/Refused	Yes/No/ DK/Refused
	Centro de salud MSPAS/Puesto de salud/Clínica privada	Centro de salud MSPAS/Puesto de salud/Clínica privada	Centro de salud MSPAS/Puesto de salud/Clínica privada
	Amgiofamiliar/experiencia previa/ persona en la farmacia/médico/otro	Amgiofamiliar/experiencia previa/ persona en la farmacia/médico/otro	Amgiofamiliar/experiencia previa/ persona en la farmacia/médico/otro
Where did you (your child) get this medicine?	Pharmacy/Corner store(tienda)/Supermarket/MSPAS clinic/Friend or family member	Pharmacy/Corner store(tienda)/Supermarket/MSPAS clinic/Friend or family member	Pharmacy/Corner store(tienda)/Supermarket/MSPAS clinic/Friend or family member
Did you (your child) buy/receive a complete dose of the medicine?	Complete/partial dose/don´t know/refused	Complete/partial dose/don´t know/refused	Complete/partial dose/don´t know/refused
For how many days did you (your child) take this medication?	Number of days	Number of days	Number of days
How frequently do you (your child) take antibiotics?	Every month/Every 3 months/Every 6 months/every year/anytime it is necessary/Dont know/Dont respond	Every month/Every 3 months/Every 6 months/every year/anytime it is necessary/Dont know/Dont respond	Every month/Every 3 months/Every 6 months/every year/anytime it is necessary/Dont know/Dont respond
Did any other person also use this medication?	Yes/No	Yes/no	Yes/No
• If yes, who else used the medication	Specify	Specify	Specify

III. Exposure to hospitals and health centers

17. Do you work in the hospital?
    a. Yes (continue with following questions, clarifying that we are asking about visits to the hospital outside of work)
    b. No

18. In the past three months have you (your child) visited the hospital for any reason?
    a. Yes (go to Q19)
    b. No (go to Q20)
    c. Refused to answer (go to Q20)
    d. Don’t know (go to Q20)

19. Why did you (your child) visit the hospital?
    a. Reason unrelated to an illness
    b. Visit a patient who was hospitalized
    c. Healthy visit check up
    d. Outpatient treatment for illness
    e. Acute illness/hospitalization
    f. Other, specify____
    g. Don´t know

h. Refused to answer

20. In the last three months have you (your child) visited a health center for any reason?
    a. Yes (go to Q21)
    b. No (end)
    c. Refused to answer (end)
    d. Don't know (end)

21. Why did you (your child) go to the health center?
    a. Reason unrelated to an illness
    b. Visit a patient who was hospitalized
    c. Healthy visit check up
    d. Outpatient treatment for illness
    e. Acute illness/hospitalization
    f. Other, specify____
    g. Don´t know
    h. Refused to answer

---END
