# Supplementary material for: Antibiotic use and hygiene interact to influence the distribution of antimicrobial-resistant bacteria in low-income communities in Guatemala
Source: Sci Rep. 2020 Aug 13;10:13767. doi: 10.1038/s41598-020-70741-4 (PMC7426860; doi:10.1038/s41598-020-70741-4)
Supplement: Supplementary file 2 [file 41598_2020_70741_MOESM2_ESM.pdf]

Antibiotic use and hygiene interact to influence the distribution of antimicrobial-resistant bacteria in low-income communities in Guatemala

Brooke M. Ramay[¥1,2], Mark, A. Caudell[¥2,3,], Celia Cordon-Rosales*[1], L. Diego Archila[1], Guy H. Palmer[2,4], Claudia Jarquin[1], Purificación Moreno[1], John P. McCracken[1], Leah Rozenkrantz[5], Ofer Amram[2,6], Sylvia Omulo[2], Douglas R. Call[2,4]

Brooke M. Ramay, Pharm D[¥]

1) Center for Health Studies, Universidad del Valle de Guatemala,

181 Avenida 11-95 Guatemala City, Guatemala, 01015

2) Paul G. Allen School for Global Animal Health, Washington State University

Pullman, WA 99164, United States

Mark, A. Caudell, PhD[¥]

2) Paul G. Allen School for Global Animal Health, Washington State University

Pullman, WA 99164, United States

3)Food and Agriculture Organization of the United Nations, United Nations Complex

UN Avenue, Gigiri, Nairobi. PO Box: 30470, 00100

Celia Cordon-Rosales, B.S.*

1) Center for Health Studies, Universidad del Valle de Guatemala,

18 Avenida 11-95 Guatemala City, Guatemala, 01015

L. Diego Archila, PhD

1) Center for Health Studies, Universidad del Valle de Guatemala,

18 Avenida 11-95 Guatemala City, Guatemala, 01015

Guy H. Palmer PhD
2) Paul G. Allen School for Global Animal Health, Washington State University
Pullman, WA 99164, United States

4) Nelson Mandela African Institution of Science and Technology, P.O.BOX 447 Arusha, Tanzania

Claudia Jarquin MSc
1) Center for Health Studies, Universidad del Valle de Guatemala,
18 Avenida 11-95 Guatemala City, Guatemala, 01015

Purificación Moreno MSc
1) Center for Health Studies, Universidad del Valle de Guatemala,
18 Avenida 11-95 Guatemala City, Guatemala, 01015

John P. McCracken DrPH
1) Center for Health Studies, Universidad del Valle de Guatemala,
18 Avenida 11-95 Guatemala City, Guatemala, 01015

Leah Rozenkrantz PhD
5) Department of Geography, Simon Fraser University, Burnaby V5A 1S6, British Columbia, Canada

Ofer AmramPhD
2) Paul G. Allen School for Global Animal Health, Washington State University
Pullman, WA 99164, United States

6) Elson S. Floyd College of Medicine, Washington State University, Spokane, PO Box 1495, WA, USA

Sylvia Omulo, PhD

2) Paul G. Allen School for Global Animal Health, Washington State University
Pullman, WA 99164, United States

Douglas R. Call PhD
2) Paul G. Allen School for Global Animal Health, Washington State University
Pullman, WA 99164, United States

4) Nelson Mandela African Institution of Science and Technology, P.O.BOX 447 Arusha, Tanzania

¥ Authors contributed equally
*corresponding author: ccordon@ces.uvg.edu.gt, (502) 2329-8452

Cuestionario De Hogar

Record ID ______________________________

Fecha en que se realiza la encuesta ______________________________

I. Buenos dÃ-as/tardes, mi nombre es (NOMBRE PERSONA HABLANDO) y vengo hoy con mi compaÃ±ero de trabajo (NOMBRE COMPAÃ‘ERO DEL TRABAJO). Estamos visitando su casa el dÃ-a de hoy porque estamos realizando un estudio sobre la resistencia a los antibiÃ³ticos. Seria posible hablar con el jefe o encargado del hogar para ver si le interesa aprender mas sobre el estudio?

- ○ a. Si- continuar
- ○ b. No, no esta el jefe del hogar o encargado del hogar
- ○ c. No, no quiero escuchar mas

EXPLIQUE SOBRE EL ESTUDIO, Y QUE NECESITARaN LA PARTICIPACIoN DE UN ADULTO Y UN NIÃ‘O QUE SELECCIONE LA TABLET. PRIMERO SE SOLICITARa EL CONSENTIMIENTO DEL JEFE/ENCARGADO DE HOGAR.

El jefe o encargado de hogar ha dado su consentimiento para participar?

- ○ a. Si-Continuar
- ○ b. No-Terminar

1. Cuantas personas adultas (>18 aÃ±os) viven en esta vivienda ______________________________

2. Para cada persona adulta identificada en la pregunta 1; indique el nombre de cada uno.

indique el nombre persona 1 ______________________________

El adulto (1) ha vivido en la casa durante por lo menos un aÃ±o?

- ○ a. SI
- ○ b. No

El adulto (1) vivira en la misma casa por lo menos un aÃ±o mas?

- ○ a. Si
- ○ b. No

(1) ES ELEGIBLE O NO= ______________________________

PERSONA ELEGIBLE

indique el nombre persona 2 ______________________________

El adulto (2) ha vivido en la casa durante por lo menos un aÃ±o?

- ○ a. SI
- ○ b. No

El adulto (2) vivira en la misma casa por lo menos un aÃ±o mas?

- ○ a. Si
- ○ b. No

(2) ES ELEGIBLE O NO= ______________________________

PERSONA ELEGIBLE

indique el nombre persona 3 ______________________________

El adulto (3) ha vivido en la casa durante por lo menos un aÃ±o?

- ○ a. SI
- ○ b. No

El adulto (3) vivira en la misma casa por lo menos un aÃ±o mas?

- ○ a. Si
- ○ b. No

(3) ES ELEGIBLE O NO= ______________________________

PERSONA ELEGIBLE

indique el nombre persona 4 __________________________________

El adulto (4) ha vivido en la casa durante por lo menos un aÃ±o?
- ◯ a. SI
- ◯ b. No

El adulto (4) vivira en la misma casa por lo menos un aÃ±o mas?
- ◯ a. Si
- ◯ b. No

(4)ES ELEGIBLE O NO= __________________________________

PERSONA ELEGIBLE

indique el nombre persona 5 __________________________________

El adulto (5) ha vivido en la casa durante por lo menos un aÃ±o?
- ◯ a. SI
- ◯ b. No

El adulto (5) vivira en la misma casa por lo menos un aÃ±o mas?
- ◯ a. Si
- ◯ b. No

(5) ES ELEGIBLE O NO= __________________________________

PERSONA ELEGIBLE

indique el nombre persona 6 __________________________________

El adulto (6) ha vivido en la casa durante por lo menos un aÃ±o?
- ◯ a. SI
- ◯ b. No

El adulto (6) vivira en la misma casa por lo menos un aÃ±o mas?
- ◯ a. Si
- ◯ b. No

(6)ES ELEGIBLE O NO= __________________________________

PERSONA ELEGIBLE

indique el nombre persona 7 __________________________________

El adulto (7) ha vivido en la casa durante por lo menos un aÃ±o?
- ◯ a. SI
- ◯ b. No

El adulto (7) vivira en la misma casa por lo menos un aÃ±o mas?
- ◯ a. Si
- ◯ b. No

(7) ES ELEGIBLE O NO= __________________________________

PERSONA ELEGIBLE

indique el nombre persona 8 __________________________________

El adulto (8) ha vivido en la casa durante por lo menos un aÃ±o?
- ◯ a. SI
- ◯ b. No

El adulto (8) vivira en la misma casa por lo menos un aÃ±o mas?
- ◯ a. Si
- ◯ b. No

(8)ES ELEGIBLE O NO= __________________________________

PERSONA ELEGIBLE

indique el nombre persona 9 __________________________________

El adulto (9) ha vivido en la casa durante por lo menos un aÃ±o?
- a. SI
- b. No

El adulto (9) vivira en la misma casa por lo menos un aÃ±o mas?
- a. Si
- b. No

(9)ES ELEGIBLE O NO= ______________________

PERSONA ELEGIBLE

indique el nombre persona 10 ______________________

El adulto (10) ha vivido en la casa durante por lo menos un aÃ±o?
- a. SI
- b. No

El adulto (10) vivira en la misma casa por lo menos un aÃ±o mas?
- a. Si
- b. No

(10)ES ELEGIBLE O NO= ______________________

PERSONA ELEGIBLE

Lance el dado y escribe el numero que sale (1 al 12)... Seleccione a la persona ADULTA con base en el numero ______________________

3. Cuantos niÃ±os de 0 a 4 aÃ±os viven en esta vivienda? ______________________

4; Para cada niÃ±o de 0 a 4 aÃ±os en la pregunta 3; indique el nombre de cada uno.

indique el nombre del niÃ±o 1 ______________________

El niÃ±o (1) ha vivido en la casa durante por lo menos un aÃ±o?
- a. Si
- b. No

El niÃ±o (1) vivira en la misma casa por lo menos un aÃ±o mas?
- a. Si
- b. No

(1) ES ELEGIBLE O NO= ______________________

NIÃ‘O ELEGIBLE

indique el nombre del niÃ±o 2 ______________________

El niÃ±o (2) ha vivido en la casa durante por lo menos un aÃ±o?
- a. Si
- b. No

El niÃ±o (2) vivira en la misma casa por lo menos un aÃ±o mas?
- a. Si
- b. No

(2) ES ELEGIBLE O NO= ______________________

NIÃ‘O ELEGIBLE

indique el nombre del niÃ±o 3 ______________________

El niÃ±o (3) ha vivido en la casa durante por lo menos un aÃ±o?
- a. Si
- b. No

El niÃ±o (3) vivira en la misma casa por lo menos un aÃ±o mas?
- a. Si
- b. No

(3) ES ELEGIBLE O NO= ______________________

NIÃ‘O ELEGIBLE

indique el nombre del niÃ±o 4 ______________________

El niÃ±o (4) ha vivido en la casa durante por lo menos un aÃ±o?
◯ a. Si
◯ b. No

El niÃ±o (4) vivira en la misma casa por lo menos un aÃ±o mas?
◯ a. Si
◯ b. No

(4) ES ELEGIBLE O NO= ______________________

NIÃ‘O ELEGIBLE

indique el nombre del niÃ±o 5 ______________________

El niÃ±o (5) ha vivido en la casa durante por lo menos un aÃ±o?
◯ a. Si
◯ b. No

El niÃ±o (5) vivira en la misma casa por lo menos un aÃ±o mas?
◯ a. Si
◯ b. No

(5) ES ELEGIBLE O NO= ______________________

NIÃ‘O ELEGIBLE

indique el nombre del niÃ±o 6 ______________________

El niÃ±o (6) ha vivido en la casa durante por lo menos un aÃ±o?
◯ a. Si
◯ b. No

El niÃ±o (6) vivira en la misma casa por lo menos un aÃ±o mas?
◯ a. Si
◯ b. No

(6) ES ELEGIBLE O NO= ______________________

NIÃ‘O ELEGIBLE

indique el nombre del niÃ±o 7 ______________________

El niÃ±o (7) ha vivido en la casa durante por lo menos un aÃ±o?
◯ a. Si
◯ b. No

El niÃ±o (7) vivira en la misma casa por lo menos un aÃ±o mas?
◯ a. Si
◯ b. No

(7) ES ELEGIBLE O NO= ______________________

NIÃ‘O ELEGIBLE

indique el nombre del niÃ±o 8 ______________________

El niÃ±o (8) ha vivido en la casa durante por lo menos un aÃ±o?
◯ a. Si
◯ b. No

El niÃ±o (8) vivira en la misma casa por lo menos un aÃ±o mas?
◯ a. Si
◯ b. No

(8) ES ELEGIBLE O NO= ______________________

NIÃ‘O ELEGIBLE

indique el nombre del niÃ±o 9 ______________________

El niÃ±o (9) ha vivido en la casa durante por lo menos un aÃ±o?
- ◯ a. Si
- ◯ b. No

El niÃ±o (9) vivira en la misma casa por lo menos un aÃ±o mas?
- ◯ a. Si
- ◯ b. No

(9) ES ELEGIBLE O NO= ______________________

NIÃ‘O ELEGIBLE

indique el nombre del niÃ±o 10 ______________________

El niÃ±o (10) ha vivido en la casa durante por lo menos un aÃ±o?
- ◯ a. Si
- ◯ b. No

El niÃ±o (10) vivira en la misma casa por lo menos un aÃ±o mas?
- ◯ a. Si
- ◯ b. No

(10) ES ELEGIBLE O NO= ______________________

NIÃ‘O ELEGIBLE

Lance el dado y escribe el numero que sale (1 al 12)... Seleccione al NIÃ‘O con base en el numero ______________________

(Explicar que la Tablet escogera a un adulto y un niÃ±o para hacerles el cuestionario. Al seleccionar a los participantes, explicarles el estudio y pedirles el consentimiento. Si las personas seleccionadas no estan alli en ese momento, pregunte a que hora podra encontrarlas y explique que volvera).

5. Se obtuvo el consentimiento informado para el adulto?
- ◯ a. Si
- ◯ b. No se encuentra en casa el adulto seleccionado (Apuntar datos de contacto)
- ◯ c. No hay ningÃºn adulto que quiera participar

6. Se obtuvo el consentimiento informado para el niÃ±o?
- ◯ a. Si
- ◯ b. No se encuentra en casa el niÃ±o seleccionado (Apuntar datos de contacto)
- ◯ c. No hay ningÃºn niÃ±o que quiera participar

INFORMACIoN DEMOGRaFICA del representante del hogar

1. ID del representante del hogar ______________________

2. Localidad
- ◯ Coatepeque
- ◯ San Juan Ostuncalco

a. Coatepeque (listado definido de opciones)
- ◯ 1.El Jardin
- ◯ 2.La Union

b. San Juan Ostuncalco (listado definido de opciones)
- ◯ 1.Casco Urbano
- ◯ 2.Monrovia

3. Sexo
- ◯ a. Masculino
- ◯ b. Femenino

4. Cuantos aÃ±os tiene? (anotar # aÃ±os completos) ______________________

5. Usted es el jefe del hogar?
- ◯ a. Si
- ◯ b. No

6. Especificar, cual es su relacion con el jefe del hogar

- ◯ a. Mama/Papa
- ◯ b. Esposa/esposo
- ◯ c. Hermana/Hermano
- ◯ d. CuÃ±ada/CuÃ±ado
- ◯ e. Hija/Hijo
- ◯ f. Nieta/Nieto

7. De que etnia se considera usted?

- ◯ a. IndÃ-gena
- ◯ b. Ladino
- ◯ c. Otro. Especificar.
- ◯ d. Desconocido
- ◯ e. Se rehÃºsa a responder

Otro ________________________________

8. Si se identifica como indigena, A cual grupo etnico pertenece usted?

- ◯ a. Maya
- ◯ b. Xinca
- ◯ c. Ladino
- ◯ d. GarÃ-funa
- ◯ e. Mestizo
- ◯ f. Otro
- ◯ g. Se rehÃºsa a responder

Otro ________________________________

9. Que idioma hablan por lo general en su hogar?

- ◯ a. EspaÃ±ol
- ◯ b. K'iche'
- ◯ c. Mam
- ◯ d. kackchiquel
- ◯ e. Otro
- ◯ f. Se rehÃºsa a responder

Otro ________________________________

10. Sabe usted leer y escribir?

- ◯ a. Si
- ◯ b. No
- ◯ c. Se rehÃºsa a responder

11. Cual fue el nivel academico que usted completo?

- ◯ a. Ninguna educaciÃ³n formal (no fue al colegio)
- ◯ b. No completÃ³ educaciÃ³n primaria
- ◯ c. CompletÃ³ educaciÃ³n primaria
- ◯ d. CompletÃ³ educaciÃ³n secundaria
- ◯ e. CompletÃ³ educaciÃ³n mayor a la secundaria
- ◯ f. Otro
- ◯ g. No quiere responder

Otro: ________________________________

Aparatos del hogar

Celular?

- ◯ Si
- ◯ No
- ◯ No sabe
- ◯ Se rehÃºsa a responder

TV?

- ◯ Si
- ◯ No
- ◯ No sabe
- ◯ Se rehÃºsa a responder

Transporte

Carro o camion?
- ◯ a. Si
- ◯ b. No
- ◯ c. Se rehÃºsa a responder

Electrodomesticos

Radio?
- ◯ a. Si
- ◯ b. No
- ◯ c. Se rehÃºsa a responder

Refrigerador?
- ◯ a. Si
- ◯ b. No
- ◯ c. Se rehÃºsa a responder

Lavadora?
- ◯ a. Si
- ◯ b. No
- ◯ c. Se rehÃºsa a responder

Secadora?
- ◯ a. Si
- ◯ b. No
- ◯ c. Se rehÃºsa a responder

Microondas?
- ◯ a. Si
- ◯ b. No
- ◯ c. Se rehÃºsa a responder

Comunicacion

Telefono fijo?
- ◯ a. Si
- ◯ b. No
- ◯ c. Se rehÃºsa a responder

13. Que ingresos mensuales recibe la familia?
- ◯ a. Menor a Q1,000.00
- ◯ b. Q1,001.00 a Q3,000.00
- ◯ c. Q3,001.00 a Q5,000.00
- ◯ d. >Q5,000.00
- ◯ e. No sabe
- ◯ f. Se rehÃºsa a responder

ENCUESTADOR LEA: Ahora le hare unas preguntas sobre las fuentes de agua en su casa, materiales de construccion, y el manejo de los desechos y basura. Me gustaria comenzar haciendole unas preguntas sobre la fuente de agua potable disponible en su hogar.

15. Durante la temporada de lluvia, cual es la fuente principal de agua para tomar que usan los miembros de su hogar?

- ◯ a. Agua embotellada o agua en bolsa (agua pura)
- ◯ b. Agua de camiÃ³n cisterna
- ◯ c. Agua de lluvia recolectada - privada
- ◯ d. Agua de lluvia recolectada - pÃºblica
- ◯ e. Agua superficial (rÃ-o, presa, lago, estanque, arroyo, canal, canales de riego)
- ◯ f. Agua entubada dentro de casa (chorro privado)
- ◯ g. Agua entubada que entra al patio/jardÃ-n/lote (chorro privado)
- ◯ h. Agua entubada pÃºblica (chorro pÃºblico)
- ◯ i. Agua de pozo perforado privado
- ◯ j. Agua de pozo perforado pÃºblico
- ◯ k. Pozo excavado protegido - privado
- ◯ l. Pozo excavado protegido - pÃºblico
- ◯ m. Pozo excavado no-protegido - privado
- ◯ n. Pozo excavado no-protegido - pÃºblico
- ◯ o. Nacimiento de agua protegido - privado
- ◯ p. Nacimiento de agua protegido - pÃºblico
- ◯ q. Nacimiento de agua no-protegido - privado
- ◯ r. Nacimiento de agua no-protegido - pÃºblico
- ◯ s. Agua de tanque pequeÃ±o
- ◯ t. Otra (especificar)
- ◯ u. No sabe

Otro ________________________________

17. En la temporada seca, cual es la fuente principal de agua para tomar los miembros de su hogar? (MARQUE UNO)

- ◯ a. Agua embotellada o agua en bolsa (agua pura)
- ◯ b. Agua de camiÃ³n cisterna
- ◯ c. Agua de lluvia recolectada - pÃºblica
- ◯ d. Agua acarreada de tanque pequeÃ±o
- ◯ e. Agua superficial (rÃ-o, presa, lago, estanque, arroyo, canal, canales de riego)
- ◯ f. Agua entubada dentro de casa (chorro privado)
- ◯ g. Agua entubada que entra al patio/jardÃ-n/lote (chorro privado)
- ◯ h. Agua entubada pÃºblica (chorro pÃºblico)
- ◯ i. Agua de pozo perforado privado
- ◯ j. Agua de pozo perforado pÃºblico
- ◯ k. Pozo excavado protegido - privado
- ◯ l. Pozo excavado protegido - pÃºblico
- ◯ m. Pozo excavado no-protegido - privado
- ◯ n. Pozo excavado no-protegido - pÃºblico
- ◯ o. Nacimiento de agua protegido - privado
- ◯ p. Nacimiento de agua protegido - pÃºblico
- ◯ q. Nacimiento de agua no-protegido - privado
- ◯ r. Nacimiento de agua no-protegido - pÃºblico
- ◯ s. Agua de lluvia recolectada - privada
- ◯ t. Otra (especificar)
- ◯ u. No sabe

Otro ________________________________

18. Almacena usted, el agua para tomar, en recipientes especiales solo para agua?

- ◯ a. Si
- ◯ b. No
- ◯ c. No sabe
- ◯ d. Se rehÃºsa a responder

19. Como almacena los recipientes cuando estan llenos con agua?

- ◯ a. Tapados
- ◯ b. Destapados

20. En donde se almacena los recipientes cuando estan llenos con agua?

- ◯ a. Dentro de la casa
- ◯ b. Afuera de la casa

21. Como almacena los recipientes cuando estan vacios?
- ◯ a. Tapado
- ◯ b. Destapado

22. En donde se almacena los recipientes cuando estan vacios?
- ◯ a. Dentro de la casa
- ◯ b. Afuera de la casa

23. Lava el recipiente antes de usarlo?
- ◯ a. SÃ-
- ◯ b. No
- ◯ 3. No sabe
- ◯ 4. Se rehÃºsa a responder

24. Como lava el recipiente? (marque los que aplican)
- ☐ a. Con agua y jabÃ³n
- ☐ b. Ãšnicamente con desinfectante (ej.: cloro)
- ☐ c. Con agua, jabÃ³n y desinfectante
- ☐ d. Hervir el recipiente
- ☐ e. No sabe
- ☐ f. Se rehÃºsa a responder

25. Le hace usted algun tratamiento al agua antes de tomarla?
- ◯ a. SÃ-
- ◯ b. No
- ◯ c. No sabe
- ◯ d. Se rehÃºsa a responder

26. Que le hace al agua? (Solamente marque mas de una respuesta si se usan varios metodos a la vez, por ejemplo, filtracion y cloracion)
- ☐ a. Hervir
- ☐ b. Agrega cloro
- ☐ c. Agrega yodo
- ☐ d. La cuela a travÃ©s de una tela
- ☐ e. Usa un filtro de agua (cerÃ¡mico, arena, compuesto, etc.)
- ☐ f. DesinfecciÃ³n solar / SODIS
- ☐ g. Deja el agua reposar
- ☐ h. Agrega lana/floculante
- ☐ i. Otro (especificar)
- ☐ j. No sabe
- ☐ k. Se rehÃºsa a responder

Otro ______________________________

27. Cuando fue la ultima vez que usted trato el agua para tomar usando este metodo? (MARQUE UNO)
- ◯ a. Hoy
- ◯ b. Ayer
- ◯ c. Hace mÃ¡s de un dÃ-a, pero menos de una semana
- ◯ d. De una a cuatro semanas
- ◯ e. Hace mÃ¡s de un mes
- ◯ f. Otra(especificar)
- ◯ g. No sabe/ no recuerda
- ◯ h. Se rehÃºsa a responder

Otro ______________________________

28. Con que frecuencia le da usted tratamiento al agua para tomar usando este metodo? (MARQUE UNO)
- ◯ a. Siempre (para toda el agua que se toma en la casa)
- ◯ b. Algunas veces
- ◯ d. No sabe/ no recuerda
- ◯ e. Se rehÃºsa a responder

29. Usa usted el agua del rio en su casa?
- ◯ a. Si
- ◯ b. No
- ◯ c. No sabe
- ◯ d. Se rehÃºsa a responder
- ◯ e. No aplica, no hay rÃ-o cercano

30. Para que se usa el agua del rio en su casa? (seleccionar todos los que aplican)

- ☐ a. Cocinar
- ☐ b. Lavar utensilios de cocina
- ☐ c. Lavar ropa
- ☐ d. BaÃ±arse
- ☐ e. Regar jardines
- ☐ f. No sabe
- ☐ g. Se rehÃºsa a responder

31. Que tipo de sanitario tiene? (indicar cual usan con mas frecuencia los miembros de su familia) (MARQUE UNO)

- ◯ a. Inodoro con jalador de agua/donde se echa agua
- ◯ b. Letrina de pozo/Hoyo de letrina con losa
- ◯ c. Letrina de pozo sin losa/abierta/Pozo ciego
- ◯ d. No tienen facilidades (defecaciÃ³n al aire libre)
- ◯ e. Compostaje / inodoro seco o letrina abonera
- ◯ f. Servicio o cubeta (donde las heces se eliminan manualmente)
- ◯ g. Letrina de hoyo mejorada y ventilada (VIP
- ◯ h. Un sanitario compartido
- ◯ i. Inodoro suspendido / letrina colgante
- ◯ j. Otra
- ◯ k. No sabe
- ◯ l. Se rehÃºsa a responder

Otro ________________________________

32. Cuando jala o echa agua en el inodoro o letrina A donde van los desechos? (MARQUE UNO)

- ◯ a. Drenaje en el sistema de desagÃ¼e con tuberÃ-a
- ◯ b. Drenaje en tanque de la fosa sÃ©ptica
- ◯ c. Va al pozo de la letrina
- ◯ d. No hay drenaje (a flor de tierra)
- ◯ e. Drenaje en cualquier otro lado
- ◯ f. Drenaje en un sitio desconocido/no estÃ¡ seguro/no sabe en donde
- ◯ g. No sabe
- ◯ h. Otro

Otro ________________________________

33. En donde esta ubicado el inodoro/letrina? (MARQUE UNO)

- ◯ a. Adentro o a la par de la casa
- ◯ b. Afuera del lote
- ◯ c. No sabe
- ◯ d. Se rehÃºsa a responder
- ◯ e. Otro

Otro ________________________________

34. Comparten el inodoro/letrina con otros hogares?

- ◯ a. Si
- ◯ b. No
- ◯ c. No sabe
- ◯ d. Se rehÃºsa a responder

35. Su inodoro es de uso comunitario (lo alquila)?

- ◯ a. SÃ-
- ◯ b. No
- ◯ c. No sabe
- ◯ d. Se rehÃºsa a responder

36. Limpia (en alguna ocasion) el inodoro/letrina?

- ◯ a. SÃ-
- ◯ b. No
- ◯ c. No sabe
- ◯ d. Se rehÃºsa a responder

37. Cuando fue la ultima vez que limpio el inodoro?

- ◯ a. Hoy
- ◯ b. Ayer
- ◯ c. Hace mÃis de un dÃ-a, pero menos de una semana
- ◯ d. Hace 1 a 4 semanas
- ◯ e. Hace mÃis de un mes
- ◯ f. No sabe
- ◯ g. Se rehÃºsa a responder

38. La ultima vez que el niÃ±o mas pequeÃ±o de este hogar defeco, como desecharon las heces? (MARQUE UNO)

- ◯ a. El niÃ±o usÃ³ el inodoro/letrina
- ◯ b. Se pusieron adentro del inodoro o letrina
- ◯ c. Se pusieron adentro/enjuagaron en el desagÃ¼e o zanja (o lo lavaron en la pila)
- ◯ d. Se tiraron a la basura
- ◯ e. Se enterraron
- ◯ f. Se dejaron en el patio al aire libre
- ◯ g. Se dejaron afuera del lote al aire libre
- ◯ h. Otro
- ◯ i. No sabe
- ◯ j. Se rehÃºsa a responder

Otro ________________________________

39. Observar. De que material esta principalmente construido el piso de la casa?

- ◯ a. Piso natural- tierra/arena
- ◯ b. Piso terminado- cerÃimico
- ◯ c. Piso terminado- cemento o concreto
- ◯ d. Piso natural- paja/aserrÃ-n
- ◯ e. Piso rudimentario- tablas de madera
- ◯ f. Piso rudimentario- palma/bambÃº
- ◯ g. Piso terminado- parquÃ© o madera pulida
- ◯ h. Piso terminado- alfombra
- ◯ i. Otro, especificar

Otro ________________________________

40. Observar: Hay excremento de algun animal en el piso de la casa?

- ◯ a. SÃ-
- ◯ b. No
- ◯ c. No es posible determinar

41. Observar. Cual es el material principal del techo de la casa?

- ◯ a. Techo terminado- metal de lÃimina
- ◯ b. Techo terminado- cemento/concreto
- ◯ c. Techo terminado- teja
- ◯ d. No hay techo
- ◯ e. Techo natural- paja/hoja de palma
- ◯ f. Techo natural- cÃ©sped
- ◯ g. Techo rudimentario- tapete rÃºstico Techo rudimentario- palma/bambÃº
- ◯ h. Techo rudimentario- tablas de madera
- ◯ i. Techo rudimentario- cartÃ³n/cartÃ³n piedra
- ◯ j. Techo terminado- madera
- ◯ k. Techo terminado- calamina/fibra de cemento
- ◯ l. Techo terminado- ladrillos de cerÃimica
- ◯ m. Techo terminado- plÃistico
- ◯ n. Otro. especificar

Otro ________________________________

42. Observar. Cual es el material principal de las paredes de la casa?

- ◯ a. Paredes terminadas - bloques de cemento
- ◯ b. Paredes terminadas - adobe cubierto
- ◯ c. Paredes rudimentarios- adobe descubierto
- ◯ d. Sin paredes
- ◯ e. Paredes naturales- CAÃ‘A/HOJA DE PALMA/TRONCOS
- ◯ f. Paredes naturales- Lodo
- ◯ g. Paredes rudimentarios- ladrillos de lodo
- ◯ h. Paredes rudimentarios- BambÃº con lodo
- ◯ i. Paredes rudimentarios- piedra con lodo
- ◯ j. Paredes rudimentarios- plywood
- ◯ k. Paredes rudimentarios- cartÃ³n/cartÃ³n piedra
- ◯ l. Paredes rudimentarios- madera reusada
- ◯ m. Paredes terminadas- cemento o concreto
- ◯ n. Paredes terminadas - piedra con cal
- ◯ o. Paredes terminadas - ladrillos
- ◯ p. Paredes terminadas - tablas de madera
- ◯ q. Otro, especificar

Otro ______________________________

43. Cuantas casas (contadas por cocinas) hay atras de la puerta principal? ______________________________

44. Cuantos cuartos para dormir tiene la casa del representante? ______________________________

45. La cocina esta fisicamente separada de los cuartos utilizados para dormir?

- ◯ a. Si
- ◯ b. No

46. Que hace con la basura en su hogar? (puede escoger mas de uno)

- ☐ a. La quema
- ☐ b. Se utiliza como "combustible" para cocinar
- ☐ c. La recicla
- ☐ d. La entierra
- ☐ e. La colectan para llevarla al basurero general
- ☐ f. La colectan para llevarla al relleno
- ☐ g. La colectan para llevarla al barranco
- ☐ h. No sabe
- ☐ i. Otro
- ☐ j. Se rehÃºsa a responder

Otro ______________________________

47. Usa usted una estufa de leÃ±a, gas o ambos para cocinar?

- ◯ a. LeÃ±a
- ◯ b. Gas
- ◯ c. Ambos
- ◯ d. Ninguno
- ◯ e. No sabe
- ◯ f. Se rehÃºsa a responder

48. Si usa una estufa de leÃ±a, que tipo de estufa es la que usa por lo general para cocinar? (MARQUE UNO)

- ◯ a. Estufa de chimenea (la plancha)
- ◯ b. Estufa de fuego abierto
- ◯ c. Otra(especificar)
- ◯ d. No sabe
- ◯ e. Se rehÃºsa a responder

Otro ______________________________

49. Lavan los utensilios de cocina antes de usarlos?

- ◯ a. SÃ-
- ◯ b. No
- ◯ c. No sabe
- ◯ d. Se rehÃºsa a responder

Otro ________________________________

50. Como lava los utensilios? (marque los que aplican)

- ☐ a. Con agua y jabÃ³n
- ☐ b. Ãšnicamente con desinfectante (ej.: cloro)
- ☐ c. Con agua, jabÃ³n y desinfectante
- ☐ d. Hervir los utensilios
- ☐ e. Otro
- ☐ f. No sabe
- ☐ g. Se rehÃºsa a responder

51. Cual es la edad aproximada de la persona que normalmente lava los trastos?

- ◯ a. 0-5 aÃ±os
- ◯ b. 6-12 aÃ±os
- ◯ c. 13-18 aÃ±os
- ◯ d. 19 aÃ±os en adelante
- ◯ e. No sabe
- ◯ f. Se rehÃºsa a responder

ENCUESTADOR LEA: Ahora me gustarÃa preguntarle sobre los hÃ¡bitos de higiene en el hogar. Estas preguntas tienen que ver principalmente con el lavado de manos en su hogar.

52. Por favor mencione todos los sitios en donde los miembros de su familia pudieron lavarse las manos en las ultimas dos semanas (MARQUE TODOS LOS QUE APLIQUEN)

- ☐ a. Sitio en o cerca del inodoro adentro de la casa
- ☐ b. Sitio en o cerca de la cocina dentro del lote o terreno
- ☐ c. Sitio cerca del corral de animales
- ☐ d. En cualquier lugar dentro del lote
- ☐ e. Afuera del lote
- ☐ f. Otro
- ☐ g. Nunca se lava las manos/no hay lugar donde hacerlo
- ☐ h. No sabe
- ☐ i. Se rehÃºsa a responder

Otro ________________________________

SecciÃ³n 3 ENCUESTADOR LEA: Muchas gracias por su tiempo. Ahora quiero hacerle unas preguntas sobre varias condiciones en su hogar. Estas preguntas las hacemos en cada casa que visitamos.

ANIMALES FUERA DE LA CASA

53. Los miembros de su familia tienen animales que viven libremente afuera de la casa? (MARQUE UNO)

- ◯ a. Si
- ◯ b. No
- ◯ d. Se rehÃºsa a responder

54. Que animales tienen? (MARQUE TODAS LAS QUE APLIQUEN, INDICAR CUANTOS ANIMALES DE CADA TIPO)

- ☐ a. Perros
- ☐ b. Gatos
- ☐ c. Pollos
- ☐ d. Pavos
- ☐ e. Patos
- ☐ f. Gansos
- ☐ g. Otros pÃ¡jaros
- ☐ h. Cerdos
- ☐ i. Vacas
- ☐ j. Ovejas
- ☐ k. Cabras
- ☐ l. Otros animales, especificar
- ☐ m. No sabe
- ☐ n. Se rehÃºsa a responder

Cual otro animal? ______________________________

PERROS

Cuantos Perros? ______________________________

Alguna vez les ha dado antibioticos a su/sus perro/perros?

- ◯ a. Si
- ◯ b. No
- ◯ c. No sabe
- ◯ d. Se rehÃºsa a responder

DATOS MEDICINA PERROS
Nombre de la Medicina/Antibiotico ______________________________

â€¢ Medicina a la vista y se puede leer el nombre

- ◯ Si
- ◯ No

â€¢ Caja de la medicina o botella vista (si/no)

- ◯ Si
- ◯ No

Alguna vez ha dejado de comer o tomar leche/carne/huevos del animal indicado, despuÃ©s de darle antibiÃ³ticos?

- ◯ SI
- ◯ No

Durante cuanto tiempo ha dejado de tomarla? (solo aplica si dice que si a la pregunta anterior) (Numero de dias) ______________________________

GATOS

Cuantos Gatos? ______________________________

Alguna vez les ha dado antibioticos a su/sus gato/gatos?

- ◯ a. Si
- ◯ b. No
- ◯ c. No sabe
- ◯ d. Se rehÃºsa a responder

DATOS MEDICINA GATOS
Nombre de la Medicina/Antibiotico ______________________________

â€¢ Medicina a la vista y se puede leer el nombre
◯ Si
◯ No

â€¢ Caja de la medicina o botella vista (si/no)
◯ Si
◯ No

Alguna vez ha dejado de comer o tomar leche/carne/huevos del animal indicado, despuÃ©s de darle antibiÃ³ticos?
◯ SI
◯ No

Durante cuanto tiempo ha dejado de tomarla? (solo aplica si dice que si a la pregunta anterior) (Numero de dias)
____

PAVOS

Cuantos Pavos?
____

Alguna vez les ha dado antibioticos a su/sus pavo/pavos?
◯ a. Si
◯ b. No
◯ c. No sabe
◯ d. Se rehÃºsa a responder

DATOS MEDICINA PAVOS
Nombre de la Medicina/Antibiotico
____

â€¢ Medicina a la vista y se puede leer el nombre
◯ Si
◯ No

â€¢ Caja de la medicina o botella vista (si/no)
◯ Si
◯ No

Alguna vez ha dejado de comer o tomar leche/carne/huevos del animal indicado, despuÃ©s de darle antibiÃ³ticos?
◯ SI
◯ No

Durante cuanto tiempo ha dejado de tomarla? (solo aplica si dice que si a la pregunta anterior) (Numero de dias)
____

POLLOS

Cuantos Pollos?
____

Alguna vez les ha dado antibioticos a su/sus pollo/pollos?
◯ a. Si
◯ b. No
◯ c. No sabe
◯ d. Se rehÃºsa a responder

DATOS MEDICINA POLLOS
Nombre de la Medicina/Antibiotico
____

â€¢ Medicina a la vista y se puede leer el nombre
◯ Si
◯ No

â€¢ Caja de la medicina o botella vista (si/no)
◯ Si
◯ No

Alguna vez ha dejado de comer o tomar leche/carne/huevos del animal indicado, despuÃ©s de darle antibiÃ³ticos?
◯ SI
◯ No

Durante cuanto tiempo ha dejado de tomarla? (solo aplica si dice que si a la pregunta anterior) (Numero de dias)
____

PATOS

Cuantos Patos?
____

Alguna vez les ha dado antibioticos a su/sus pato/patos?
◯ a. Si
◯ b. No
◯ c. No sabe
◯ d. Se rehÃºsa a responder

DATOS MEDICINA PATOS
Nombre de la Medicina/Antibiotico
____

â€¢ Medicina a la vista y se puede leer el nombre
◯ Si
◯ No

â€¢ Caja de la medicina o botella vista (si/no)
◯ Si
◯ No

Alguna vez ha dejado de comer o tomar leche/carne/huevos del animal indicado, despuÃ©s de darle antibiÃ³ticos?
◯ SI
◯ No

Durante cuanto tiempo ha dejado de tomarla? (solo aplica si dice que si a la pregunta anterior) (Numero de dias)
____

GANSOS

Cuantos Gansos?
____

Alguna vez les ha dado antibioticos a su/sus ganso/gansos?
◯ a. Si
◯ b. No
◯ c. No sabe
◯ d. Se rehÃºsa a responder

DATOS MEDICINA GANSOS
Nombre de la Medicina/Antibiotico
____

â€¢ Medicina a la vista y se puede leer el nombre
◯ Si
◯ No

â€¢ Caja de la medicina o botella vista (si/no) | ◯ Si ◯ No

Alguna vez ha dejado de comer o tomar leche/carne/huevos del animal indicado, despuÃ©s de darle antibiÃ³ticos? | ◯ SI ◯ No

Durante cuanto tiempo ha dejado de tomarla? (solo aplica si dice que si a la pregunta anterior) (Numero de dias) | ______________________

PAJAROS

Cuantos otros pajaros? | ______________________

Alguna vez les ha dado antibioticos a su/sus pajaro/pajaros?

◯ a. Si
◯ b. No
◯ c. No sabe
◯ d. Se rehÃºsa a responder

DATOS MEDICINA PAJAROS
Nombre de la Medicina/Antibiotico | ______________________

â€¢ Medicina a la vista y se puede leer el nombre | ◯ Si ◯ No

â€¢ Caja de la medicina o botella vista (si/no) | ◯ Si ◯ No

Alguna vez ha dejado de comer o tomar leche/carne/huevos del animal indicado, despuÃ©s de darle antibiÃ³ticos? | ◯ SI ◯ No

Durante cuanto tiempo ha dejado de tomarla? (solo aplica si dice que si a la pregunta anterior) (Numero de dias) | ______________________

CERDOS

Cuantos Cerdos? | ______________________

Alguna vez les ha dado antibioticos a su/sus cerdo/cerdos?

◯ a. Si
◯ b. No
◯ c. No sabe
◯ d. Se rehÃºsa a responder

DATOS MEDICINA CERDOS
Nombre de la Medicina/Antibiotico | ______________________

â€¢ Medicina a la vista y se puede leer el nombre | ◯ Si ◯ No

â€¢ Caja de la medicina o botella vista (si/no)
◯ Si
◯ No

Alguna vez ha dejado de comer o tomar leche/carne/huevos del animal indicado, despuÃ©s de darle antibiÃ³ticos?
◯ SI
◯ No

Durante cuanto tiempo ha dejado de tomarla? (solo aplica si dice que si a la pregunta anterior) (Numero de dias)
____

VACAS

Cuantos Vacas?
____

Alguna vez les ha dado antibioticos a su/sus vaca/vacas?
◯ a. Si
◯ b. No
◯ c. No sabe
◯ d. Se rehÃºsa a responder

DATOS MEDICINA VACAS
Nombre de la Medicina/Antibiotico
____

â€¢ Medicina a la vista y se puede leer el nombre
◯ Si
◯ No

â€¢ Caja de la medicina o botella vista (si/no)
◯ Si
◯ No

Alguna vez ha dejado de comer o tomar leche/carne/huevos del animal indicado, despuÃ©s de darle antibiÃ³ticos?
◯ SI
◯ No

Durante cuanto tiempo ha dejado de tomarla? (solo aplica si dice que si a la pregunta anterior) (Numero de dias)
____

CABRAS

Cuantos Cabras?
____

Alguna vez les ha dado antibioticos a su/sus cabra/cabras?
◯ a. Si
◯ b. No
◯ c. No sabe
◯ d. Se rehÃºsa a responder

DATOS MEDICINA CABRAS
Nombre de la Medicina/Antibiotico
____

â€¢ Medicina a la vista y se puede leer el nombre
◯ Si
◯ No

â€¢ Caja de la medicina o botella vista (si/no)
◯ Si
◯ No

Alguna vez ha dejado de comer o tomar leche/carne/huevos del animal indicado, despuÃ©s de darle antibiÃ³ticos?	◯ SI<br>◯ No
Durante cuanto tiempo ha dejado de tomarla? (solo aplica si dice que si a la pregunta anterior)<br>(Numero de dias)	__________________________________

OVEJAS

Cuantos Ovejas?	__________________________________
Alguna vez les ha dado antibioticos a su/sus oveja/ovejas?	◯ a. Si<br>◯ b. No<br>◯ c. No sabe<br>◯ d. Se rehÃºsa a responder
DATOS MEDICINA OVEJAS<br>Nombre de la Medicina/Antibiotico	__________________________________
â€¢ Medicina a la vista y se puede leer el nombre	◯ Si<br>◯ No
â€¢ Caja de la medicina o botella vista (si/no)	◯ Si<br>◯ No
Alguna vez ha dejado de comer o tomar leche/carne/huevos del animal indicado, despuÃ©s de darle antibiÃ³ticos?	◯ SI<br>◯ No
Durante cuanto tiempo ha dejado de tomarla? (solo aplica si dice que si a la pregunta anterior)<br>(Numero de dias)	__________________________________
a. Otros animales, especificar la cantidad	__________________________________

OTROS ANIMALES

Alguna vez les ha dado antibioticos a sus otros animales?	◯ a. Si<br>◯ b. No<br>◯ c. No sabe<br>◯ d. Se rehÃºsa a responder
DATOS MEDICINA OTROS ANIMALES<br>Nombre de la Medicina/Antibiotico	__________________________________
â€¢ Medicina a la vista y se puede leer el nombre	◯ Si<br>◯ No
â€¢ Caja de la medicina o botella vista (si/no)	◯ Si<br>◯ No

Alguna vez ha dejado de comer o tomar leche/carne/huevos del animal indicado, despuÃ©s de darle antibiÃ³ticos?

- ◯ SI
- ◯ No

Con que frecuencia les da esta medicina a sus animales? (Numero de veces al aÃ±o)

____

57. Tiene algun animal que vive adentro de la casa?

- ◯ a. Si
- ◯ b. No

58. Por favor indique el tipo y numero de animales que viven libremente adentro de la casa. (MARQUE TODOS LOS QUE APLIQUEN indicando cantidad)

- ☐ a. Perros
- ☐ b. Gatos
- ☐ c. Pollos
- ☐ d. Pavos
- ☐ e. Patos
- ☐ f. Gansos
- ☐ g. Otros pÃ¯Â¿Â½jaros
- ☐ h. Cerdos
- ☐ i. Vacas
- ☐ j. Ovejas
- ☐ k. Cabras
- ☐ a. Otros animales, especificar
- ☐ b. No sabe
- ☐ c. Se rehÃºsa a responder

Cual otro animal?

____

PERROS

Cuantos Perros?

____

Alguna vez les ha dado antibioticos a su/sus perro/perros?

- ◯ a. Si
- ◯ b. No
- ◯ c. No sabe
- ◯ d. Se rehÃºsa a responder

DATOS MEDICINA PERROS
Nombre de la Medicina/Antibiotico

____

â€¢ Medicina a la vista y se puede leer el nombre

- ◯ Si
- ◯ No

â€¢ Caja de la medicina o botella vista (si/no)

- ◯ Si
- ◯ No

Alguna vez ha dejado de tomar la leche o comer la carne del animal indicado, despues de darle antibioticos?

- ◯ SI
- ◯ No

Durante cuanto tiempo ha dejado de tomarla? (solo aplica si dice que si a la pregunta anterior)
(Numero de dias)

____

GATOS

Cuantos Gatos? ______________________________

Alguna vez les ha dado antibioticos a su/sus gato/gatos?
- ◯ a. Si
- ◯ b. No
- ◯ c. No sabe
- ◯ d. Se rehÃºsa a responder

DATOS MEDICINA GATOS
Nombre de la Medicina/Antibiotico ______________________________

â€¢ Medicina a la vista y se puede leer el nombre
- ◯ Si
- ◯ No

â€¢ Caja de la medicina o botella vista (si/no)
- ◯ Si
- ◯ No

Alguna vez ha dejado de tomar la leche o comer la carne del animal indicado, despues de darle antibioticos?
- ◯ SI
- ◯ No

Durante cuanto tiempo ha dejado de tomarla? (solo aplica si dice que si a la pregunta anterior) (Numero de dias) ______________________________

PAVOS

Cuantos Pavos? ______________________________

Alguna vez les ha dado antibioticos a su/sus pavo/pavos?
- ◯ a. Si
- ◯ b. No
- ◯ c. No sabe
- ◯ d. Se rehÃºsa a responder

DATOS MEDICINA PAVOS
Nombre de la Medicina/Antibiotico ______________________________

â€¢ Medicina a la vista y se puede leer el nombre
- ◯ Si
- ◯ No

â€¢ Caja de la medicina o botella vista (si/no)
- ◯ Si
- ◯ No

Alguna vez ha dejado de tomar la leche o comer la carne del animal indicado, despues de darle antibioticos?
- ◯ SI
- ◯ No

Durante cuanto tiempo ha dejado de tomarla? (solo aplica si dice que si a la pregunta anterior) (Numero de dias) ______________________________

POLLOS

Cuantos Pollos? ________________________________

Alguna vez les ha dado antibioticos a su/sus pollo/pollos?
- ◯ a. Si
- ◯ b. No
- ◯ c. No sabe
- ◯ d. Se rehÃºsa a responder

DATOS MEDICINA POLLOS
Nombre de la Medicina/Antibiotico ________________________________

â€¢ Medicina a la vista y se puede leer el nombre
- ◯ Si
- ◯ No

â€¢ Caja de la medicina o botella vista (si/no)
- ◯ Si
- ◯ No

Alguna vez ha dejado de tomar la leche o comer la carne del animal indicado, despues de darle antibioticos?
- ◯ SI
- ◯ No

Durante cuanto tiempo ha dejado de tomarla? (solo aplica si dice que si a la pregunta anterior)
(Numero de dias) ________________________________

PATOS

Cuantos Patos? ________________________________

Alguna vez les ha dado antibioticos a su/sus pato/patos?
- ◯ a. Si
- ◯ b. No
- ◯ c. No sabe
- ◯ d. Se rehÃºsa a responder

DATOS MEDICINA PATOS
Nombre de la Medicina/Antibiotico ________________________________

â€¢ Medicina a la vista y se puede leer el nombre
- ◯ Si
- ◯ No

â€¢ Caja de la medicina o botella vista (si/no)
- ◯ Si
- ◯ No

Alguna vez ha dejado de tomar la leche o comer la carne del animal indicado, despues de darle antibioticos?
- ◯ SI
- ◯ No

Durante cuanto tiempo ha dejado de tomarla? (solo aplica si dice que si a la pregunta anterior)
(Numero de dias) ________________________________

GANSOS

Cuantos Gansos? __________________________________

Alguna vez les ha dado antibioticos a su/sus ganso/gansos?
- ◯ a. Si
- ◯ b. No
- ◯ c. No sabe
- ◯ d. Se rehÃºsa a responder

DATOS MEDICINA GANSOS
Nombre de la Medicina/Antibiotico __________________________________

â€¢ Medicina a la vista y se puede leer el nombre
- ◯ Si
- ◯ No

â€¢ Caja de la medicina o botella vista (si/no)
- ◯ Si
- ◯ No

Alguna vez ha dejado de tomar la leche o comer la carne del animal indicado, despues de darle antibioticos?
- ◯ SI
- ◯ No

Durante cuanto tiempo ha dejado de tomarla? (solo aplica si dice que si a la pregunta anterior) (Numero de dias) __________________________________

PAJAROS

Cuantos otros pajaros? __________________________________

Alguna vez les ha dado antibioticos a su/sus pajaro/pajaros?
- ◯ a. Si
- ◯ b. No
- ◯ c. No sabe
- ◯ d. Se rehÃºsa a responder

DATOS MEDICINA PAJAROS
Nombre de la Medicina/Antibiotico __________________________________

â€¢ Medicina a la vista y se puede leer el nombre
- ◯ Si
- ◯ No

â€¢ Caja de la medicina o botella vista (si/no)
- ◯ Si
- ◯ No

Alguna vez ha dejado de tomar la leche o comer la carne del animal indicado, despues de darle antibioticos?
- ◯ SI
- ◯ No

Durante cuanto tiempo ha dejado de tomarla? (solo aplica si dice que si a la pregunta anterior) (Numero de dias) __________________________________

CERDOS

Cuantos Cerdos? ________________________

Alguna vez les ha dado antibioticos a su/sus cerdo/cerdos?
- ◯ a. Si
- ◯ b. No
- ◯ c. No sabe
- ◯ d. Se rehÃºsa a responder

DATOS MEDICINA CERDOS
Nombre de la Medicina/Antibiotico ________________________

â€¢ Medicina a la vista y se puede leer el nombre
- ◯ Si
- ◯ No

â€¢ Caja de la medicina o botella vista (si/no)
- ◯ Si
- ◯ No

Alguna vez ha dejado de tomar la leche o comer la carne del animal indicado, despues de darle antibioticos?
- ◯ SI
- ◯ No

Durante cuanto tiempo ha dejado de tomarla? (solo aplica si dice que si a la pregunta anterior)
(Numero de dias) ________________________

VACAS

Cuantos Vacas? ________________________

Alguna vez les ha dado antibioticos a su/sus vaca/vacas?
- ◯ a. Si
- ◯ b. No
- ◯ c. No sabe
- ◯ d. Se rehÃºsa a responder

DATOS MEDICINA VACAS
Nombre de la Medicina/Antibiotico ________________________

â€¢ Medicina a la vista y se puede leer el nombre
- ◯ Si
- ◯ No

â€¢ Caja de la medicina o botella vista (si/no)
- ◯ Si
- ◯ No

Alguna vez ha dejado de tomar la leche o comer la carne del animal indicado, despues de darle antibioticos?
- ◯ SI
- ◯ No

Durante cuanto tiempo ha dejado de tomarla? (solo aplica si dice que si a la pregunta anterior)
(Numero de dias) ________________________

CABRAS

Cuantos Cabras? ______________________

Alguna vez les ha dado antibioticos a su/sus cabra/cabras?
- ◯ a. Si
- ◯ b. No
- ◯ c. No sabe
- ◯ d. Se rehÃºsa a responder

DATOS MEDICINA CABRAS
Nombre de la Medicina/Antibiotico ______________________

â€¢ Medicina a la vista y se puede leer el nombre
- ◯ Si
- ◯ No

â€¢ Caja de la medicina o botella vista (si/no)
- ◯ Si
- ◯ No

Alguna vez ha dejado de tomar la leche o comer la carne del animal indicado, despues de darle antibioticos?
- ◯ SI
- ◯ No

Durante cuanto tiempo ha dejado de tomarla? (solo aplica si dice que si a la pregunta anterior)
(Numero de dias) ______________________

OVEJAS

Cuantos Ovejas? ______________________

Alguna vez les ha dado antibioticos a su/sus oveja/ovejas?
- ◯ a. Si
- ◯ b. No
- ◯ c. No sabe
- ◯ d. Se rehÃºsa a responder

DATOS MEDICINA OVEJAS
Nombre de la Medicina/Antibiotico ______________________

â€¢ Medicina a la vista y se puede leer el nombre
- ◯ Si
- ◯ No

â€¢ Caja de la medicina o botella vista (si/no)
- ◯ Si
- ◯ No

Alguna vez ha dejado de tomar la leche o comer la carne del animal indicado, despues de darle antibioticos?
- ◯ SI
- ◯ No

Durante cuanto tiempo ha dejado de tomarla? (solo aplica si dice que si a la pregunta anterior)
(Numero de dias) ______________________

a. Otros animales, especificar la cantidad ______________________

OTROS ANIMALES

Alguna vez les ha dado antibioticos a sus otros animales?
- ◯ a. Si
- ◯ b. No
- ◯ c. No sabe
- ◯ d. Se rehÃºsa a responder

DATOS MEDICINA OTROS ANIMALES
Nombre de la Medicina/Antibiotico ______________________

â€¢ Medicina a la vista y se puede leer el nombre
- ◯ Si
- ◯ No

â€¢ Caja de la medicina o botella vista (si/no)
- ◯ Si
- ◯ No

Alguna vez ha dejado de tomar la leche o comer la carne del animal indicado, despues de darle antibioticos?
- ◯ SI
- ◯ No

Con que frecuencia les da esta medicina a sus animales? (Numero de veces al aÃ±o) ______________________

59. Los animales a veces se quedan dentro de los cuartos en donde duermen los miembros de la familia(ya sea de dia o de noche)? (MARQUE UNO)
- ◯ a. Si
- ◯ b. No
- ◯ c. No sabe
- ◯ d. Se rehÃºsa a responder

i. Ovejas ______________________

60. Por favor indique Que animales se quedan a veces adentro de los cuartos donde duermen miembros de la familia (ya sea de noche o de dia)?
- ☐ a. Perros
- ☐ b. Gatos
- ☐ c. Pollos
- ☐ d. Pavos
- ☐ e. Gansos
- ☐ f. Otros pÃ¡jaros
- ☐ g. Cerdos
- ☐ h. Vacas
- ☐ i. Ovejas
- ☐ j. Cabras
- ☐ k. Otros, especificar
- ☐ l. No sabe
- ☐ m. RehÃºsa

a. Perros ______________________

b. Gatos ______________________

d. Pavos ______________________

g. Cerdos ______________________

c. Pollos ______________________

e. Gansos ______________________

h. Vacas ______________________

f. Otros pajaros ______________________

i. Ovejas ______________________

j. Cabras ______________________

k. Otros, especificar ______________________

SECCIÃ“N ALIMENTOS

61. Ustedes comen carne?
- ◯ a. Si
- ◯ b. No
- ◯ c. No sabe
- ◯ d. Se rehÃºsa a responder

62. Que tipo de carne? (Puede seleccionar mas de una)
- ☐ a. Pollo
- ☐ b. Carne de cerdo
- ☐ c. Carne de res
- ☐ d. Pescado
- ☐ e. Conejo
- ☐ f. Chompipe
- ☐ g. Pato
- ☐ h. Otro, indique
- ☐ i. No sabe
- ☐ j. Se rehÃºsa a responder

Otro ______________________

63. Indique la procedencia de cada tipo de carne
- ☐ a. Criado en la casa
- ☐ b. Comprado en el mercado
- ☐ c. Comprado en el supermercado
- ☐ d. Comprado en la despensa
- ☐ e. Comprado en la tienda
- ☐ f. Otro, especificar
- ☐ g. No sabe
- ☐ h. Se rehÃºsa a responder

Otro ______________________

64. Ustedes toman leche?
- ◯ a. Si
- ◯ b. No
- ◯ c. No sabe
- ◯ d. Se rehÃºsa a responder

65. Que tipo de leche toman? (MARQUE TODOS LOS QUE APLIQUEN)
- ☐ a. Leche de vaca (en fresco)
- ☐ b. Leche en polvo
- ☐ c. Leche en caja
- ☐ d. Leche de cabra (en fresco)
- ☐ e. Leche en bolsa
- ☐ f. Otro

66. Hierve la leche antes de tomarla?
- ◯ a. Si
- ◯ b. No
- ◯ c. No sabe
- ◯ d. Se rehÃºsa a responder

67. Cuanta leche hervida toma por semana? (numero de vasos enteros) ______________________

Otro ______________________

68. Cuanta leche no hervida se toma por semana (numero de vasos enteros)? ______________________

69. Cuanta leche comprada en la tienda/supermercado toma por semana? (numero de vasos enteros) ______________________

70. Usted guarda la leche?
- ◯ a. Si
- ◯ b. No

71. En donde guarda la leche?
- ◯ a. En la refri
- ◯ b. Sin refrigeraciÃ³n
- ◯ c. No sabe
- ◯ d. Se rehÃºsa a responder

72. En que tipo de recipiente guarda la leche?
- ◯ a. En su recipiente original desechable (comprado en la tienda/supermercado)
- ◯ b. PlÃ¡stico
- ◯ c. Metal
- ◯ d. Vidrio
- ◯ e. Bolsa
- ◯ f. No sabe
- ◯ g. Se rehÃºsa a responder

73. Es un recipiente especifico para la leche?
- ◯ a. Si
- ◯ b. No
- ◯ c. No sabe
- ◯ d. Se rehÃºsa a responder

74. Lava el recipiente antes de agregarle la leche?
- ◯ a. Si
- ◯ b. No
- ◯ c. No sabe
- ◯ d. Se rehÃºsa a responder

75. Como lava el recipiente? (marque los que aplican)
- ◯ a. Con agua y jabÃ³n
- ◯ b. Ãšnicamente con desinfectante (ej: cloro)
- ◯ c. Con agua, jabÃ³n y desinfectante
- ◯ d. Hervir el recipiente
- ◯ e. No sabe
- ◯ f. Se rehÃºsa a responder

Cuestionario Adultos

Preguntas de tamizaje - para adulto que darÃi la muestra

1. Usted ha vivido en la casa durante por lo menos un aÃ±o?
   - ◯ a. SÃ-
   - ◯ b. No

2. Usted vivira en la misma casa por lo menos un aÃ±o mas?
   - ◯ a. SÃ-
   - ◯ b. No

3. Usted esta enfermo el dia de hoy con diarrea, fiebre aguda o una enfermedad respiratoria?
   - ◯ a. SÃ-- Finalizado
   - ◯ b. No

4. Usted esta tomando algun medicamento el dia de hoy?
   - ◯ a. SÃ-
   - ◯ b. No

5. Nos puede enseÃ±ar que medicamento esta tomando?
   - ◯ a. SÃ-
   - ◯ b. No, no lo puede mostrar

5.1 Nos puede decir cual medicamento esta tomando?
   - ◯ a. SÃ-.
   - ◯ b. No, no lo puede decir. Finalizado.

'' El encuestador debe verificar si se trata de un antibiÃƒÂ³tico o no. Puede ayudarse con el ÃƒÂ¡lbum de medicamentos o, en el caso de dificultad, llamando a Brooke o a Puri''

6. Es un antibiÃ³tico lo que estÃi tomando el adulto en el dÃ-a de hoy?
   - ◯ a. SÃ-, Finalizado
   - ◯ b. No - proceder al consentimiento informado

ELEGIBLE O NO ________________________________

7. Usted dio su consentimiento informado?
   - ◯ a. SÃ-
   - ◯ b. No- Finalizado

Cuestionario para la EvaluaciÃ³n comunitaria de Escherichia coli resistente a los antibiÃ³ticos en Quetzaltenango; Guatemala

INFORMACIÃ“N DEMOGRÃ FICA del adulto

1. Codigo/identificacion (xxx-xx) ________________________________

Latitud ________________________________

Longitud ________________________________

2. Localidad
   - ◯ Coatepeque
   - ◯ San Juan Ostuncalco

a. Coatepeque (listado definido de opciones)
   - ◯ a. El Jardin
   - ◯ b. La Union

b. San Juan Ostuncalco (listado definido de opciones)
   - ◯ 1.Casco Urbano
   - ◯ 2.Monrovia

3. Sexo
   - ◯ a. Masculino
   - ◯ b. Femenino

4. Cuantos aÃ±os tiene? (anotar # aÃ±os completos) ______________________________

5. De que etnia se considera usted?
- a. IndÃ-gena
- b. Ladino
- c. Otro. Especificar.
- d. Desconocido
- e. Se rehÃ¯Â¿Â½sa a responder

Otro ______________________________

6. Si se identifica como indigena, A cual grupo etnico pertenece usted?
- a. Maya
- b. Xinca
- c. Ladino
- d. GarÃ-funa
- e. Mestizo
- f. Otro
- g. Se rehÃºsa a responder

Otro ______________________________

7. Sabe usted leer y escribir?
- a. Si
- b. No
- c. Se rehÃºsa a responder

8. CuÃ¡l fue el nivel acadÃ©mico que usted completÃ©?
- a. Ninguna educaciÃ³n formal (no fue al colegio)
- b. No completÃ³ educaciÃ³n primaria
- c. CompletÃ³ educaciÃ³n primaria
- d. CompletÃ³ educaciÃ³n secundaria
- e. CompletÃ³ educaciÃ³n mayor a la secundaria
- f. Otro
- g. No quiere responder

8.1 Â¿CuÃ¡l fue el nivel acadÃ©mico que usted completÃ³? ______________________________

9. Con que frecuencia toma alcohol?
- a. No toma alcohol
- b. De vez en cuando (ocasional)
- c. Mensual
- d. Semanal
- e. Diario
- f. No quiere responder

I. PrÃ¡cticas en la Vivienda: Lavado de manos

ENCUESTADOR LEA: Ahora me gustarÃ-a preguntarle sobre los hÃ¡bitos de higiene en el hogar. Estas preguntas tienen que ver principalmente con el lavado de manos en su hogar.

10. Se ha lavado las manos con agua por cualquier razon el dia de hoy? MARQUE UNO
- a. Si
- b. No
- c. No sabe
- d. Se rehÃºsa a responder

11. De que fuente salio el agua con la que se lavo las manos?
- a. CaÃ¯Â¿Â½a agua del chorro
- b. UsÃ¯Â¿Â½ agua de un bote
- c. Otra fuente de agua
- d. No sabe
- e. Se rehÃ¯Â¿Â½sa a responder

11. 1 Otra fuente de agua ______________________________

12. Uso jabon?

- a. SÃ-
- b. No
- c. No sabe
- d. Se rehÃºsa a responder

II.Historia de uso antibiÃ³tico

En la siguiente secciÃ³n de la encuesta le preguntaremos sobre el uso de medicamentos, especialmente, el uso de antibiÃ³ticos. Los antibiÃ³ticos se definen como medicamentos usados para matar las bacterias

13. Ha tomado antibioticos alguna vez?

- a. Si
- b. No
- c. No sabe
- d. Se rehÃºsa a responder

14. Ha tomado antibioticos en los ultimos 14 dias?

- a. Si
- b. No
- c. No sabe
- d. Se rehÃƒÂºsa a responder

15. La ultima vez que tomo un antibiotico, para que enfermedad lo tomo? (puede seleccionar mas de una)

- a. Nariz congestionada
- b. Tos
- c. Dolor de cabeza
- d. Fiebre
- e. VÃƒÂ³mitos
- f. Diarrea
- g. Heridas de la piel
- h. Malaria
- i. VIH
- j. Zika
- k. Dengue
- l. Chikungunya
- m. Tuberculosis
- n. Otro. Especificar.
- o. No Sabe
- p. Se rehÃºsa a responder

Otro ________________________________

16. Hablando de la enfermedad que le dio en la P15, Â¿BuscÃ³ algÃºn tipo de atenciÃ³n para tratar su enfermedad?

- a. SÃ- (ir a P17)
- b. No (ir a P18)
- c. No sabe (ir a P18)
- d. Se rehÃºsa a responder (ir a P18)

17. Donde busco atencion? (Marque todos los que apliquen)

- ☐ a. Puesto de Salud
- ☐ b. Centro de Salud
- ☐ c. Hospital PÃºblico
- ☐ d. Hospital Privado
- ☐ e. ClÃ-nica Privada
- ☐ f. Centro de Convergencia
- ☐ g. Trabajador de Salud
- ☐ h. Feria MÃ©dica
- ☐ i. Farmacia
- ☐ j. Tienda
- ☐ k. Curandero
- ☐ l. Miembros de la Familia y/o amigos
- ☐ m. Otros, especificar
- ☐ n. No sabe
- ☐ o. Se rehÃºsa a responder

Otro ________________________________

18. Â¿QuÃ© medicamentos usÃ³ para esta enfermedad en particular (pregunte para cada enfermedad mencionada en P16). Pida ver los medicamentos si estÃ¡n disponibles, y pregunte "Â¿quÃ© otros medicamento tomÃ³?" hasta que ya no mencionen otros medicamentos.

- ☐ a. AntibiÃ³ticos
- ☐ b. Desparasitantes
- ☐ c. Medicamentos para el dolor
- ☐ d. Medicamentos naturales
- ☐ e. Otros,
- ☐ f. No sabe
- ☐ g. Se rehÃºsa a responder

Otro ________________________________

19. Â¿CuÃ¡ntos tipos de antibiÃ³ticos ha tomado? ________________________________

20. Para cada antibiÃ³tico complete la siguiente tabla:

DATOS MEDICINA No 1
Nombre de la medicina (1) ________________________________

â€¢ Se examino paquete o botella (si/no)

- ○ Si
- ○ No

â€¢ El nombre del medicamento se puede leer

- ○ Si
- ○ No

Le recetaron este medicamento en un puesto/centro/clinica privada de salud?

- ○ Si
- ○ No
- ○ No se
- ○ No Responde

â€¢ Si, indique en que lugar se le receto/recomendo.

- ○ Centro de salud MSPAS
- ○ Puesto de salud/ClÃ-nica privada

Si no, como sabia que medicamento comprar?

- ○ Amigo o familiar
- ○ Experiencia previa
- ○ Persona en la farmacia/mÃ¯Â¿Â½dico/otro

En donde consiguio el medicamento?

- ◯ Farmacia
- ◯ Tienda
- ◯ Super
- ◯ MSPAS
- ◯ Amigo o familiar
- ◯ Comadronas
- ◯ Curanderos
- ◯ Otros

Cuantos dias tomo el medicamento? ________________________________

Con que frecuencia toma antibioticos?

- ◯ Cada mes
- ◯ Cada tres meses
- ◯ Cada 6 meses
- ◯ Cada aÃ±o
- ◯ Cada vez que es necesario
- ◯ No sabe
- ◯ No responde

Ademas de usted, uso otra persona o animal este medicamento?

- ◯ SI
- ◯ No

â€¢ Si si, que otra persona o animal uso este medicamento?

- ☐ a. PapÃi o MamÃi
- ☐ b, Pareja
- ☐ c. Hermanos(as)
- ☐ d. Hijos
- ☐ e. Abuelos
- ☐ f. Otro parientes
- ☐ g. Animal

Medicina 2

Nombre de la medicina ________________________________

â€¢ Se examino paquete o botella (si/no)

- ◯ Si
- ◯ No

â€¢ El nombre del medicamento se puede leer

- ◯ Si
- ◯ No

Le recetaron este medicamento en un puesto/centro/clinica privada de salud?

- ◯ Si
- ◯ No
- ◯ No se
- ◯ No Responde

â€¢ Si, indique en que lugar se le receto/recomendo.

- ◯ Centro de salud MSPAS
- ◯ Puesto de salud/ClÃ-nica privada

Si no, como sabia que medicamento comprar?

- ◯ Amigo o familiar
- ◯ Experiencia previa
- ◯ Persona en la farmacia/mÃ¯Â¿Â½dico/otro

En donde consiguio el medicamento?

- ◯ Farmacia
- ◯ Tienda
- ◯ Super
- ◯ MSPAS
- ◯ Amigo o familiar
- ◯ Comadronas
- ◯ Curanderos
- ◯ Otros

Cuantos dias tomo el medicamento? ______________________________

Con que frecuencia toma antibioticos?

- ◯ Cada mes
- ◯ Cada tres meses
- ◯ Cada 6 meses
- ◯ Cada aÃ±o
- ◯ Cada vez que es necesario
- ◯ No sabe
- ◯ No responde

Ademas de usted, uso otra persona o animal este medicamento?

- ◯ SI
- ◯ No

â€¢ Si si, que otra persona o animal uso este medicamento?

- ☐ a. PapÃi o MamÃi
- ☐ b, Pareja
- ☐ c. Hermanos(as)
- ☐ d. Hijos
- ☐ e. Abuelos
- ☐ f. Otro parientes
- ☐ g. Animal

Medicina 3

Nombre de la medicina ______________________________

â€¢ Se examino paquete o botella (si/no)

- ◯ Si
- ◯ No

â€¢ El nombre del medicamento se puede leer

- ◯ Si
- ◯ No

Le recetaron este medicamento en un puesto/centro/clinica privada de salud?

- ◯ Si
- ◯ No
- ◯ No se
- ◯ No Responde

Indique en que lugar se le receto/recomendo.

- ◯ Centro de salud MSPAS
- ◯ Puesto de salud/ClÃ¯Â¿Â½nica privada

Si no, como sabia que medicamento comprar?

- ◯ Amigo o familiar
- ◯ Experiencia previa
- ◯ Persona en la farmacia/mÃ¯Â¿Â½dico/otro

En donde consiguio el medicamento?
- ◯ Farmacia
- ◯ Tienda
- ◯ Super
- ◯ MSPAS
- ◯ Amigo o familiar
- ◯ Comadronas
- ◯ Curanderos
- ◯ Otros

Cuantos dias tomo el medicamento? ______________________________

Con que frecuencia toma antibioticos?
- ◯ Cada mes
- ◯ Cada tres meses
- ◯ Cada 6 meses
- ◯ Cada aÃ±o
- ◯ Cada vez que es necesario
- ◯ No sabe
- ◯ No responde

Ademas de usted, uso otra persona o animal este medicamento?
- ◯ SI
- ◯ No

â€¢ Si si, que otra persona o animal uso este medicamento?
- ☐ a. PapÃi o MamÃi
- ☐ b, Pareja
- ☐ c. Hermanos(as)
- ☐ d. Hijos
- ☐ e. Abuelos
- ☐ f. Otro parientes
- ☐ g. Animal

III.PrÃƒÂicticas de la vivienda incluyendo posesiÃƒÂ³n de animales, mÃƒÂ©todos de cocciÃƒÂ³n y dieta

20. Usted come carne?
- ◯ a. Si
- ◯ b. No
- ◯ c. No sabe
- ◯ d. Se rehÃºsa a responder

21. Que tipo de carne? (Puede seleccionar mas de una)
- ☐ a. Pollo
- ☐ b. Carne de cerdo
- ☐ c. Carne de res
- ☐ d. Pescado
- ☐ e. Conejo
- ☐ f. Chompipe
- ☐ g. Pato
- ☐ h. Otro, indique
- ☐ i. No sabe
- ☐ j. Se rehÃºsa a responder

21.1 QuÃ© otro tipo de carne? ______________________________

22. indique la procedencia de cada tipo de carne indicado en la P21:

- ☐ a. Criado en la casa
- ☐ b. Comprado en el mercado
- ☐ c. Comprado en el supermercado
- ☐ d. Comprado en la despensa
- ☐ e. Comprado en la tienda
- ☐ f. Otro, especificar
- ☐ g. No sabe
- ☐ h. Se rehÃ¯Â¿Â½sa a responder

22.1 Otro

____

23. Usted toma leche?

- ◯ a. Si
- ◯ b. No
- ◯ c. No sabe
- ◯ d. Se rehÃºsa a responder

24. Que tipo de leche toma? (MARQUE TODOS LOS QUE APLIQUEN)

- ☐ a. Leche de vaca (en fresco)
- ☐ b. Leche en polvo
- ☐ c. Leche en caja
- ☐ d. Leche de cabra (en fresco)
- ☐ e. Leche en bolsa
- ☐ f. Otro

Otro

____

25. Trata (o prepara) de alguna forma la leche antes de tomarla?

- ◯ a. Si
- ◯ b. No
- ◯ c. No sabe
- ◯ d. Se rehÃ¯Â¿Â½sa a responder

26. Hierve la leche antes de tomarla?

- ◯ a. Si
- ◯ b. No
- ◯ c. No sabe
- ◯ d. Se rehÃ¯Â¿Â½sa a responder

27. Cuanta leche hervida toma por semana? (numero de vasos enteros)

- ◯ a. NÃºmero de vasos
- ◯ b. No sabe
- ◯ Se rehÃºsa a responder

Cuantos vasos por semana

____

28. Cuanta leche no hervida se toma por semana (numero de vasos enteros)?

- ◯ a. NÃºmero de vasos
- ◯ b. No sabe
- ◯ Se rehÃºsa a responder

Cuantos vasos por semana

____

29. Cuanta leche comprada en la tienda/supermercado toma por semana (numero de vasos enteros)?

- ◯ a. NÃºmero de vasos
- ◯ b. No sabe
- ◯ c. Se rehÃºsa a responder

Cuantos vasos por semana

____

IV.FACTORES RELACIONADOS A VIAJES

30. En los ultimos 14 dias, ha salido fuera de su municipio dentro del departamento Quetzaltenango?

- ◯ a. Si
- ◯ b. No

31. A que municipio?

- ◯ a. San Carlos Sija
- ◯ b. CabricÃ¯Â¿Â½n
- ◯ c. HuitÃ¯Â¿Â½n
- ◯ d. Sibilia
- ◯ e. CajolÃ¯Â¿Â½
- ◯ f. Palestina de los Altos
- ◯ g. San Miguel SigÃ¯Â¿Â½ila
- ◯ h. San Juan Ostuncalco
- ◯ i. San mateo
- ◯ j. San Francisco La UniÃ¯Â¿Â½n
- ◯ k. La Esperanza
- ◯ l. Olintepeque
- ◯ m. SalcajÃ¯Â¿Â½
- ◯ n. Quetzaltenango
- ◯ o. Almolonga
- ◯ p. Cantel
- ◯ q. Zunil
- ◯ r. El Palmar
- ◯ s. ConcepciÃ¯Â¿Â½n Chiquirichapa
- ◯ t. San Martin SacatepÃ¯Â¿Â½quez
- ◯ u. Colomba
- ◯ v. Flores Costa Cuca
- ◯ w. Coatepeque

32. En este viaje, Cuanto tiempo estuvo fuera de su casa?

- ◯ a. Una semana o menos
- ◯ b. MÃ¡s de una semana, pero menos de un mes
- ◯ c. Un mes o mÃ¡s

33. Con que frecuencia viaja a esa localidad? (NÃƒÂºmero de veces)

____

33.1. Con que frecuencia viaja a esa localidad? (Veces por)

- ◯ Semana
- ◯ Mes
- ◯ AÃ±o

34. En los ultimos 14 dias Ha ido a algun departamento fuera de Quetzaltenango?

- ◯ a. Si
- ◯ b. No
- ◯ c. No sabe
- ◯ d. Se rehÃºsa a responder

35. A que departamento?

- ◯ a. Alta Verapaz
- ◯ b. Baja Verapaz
- ◯ c. Chimaltenango
- ◯ d. Chiquimula
- ◯ e. El PetÃ¯Â¿Â½n
- ◯ f. El Progreso
- ◯ g. El QuichÃ¯Â¿Â½
- ◯ h. Escuintla
- ◯ i. Guatemala
- ◯ j. Huehuetenango
- ◯ k. Izabal
- ◯ l. Jalapa
- ◯ m. Jutiapa
- ◯ n. Quetzaltenango
- ◯ o. Retalhuleu
- ◯ p. SacatepÃ¯Â¿Â½quez
- ◯ q. San Marcos
- ◯ r. Santa Rosa
- ◯ s. SololÃ¯Â¿Â½
- ◯ t. SuchitepÃ¯Â¿Â½quez
- ◯ u. TotonicapÃ¯Â¿Â½n
- ◯ v. Zacapa

36. En su ultimo viaje al departamento X, cuanto tiempo estuvo fuera de su casa?
- ◯ a. Una semana o menos
- ◯ b. MÃis de una semana, pero menos de un mes
- ◯ c. Un mes o mÃis

37. Con que frecuencia viaja a ese departamento? (NÃƒÂºmero de veces) __________________________________

37.1 Con que frecuencia viaja a ese departamento? (Veces por)
- ◯ Semana
- ◯ Mes
- ◯ AÃƒÂ±o

38. En los ultimos 14 dias Ha ido a otro pais fuera de Guatemala?
- ◯ a. Si
- ◯ b. No

39. A que pais?
- ◯ a. MÃ¯Â¿Â½xico
- ◯ b. El Salvador
- ◯ c. Honduras
- ◯ d. Belice
- ◯ e. Estados Unidos
- ◯ f. Otro.

39.1 Â¿Cual otro paÃ-s? __________________________________

40. En el ultimo viaje a otro pais, cuanto tiempo estuvo fuera de su casa?
- ◯ a. Una semana o menos
- ◯ b. MÃis de una semana, pero menos de un mes
- ◯ c. Un mes o mÃis

41. Con que frecuencia viaja a ese paÃ-s? (NÃºmero de veces) __________________________________

41.1 Con que frecuencia viaja a ese pais? (Veces por)
- ◯ Semana
- ◯ Mes
- ◯ AÃƒÂ±o

IV. Enfermedades Previas-Diarrea y exposiciÃ³n a hospitales: Ahora quisiera preguntarle sobre eventos previos de enfermedad diarreica.

42. En los ultimos 14 dias, ha tenido diarrea?
- ◯ a. Si
- ◯ b. No
- ◯ c. No sabe
- ◯ d. Se rehÃºsa a responder

43. Hace cuantos dias tuvo su ultimo episodio de diarrea/asientos? __________________________________

44. Durante el peor dÃ-a de diarrea/asientos: cuantas veces fue al baÃ±o? __________________________________

45. Durante este episodio de diarrea/asientos, tuvo sintomas de vomitos?
- ◯ a. Si
- ◯ b. No
- ◯ c. No sabe
- ◯ d. Se rehÃºsa a responder

46. Busco algun tipo de atencion fuera de la casa para curar su diarrea?
- ◯ a. Si
- ◯ b. No
- ◯ c. No sabe
- ◯ d. Se rehÃ¯Â¿Â½sa a responder

47. Si busco algun tipo de atencion fuera de la casa, a donde acudio? (marque todos los que apliquen)

- ☐ a. Puesto de Salud
- ☐ b. Centro de Salud
- ☐ c. Hospital PÃºblico
- ☐ d. Hospital Privado
- ☐ e. ClÃ¯Â¿Â½nica Privada
- ☐ f. Centro de Convergencia
- ☐ g. Trabajador de Salud
- ☐ h. Feria MÃ©dica
- ☐ i. Farmacia
- ☐ j. Tienda
- ☐ k. Curandero
- ☐ l. Miembros de la Familia y/o amigos
- ☐ m.Otros

Otro ______________________________

48. Tomo algun medicamento para tratar este episodio de diarrea/asientos?

- ◯ a. Si
- ◯ b. No
- ◯ c. No sabe
- ◯ d. Se rehusa a responder

49. Que medicamentos tomo?

- ☐ a. Medicamentos para el dolor
- ☐ b. Desparasitantes
- ☐ c. Remedios de hierbas
- ☐ d. Otros
- ☐ e. Ninguno

Nombre del medicamento ______________________________

Remedios de hierbas: ______________________________

50. Nos menciono usted estos antibioticos en las preguntas que le hicimos anteriormente?

- ◯ a. Si
- ◯ b. No
- ◯ c. No sabe
- ◯ d. Se rehÃºsa a responder

51. Usted trabaja en el hospital?

- ◯ a.Si
- ◯ b.No

52. En los ultimos 3 meses, ha visitado el hospital por alguna razon?

- ◯ a. Si
- ◯ b. No
- ◯ c. No sabe
- ◯ d. RehÃºsa

53. Por que visito el hospital?

- ◯ a. Para hacer un tramite
- ◯ b. para visitar a un paciente internado/AcompaÃ±ar a un familiar
- ◯ c. Para un chequeo mÃ©dico rutinario
- ◯ d. Para recibir un tratamiento
- ◯ e. Porque estaba enfermo y me internaron
- ◯ f. Otro
- ◯ g. No sabe
- ◯ h. Se rehÃºsa a responder

Otro ______________________________

54. En los ultimos 3 meses, ha visitado el centro de salud por alguna razon?

- ◯ a. Si
- ◯ b. No
- ◯ c. No sabe
- ◯ d. Se rehÃºsa a responder

55. Por que visito el centro de salud?

- ◯ a. Para hacer un tramite
- ◯ b. para visitar a un paciente internado/AcompaÃ±ar a un familiar
- ◯ c. Para un chequeo mÃ©dico rutinario
- ◯ d. para recibir un tratamiento
- ◯ e. porque estaba enferma y me internaron
- ◯ f. Otro, especificar
- ◯ g. No sabe
- ◯ h. Se rehÃºsa a responder

Otro ________________________________

Cuestionario NiÃ±o menor de 5 AÃ±os

Preguntas de tamizaje - Gracias por su participaciÃ³n en el proyecto. Como le comentamos al principio, tambiÃ©n estamos interesados en la participaciÃ³n de un niÃ±o menor de 5 aÃ±os que viva en su hogar. Ahora le haremos unas preguntas sobre su niÃ±o. Estas preguntas son iguales a las que le hicimos a usted.

1. Â¿El niÃ±o ha vivido en la casa durante por lo menos un aÃ±o (o desde su nacimiento)?
   - ○ a. SÃ-
   - ○ b. No

2. Â¿El niÃ±o vivirÃi en la misma casa por lo menos un aÃ±o mÃis?
   - ○ a. Si
   - ○ b. No

3. Â¿El niÃ±o estÃi enfermo el dÃ-a de hoy con diarrea, fiebre aguda o una enfermedad respiratoria severa?
   - ○ a. SÃ-- Finalizado
   - ○ b. No

4. Â¿El niÃ±o estÃi tomando algÃºn medicamento el dÃ-Â-a de hoy?
   - ○ a. SÃ-
   - ○ b. No

5. Nos puede enseÃ±ar quÃ© medicamento estÃi tomando?
   - ○ SÃ-Â-, consultar el listado para ver si se trata de un antibiÃ³tico. Si estÃi en la lista de antibiÃ³ticos no puede participar. Si no estÃi en la lista, llamar a Puri y Brooke para aclarar si se trata de un antibiÃ³tico o no
   - ○ No, no lo puede mostrar

5.1 Nos puede decir cual medicamento esta tomando?
   - ○ a. SÃ-.
   - ○ b. No, no lo puede decir. Finalizado.

'' El encuestador debe verificar si se trata de un antibiÃƒÂ³tico o no. Puede ayudarse con el ÃƒÂ¡lbum de medicamentos o, en el caso de dificultad, llamando a Brooke o a Puri''

6. Â¿Es un antibiÃ³tico lo que estÃi tomando el niÃ±oo en el dÃ-Â-a de hoy?
   - ○ a. SÃ-, Finalizado
   - ○ b. No - proceder al consentimiento informado

ELEGIBLE O NO ________________________________

7. Â¿El encargado del niÃ±o dio su consentimiento informado para que el niÃ±o participe?
   - ○ a. SÃ-
   - ○ b. No- Finalizado

INFORMACIÃ“N DEMOGRÃFICA del niÃ±o participante

1. Codigo/identificacion (xxx-xx) ________________________________

Latitud ________________________________

Longitud ________________________________

2. Localidad
   - ○ Coatepeque
   - ○ San Juan Ostuncalco

a. Coatepeque (listado definido de opciones)
   - ○ a. El Jardin
   - ○ b. La Union

b. San Juan Ostuncalco (listado definido de opciones)
- ◯ 1.Casco Urbano
- ◯ 2.Monrovia

3. Sexo
- ◯ a. Masculino
- ◯ b. Femenino

4. Â¿CuÃ¡ntos aÃ±os tiene el niÃ±o? (anotar # aÃ±os completos) ________________________________

I.Factores en el Medio Ambiente: Fuentes de Agua, construcciÃ³n de casa, manejo de basura

5. La Ãºltima vez el niÃ±o defecÃ³, Â¿cÃ³mo desecharon las heces?
- ◯ a. El niÃ±o uso el inodoro/letrina
- ◯ b. Se pusieron adentro del inodoro o letrina
- ◯ c. Se pusieron adentro/enjuagaron en el desagÃ¼e o zanja (o lo lavaron en la pila)
- ◯ d. Se tiraron a la basura
- ◯ e. Se enterraron
- ◯ f. Se dejaron en el patio al aire libre
- ◯ g. Se dejaron afuera del lote al aire libre
- ◯ h. Otro
- ◯ i. No sabe
- ◯ j. Se rehÃºsa a responder

Otro ________________________________

6. Â¿Usa el niÃ±o el agua del rÃ-Â-o en su casa?
- ◯ a. Si
- ◯ b. No
- ◯ c. No sabe
- ◯ d. Se rehÃºsa a responder

7. Â¿Para quÃ© se usa el niÃ±o el agua del rÃ-o en su casa?
- ☐ a. Para jugar adentro del rÃ-o
- ☐ b. Para jugar con el agua afuera del rÃ-o
- ☐ c. Para ayudar a cocinar
- ☐ d. Cuando ayuda en lavar platos
- ☐ e. Cuando ayuda a lavar ropa
- ☐ f. BaÃ±arse
- ☐ g. Para ayudar a regar jardines
- ☐ h. No sabe
- ☐ i. Se rehÃºsa a responder

II. PrÃ¡cticas en la Vivienda: Lavado de manos

ENCUESTADOR LEA: Ahora me gustarÃ-a preguntarle sobre higiene en los hÃ¡bitos del hogar. Estas preguntas tienen que ver principalmente con el lavado de manos del niÃ±o en su hogar.

8. Â¿Se ha lavado las manos el niÃ±o con agua por cualquier razÃ³n el dÃ-a de hoy? (MARQUE UNO)
- ◯ a. Si
- ◯ b. No
- ◯ c. No sabe
- ◯ d. Se rehÃºsa a responder

9. Â¿De quÃ© fuente saliÃ³ el agua con que se lavÃ³ las manos?
- ◯ a. Con agua del chorro
- ◯ b. Con agua de un bote (agua estancada)
- ◯ c. Otra fuente de agua
- ◯ d. No sabe
- ◯ e. Se rehÃºsa a responder

9. 1 Otra fuente de agua ________________________________

10. Uso jabon?

- ◯ a. SÃ-
- ◯ b. No
- ◯ c. No sabe
- ◯ d. Se rehÃºsa a responder

III. PrÃ¡cticas de la vivienda incluyendo posesiÃ³n de animales, mÃ©todos de cocciÃ³n y dieta

11. Â¿El niÃ±o come carne?

- ◯ a. Si
- ◯ b. No
- ◯ c. No sabe
- ◯ d. Se rehÃºsa a responder

12.Â¿QuÃ© tipo de carne? (puede responder a varios)

- ☐ a. Pollo
- ☐ b. Carne de cerdo
- ☐ c. Carne de res
- ☐ d. Pescado
- ☐ e. Conejo
- ☐ f. Chompipe
- ☐ g. Pato
- ☐ h. Otro, indique
- ☐ i. No sabe
- ☐ j. Se rehÃšsa a responder

12.1 QuÃ© otro tipo de carne? ___________________________________

13. indique la procedencia de cada tipo de carne que el niÃ±o come

- ☐ a. Criado en la casa
- ☐ b. Comprado en el mercado
- ☐ c. Comprado en el supermercado
- ☐ d. Comprado en la despensa
- ☐ e. Comprado en la tienda
- ☐ f. Otro, especificar
- ☐ g. No sabe
- ☐ h. Se rehÃºsa a responder

13.1 Otro ___________________________________

14. Â¿El niÃ±o toma leche?

- ◯ a. Si
- ◯ b. No
- ◯ c. No sabe
- ◯ d. Se rehÃºsa a responder

15. Â¿QuÃ© tipo de leche toma?

- ☐ a. Leche Materna
- ☐ b. Leche de vaca (en fresco)
- ☐ c. Leche en polvo
- ☐ d. Leche en caja
- ☐ e. Leche de cabra (en fresco)
- ☐ f. Leche en bolsa
- ☐ g. Otro

Otro ___________________________________

16. Â¿Trata (o prepara) de alguna forma la leche de vaca o cabra (en fresco) antes de tomarla?

- ◯ a. Si
- ◯ b. No
- ◯ c. No sabe
- ◯ d. Se rehÃºsa a responder

17. Â¿Hierve la leche antes de tomarla?

- ◯ a. Si
- ◯ b. No
- ◯ c. No sabe
- ◯ d. Se rehÃºsa a responder

18. Â¿CuÃ¡nta leche hervida toma por semana? (nÃºmero de vasos/pachas enteros)

- ◯ a. NÃºmero de vasos/pachas
- ◯ b. No sabe
- ◯ Se rehÃºsa a responder

Cuantos vasos/pachas por semana ________________________________

19. Â¿CuÃ¡nta leche no hervida se toma por semana (nÃºmero de vasos/pachas enteros)?

- ◯ a. NÃºmero de vasos/pachas
- ◯ b. Nada
- ◯ c. No sabe
- ◯ Se rehÃºsa a responder

Cuantos vasos/pachas por semana ________________________________

20. Â¿Cuanta leche comprada en la tienda/supermercado toma por semana (nÃºmero de vasos/pachas enteros)?

- ◯ a. NÃºmero de vasos/pachas
- ◯ b. No sabe
- ◯ c. Se rehÃºsa a responder

Cuantos vasos/pachas por semana ________________________________

21. Â¿Usted guarda la leche que toma su niÃ±o?

- ◯ a. Si
- ◯ b. No

22. Â¿En dÃ³nde se guarda la leche?

- ◯ a. En frÃo
- ◯ b. No en friÃ³
- ◯ c. No sabe
- ◯ d. Se rehÃºsa a responder

23. Â¿En quÃ© tipo de recipiente guarda la leche?

- ◯ a. En su recipiente original desechable (comprado en la tienda/supermercado) (ir a P27)
- ◯ b. PlÃ¡stico (ir a P24)
- ◯ c. Metal (ir a P24)
- ◯ d. Vidrio (ir a P24)
- ◯ e. Bolsa (ir a P24)
- ◯ f. No sabe (ir a P27)
- ◯ g. Se rehÃºsa a responder (ir a P27)

24. Â¿Es un recipiente dedicado Ãºnicamente para la leche?

- ◯ a. Si
- ◯ b. No
- ◯ c. No sabe
- ◯ d. Se rehÃºsa a responder

25. Â¿Lava el recipiente antes de agregarle leche?

- ◯ a. Si
- ◯ b. No
- ◯ c. No sabe
- ◯ d. Se rehÃºsa a responder

26. Ã‚Â¿CÃƒÂ³mo lava el recipiente? (marque los que aplican)

- ☐ a. Con agua y jabÃƒÂ³n
- ☐ b. ÃƒÅ¡nicamente con desinfectante (ej: cloro)
- ☐ c. Con agua, jabÃƒÂ³n y desinfectante
- ☐ d. Hervir el recipiente
- ☐ e. No sabe
- ☐ f. Se rehÃƒÂºsa a responder

IV.FACTORES RELACIONADOS A VIAJES

27. En los Ãºltimos 14 dÃas, Â¿el niÃ±o ha salido fuera de su municipio dentro del departamento de Quetzaltenango?

- ◯ a. Si
- ◯ b. No

28. A que municipio?

- ◯ a. San Carlos Sija
- ◯ b. CabricÃ¯Â¿Â½n
- ◯ c. HuitÃ¯Â¿Â½n
- ◯ d. Sibilia
- ◯ e. CajolÃ¯Â¿Â½
- ◯ f. Palestina de los Altos
- ◯ g. San Miguel SigÃ¯Â¿Â½ila
- ◯ h. San Juan Ostuncalco
- ◯ i. San mateo
- ◯ j. San Francisco La UniÃ¯Â¿Â½n
- ◯ k. La Esperanza
- ◯ l. Olintepeque
- ◯ m. SalcajÃ¯Â¿Â½
- ◯ n. Quetzaltenango
- ◯ o. Almolonga
- ◯ p. Cantel
- ◯ q. Zunil
- ◯ r. El Palmar
- ◯ s. ConcepciÃ¯Â¿Â½n Chiquirichapa
- ◯ t. San Martin SacatepÃ¯Â¿Â½quez
- ◯ u. Colomba
- ◯ v. Flores Costa Cuca
- ◯ w. Coatepeque

29. En este viaje, Â¿CuÃ¡nto tiempo estuvo fuera de la casa el niÃ±o?

- ◯ a. Una semana o menos
- ◯ b. MÃ¡s de una semana, pero menos de un mes
- ◯ c. Un mes o mÃ¡s

30. Con que frecuencia viaja a esa localidad? (NÃƒÂºmero de veces)

____

30.1. Con que frecuencia viaja a esa localidad? (Veces por)

- ◯ Semana
- ◯ Mes
- ◯ AÃƒÂ±o

31. En los Ãºltimos 14 dÃas Â¿el niÃ±o ha ido a un departamento fuera de Quetzaltenango?

- ◯ a. Si
- ◯ b. No
- ◯ c. No sabe
- ◯ d. Se rehÃºsa a responder

32. A que departamento?

- ◯ a. Alta Verapaz
- ◯ b. Baja Verapaz
- ◯ c. Chimaltenango
- ◯ d. Chiquimula
- ◯ e. El PetÃ¯Â¿Â½n
- ◯ f. El Progreso
- ◯ g. El QuichÃ¯Â¿Â½
- ◯ h. Escuintla
- ◯ i. Guatemala
- ◯ j. Huehuetenango
- ◯ k. Izabal
- ◯ l. Jalapa
- ◯ m. Jutiapa
- ◯ n. Quetzaltenango
- ◯ o. Retalhuleu
- ◯ p. SacatepÃ¯Â¿Â½quez
- ◯ q. San Marcos
- ◯ r. Santa Rosa
- ◯ s. SololÃ¯Â¿Â½
- ◯ t. SuchitepÃ¯Â¿Â½quez
- ◯ u. TotonicapÃ¯Â¿Â½n
- ◯ v. Zacapa

33. En su ultimo viaje al departamento X, cuanto tiempo estuvo fuera de su casa?
- ◯ a. Una semana o menos
- ◯ b. MÃ¡s de una semana, pero menos de un mes
- ◯ c. Un mes o mÃ¡s

34. Con que frecuencia viaja a ese departamento? (NÃºmero de veces) __________________________________

34.1 Con que frecuencia viaja a ese departamento? (Veces por)
- ◯ Semana
- ◯ Mes
- ◯ AÃƒÂ±o

35. En estos 14 dÃ-as Â¿el niÃ±o ha ido a otro paÃ-s fuera de Guatemala?
- ◯ a. Si
- ◯ b. No

36. A que pais?
- ◯ a. MÃ¯Â¿Â½xico
- ◯ b. El Salvador
- ◯ c. Honduras
- ◯ d. Belice
- ◯ e. Estados Unidos
- ◯ f. Otro.

36.1 Â¿CuÃ¡l otro paÃ-s? __________________________________

37. En el Ãºltimo viaje del niÃ±o a otro paÃ-s, Â¿cuÃ¡nto tiempo estuvo fuera de su casa
- ◯ a. Una semana o menos
- ◯ b. MÃ¡s de una semana, pero menos de un mes
- ◯ c. Un mes o mÃ¡s

38. Â¿Con quÃ© frecuencia viaja el niÃ±o a esa paÃ-s? __________________________________

38.1 Con que frecuencia viaja el niÃ±o a ese paÃ-s? (Veces por)
- ◯ Semana
- ◯ Mes
- ◯ AÃ±o

IV.Historia de uso antibiÃ³tico

En la siguiente secciÃ³n de la encuesta le preguntaremos sobre el uso de medicamentos, especialmente, el uso de antibiÃ³ticos por el niÃ±o. Los antibiÃ³ticos se definen como medicamentos usados para matar las bacterias.

39. Â¿El niÃ±o ha tomado antibiÃ³ticos alguna vez?
- ◯ a. Si
- ◯ b. No
- ◯ c. No sabe
- ◯ d. Se rehÃºsa a responder

40. Â¿El niÃ±o ha tomado antibiÃ³ticos en los Ãºltimos 14 dÃ-as?
- ◯ a. Si
- ◯ b. No
- ◯ c. No sabe
- ◯ d. Se rehÃºsa a responder

41. La Ãºltima vez que el niÃ±o tomÃ³ un antibiÃ³tico, Â¿para quÃ© enfermedad lo tomÃ³? (puede seleccionar mÃ¡s de una)

- ☐ a. Nariz congestionada
- ☐ b. Tos
- ☐ c. Dolor de cabeza
- ☐ d. Fiebre
- ☐ e. VÃƒÂ³mitos
- ☐ f. Diarrea
- ☐ g. Heridas de la piel
- ☐ h. Malaria
- ☐ i. VIH
- ☐ j. Zika
- ☐ k. Dengue
- ☐ l. Chikungunya
- ☐ m. Tuberculosis
- ☐ n. Otro. Especificar.
- ☐ o. No Sabe
- ☐ p. Se rehÃºsa a responder

Otro ______________________________

42. Hablando de la enfermedad que le dio en la P41, Â¿BuscÃ³ algÃºn tipo de ayuda mÃ©dica para tratar la enfermedad del niÃ±o?

- ◯ a. SÃ-Â-
- ◯ b. No
- ◯ c. No sabe
- ◯ d. Se rehÃºsa a responder

43. Â¿DÃ³nde buscÃ³ ayuda mÃ©dica para el niÃ±o?

- ☐ a. Puesto de Salud
- ☐ b. Centro de Salud
- ☐ c. Hospital PÃºblico
- ☐ d. Hospital Privado
- ☐ e. ClÃ-nica Privada
- ☐ f. Centro de Convergencia
- ☐ g. Trabajador de Salud
- ☐ h. Feria MÃ©dica
- ☐ i. Farmacia
- ☐ j. Tienda
- ☐ k. Curandero
- ☐ l. Miembros de la Familia y/o amigos
- ☐ m. Otros, especificar
- ☐ n. No sabe
- ☐ o. Se rehÃºsa a responder

Otro ______________________________

44. Â¿QuÃ© medicamentos usÃ³ para tratar al niÃ±o en esta enfermedad en particular (pregunte para cada enfermedad mencionada en P42). Pida ver los medicamentos si estÃ¡n disponibles, y pregunte "Â¿quÃ© otro medicamento le dio al niÃ±o?" hasta que ya no mencionen otros medicamentos.

- ☐ a. AntibiÃ³ticos
- ☐ b. Desparasitantes
- ☐ c. Medicamentos para el dolor
- ☐ d. Medicamentos naturales
- ☐ e. Otros,
- ☐ f. No sabe
- ☐ g. Se rehÃºsa a responder

Otro ______________________________

Cuantos antibioticos ha tomado? ______________________________

20. Para cada antibiÃ³tico complete la siguiente tabla:

DATOS MEDICINA No 1
Nombre de la medicina (1) ______________________

â€¢ Se examino paquete o botella (si/no)
- ◯ Si
- ◯ No

â€¢ El nombre del medicamento se puede leer
- ◯ Si
- ◯ No

Le recetaron este medicamento en un puesto/centro/clinica privada de salud?
- ◯ Si
- ◯ No
- ◯ No se
- ◯ No Responde

â€¢ Si, indique en que lugar se le receto/recomendo.
- ◯ Centro de salud MSPAS
- ◯ Puesto de salud/ClÃnica privada

Si no, como sabia que medicamento comprar?
- ◯ Amigo o familiar
- ◯ Experiencia previa
- ◯ Persona en la farmacia/mÃ¯Â¿Â½dico/otro

En donde consiguio el medicamento?
- ◯ Farmacia
- ◯ Tienda
- ◯ Super
- ◯ MSPAS
- ◯ Amigo o familiar
- ◯ Comadronas
- ◯ Curanderos
- ◯ Otros

Cuantos dias tomo el medicamento? ______________________

Con que frecuencia toma antibioticos?
- ◯ Cada mes
- ◯ Cada tres meses
- ◯ Cada 6 meses
- ◯ Cada aÃ±o
- ◯ Cada vez que es necesario
- ◯ No sabe
- ◯ No responde

Ademas de usted, uso otra persona o animal este medicamento?
- ◯ SI
- ◯ No

â€¢ Si si, que otra persona o animal uso este medicamento?
- ☐ a. PapÃ¡ o MamÃ¡
- ☐ b, Pareja
- ☐ c. Hermanos(as)
- ☐ d. Hijos
- ☐ e. Abuelos
- ☐ f. Otro parientes
- ☐ g. Animal

Medicina 2

Nombre de la medicina ________________________________

â€¢ Se examino paquete o botella (si/no)
- ◯ Si
- ◯ No

â€¢ El nombre del medicamento se puede leer
- ◯ Si
- ◯ No

Le recetaron este medicamento en un puesto/centro/clinica privada de salud?
- ◯ Si
- ◯ No
- ◯ No se
- ◯ No Responde

â€¢ Si, indique en que lugar se le receto/recomendo.
- ◯ Centro de salud MSPAS
- ◯ Puesto de salud/ClÃnica privada

Si no, como sabia que medicamento comprar?
- ◯ Amigo o familiar
- ◯ Experiencia previa
- ◯ Persona en la farmacia/mÃ¯Â¿Â½dico/otro

En donde consiguio el medicamento?
- ◯ Farmacia
- ◯ Tienda
- ◯ Super
- ◯ MSPAS
- ◯ Amigo o familiar
- ◯ Comadronas
- ◯ Curanderos
- ◯ Otros

Cuantos dias tomo el medicamento? ________________________________

Con que frecuencia toma antibioticos?
- ◯ Cada mes
- ◯ Cada tres meses
- ◯ Cada 6 meses
- ◯ Cada aÃ±o
- ◯ Cada vez que es necesario
- ◯ No sabe
- ◯ No responde

Ademas de usted, uso otra persona o animal este medicamento?
- ◯ SI
- ◯ No

â€¢ Si si, que otra persona o animal uso este medicamento?
- ☐ a. PapÃi o MamÃi
- ☐ b, Pareja
- ☐ c. Hermanos(as)
- ☐ d. Hijos
- ☐ e. Abuelos
- ☐ f. Otro parientes
- ☐ g. Animal

Medicina 3

Nombre de la medicina ________________________

â€¢ Se examino paquete o botella (si/no)
- ◯ Si
- ◯ No

â€¢ El nombre del medicamento se puede leer
- ◯ Si
- ◯ No

Le recetaron este medicamento en un puesto/centro/clinica privada de salud?
- ◯ Si
- ◯ No
- ◯ No se
- ◯ No Responde

Indique en que lugar se le receto/recomendo.
- ◯ Centro de salud MSPAS
- ◯ Puesto de salud/ClÃ¯Â¿Â½nica privada

Si no, como sabia que medicamento comprar?
- ◯ Amigo o familiar
- ◯ Experiencia previa
- ◯ Persona en la farmacia/mÃ¯Â¿Â½dico/otro

En donde consiguio el medicamento?
- ◯ Farmacia
- ◯ Tienda
- ◯ Super
- ◯ MSPAS
- ◯ Amigo o familiar
- ◯ Comadronas
- ◯ Curanderos
- ◯ Otros

Cuantos dias tomo el medicamento? ________________________

Con que frecuencia toma antibioticos?
- ◯ Cada mes
- ◯ Cada tres meses
- ◯ Cada 6 meses
- ◯ Cada aÃ±o
- ◯ Cada vez que es necesario
- ◯ No sabe
- ◯ No responde

Ademas de usted, uso otra persona o animal este medicamento?
- ◯ SI
- ◯ No

â€¢ Si si, que otra persona o animal uso este medicamento?
- ☐ a. PapÃi o MamÃi
- ☐ b, Pareja
- ☐ c. Hermanos(as)
- ☐ d. Hijos
- ☐ e. Abuelos
- ☐ f. Otro parientes
- ☐ g. Animal

V.Enfermedades Previas-Diarrea y exposiciÃ³n a hospitales: Ahora quisiera preguntarle sobre eventos previos de enfermedad diarreica del niÃ±o.

46. En los Ãºltimos 14 dÃ-as, Â¿ha tenido diarrea el niÃ±o?
- a. Si
- b. No
- c. No sabe
- d. Se rehÃºsa a responder

47. Â¿Hace cuÃ¡ntos dÃ-as tuvo el niÃ±o el Ãºltimo episodio de diarrea/asientos? NÃºmeros enteros

____

48. Durante el peor dÃ-a de diarrea/asientos: (EN UN PERÃODO DE 24 HORAS) Â¿cuÃ¡ntas veces fue al baÃ±o?

____

49. Durante este episodio de diarrea/asientos, Â¿tuvo el niÃ±o vÃ³mitos?
- a. Si
- b. No
- c. No sabe
- d. Se rehÃºsa a responder

50. Â¿BuscÃ³ algÃºn tipo de atenciÃ³n mÃ©dica fuera de la casa para curar la diarrea del niÃ±o?
- a. Si
- b. No
- c. No sabe
- d. Se rehÃºsa a responder

51. Si buscÃ³ algÃºn tipo de atenciÃ³n fuera de la casa, Â¿a dÃ³nde acudiÃ³? (marque todos los que apliquen)
- a. Puesto de Salud
- b. Centro de Salud
- c. Hospital PÃ¯Â¿Â½blico
- d. Hospital Privado
- e. ClÃ¯Â¿Â½nica Privada
- f. Centro de Convergencia
- g. Trabajador de Salud
- h. Feria MÃ¯Â¿Â½dica
- i. Farmacia
- j. Tienda
- k. Curandero
- l. Miembros de la Familia y/o amigos
- m.Otros

Otro __________________________________

52. Â¿El niÃ±o tomÃ³ algÃºn medicamento para tratar este episodio de diarrea/asientos?
- a. Si
- b. No
- c. No sabe
- d. Se rehÃºsa a responder

53. Â¿QuÃ© medicamentos tomÃ³ el niÃ±o?
- [ ] a. Medicamentos para el dolor
- [ ] b. Desparasitantes
- [ ] c. Remedios de hierbas
- [ ] d. Otros
- [ ] e. Ninguno

Otro __________________________________

Remedios de hierbas: __________________________________

54. Â¿Nos mencionÃ³ usted estos antibiÃ³ticos en las preguntas que le hicimos anteriormente (P45)?
- a. Si
- b. No
- c. No sabe
- d. Se rehÃºsa a responder

55. En los Ãºltimos 3 meses, Â¿el niÃ±o ha visitado el hospital por alguna razÃ³n?

- ◯ a. Si
- ◯ b. No
- ◯ c. No sabe
- ◯ d. RehÃºsa

56. Â¿Por quÃ© visitÃ³ el niÃ±o el hospital?

- ◯ a. Para hacer un tramite
- ◯ b. para visitar a un paciente internado/AcompaÃ±ar a un familiar
- ◯ c. Para un chequeo mÃ©dico rutinario
- ◯ d. Para recibir un tratamiento
- ◯ e. Porque estaba enfermo y lo internaron
- ◯ f. Otro
- ◯ g. No sabe
- ◯ h. Se rehÃºsa a responder

Otro ______________________________

57. En los Ãºltimos 3 meses, Â¿el niÃ±o ha visitado el centro de salud por alguna razÃ³n?

- ◯ a. Si
- ◯ b. No
- ◯ c. No sabe
- ◯ d. Se rehÃºsa a responder

58. Â¿Por quÃ© visitÃ³ el niÃ±o el centro de salud?

- ◯ a. Para hacer un tramite
- ◯ b. para visitar a un paciente internado/AcompaÃ±ar a un familiar
- ◯ c. Para un chequeo mÃ©dico rutinario
- ◯ d. para recibir un tratamiento
- ◯ e. porque estaba enferma y me internaron
- ◯ f. Otro, especificar
- ◯ g. No sabe
- ◯ h. Se rehÃºsa a responder

Otro ______________________________

Muestras de laboratorio Adultos

CÃ³digo ________________________________

Lugar de origen
◯ La Union
◯ El jardin
◯ San Juan Ostuncalco
◯ Monrovia

Muestra de Heces
◯ Si
◯ No

Fecha y hora de recolecciÃ³n (Trabajador de Campo) ________________________________

Fecha y hora de recepciÃ³n en sitio de Hospital ________________________________

Procesamiento en laboratorio

Heces entera
◯ Si
◯ No

Fecha de elaboraciÃ³n ________________________________

RecepciÃ³n Laboratorio UVG ________________________________

Heces liquida almacenada en crio tubo
◯ Si
◯ No

Fecha de elaboraciÃ³n ________________________________

RecepciÃ³n Laboratorio UVG ________________________________

Placa MacConkey para aislamiento de E. coli
◯ Si
◯ No

Fecha de elaboraciÃ³n ________________________________

RecepciÃ³n Laboratorio UVG ________________________________

Placa de 96 pocillos con caldo LB
◯ Si
◯ No

Fecha de elaboraciÃ³n ________________________________

RecepciÃ³n Laboratorio UVG ________________________________

Placas MacConkey con antibiÃ³ticos
◯ Si
◯ No

Fecha de elaboraciÃ³n ________________________________

Fecha de elaboraciÃ³n ________________________________

Pruebas de parÃisitos

Â¿Se realizo la prueba de parÃisitos?
- ◯ Si
- ◯ No

Fecha en que se realizo la prueba de parÃisitos ______________________

Â¿Resultado de prueba de parÃisitos?
- ◯ Helmintos
- ◯ Protozoos
- ◯ Ambos
- ◯ Negativo

Observaciones ______________________

Muestras de laboratorio Menores de 5 AÃ±os

CÃ³digo ______________________

Lugar de origen
- ◯ La Union
- ◯ El jardin
- ◯ San Juan Ostuncalco
- ◯ Monrovia

Muestra de Heces
- ◯ Si
- ◯ No

Fecha y hora de recolecciÃ³n (Trabajador de Campo) ______________________

Fecha y hora de recepciÃ³n en sitio de Hospital ______________________

Procesamiento en laboratorio

Heces entera
- ◯ Si
- ◯ No

Fecha de elaboraciÃ³n ______________________

RecepciÃ³n Laboratorio UVG ______________________

Heces liquida almacenada en crio tubo
- ◯ Si
- ◯ No

Fecha de elaboraciÃ³n ______________________

RecepciÃ³n Laboratorio UVG ______________________

Placa MacConkey para aislamiento de E. coli
- ◯ Si
- ◯ No

Fecha de elaboraciÃ³n ______________________

RecepciÃ³n Laboratorio UVG ______________________

Placa de 96 pocillos con caldo LB
- ◯ Si
- ◯ No

Fecha de elaboraciÃ³n ______________________

RecepciÃ³n Laboratorio UVG ______________________

Placas MacConkey con antibiÃ³ticos
- ◯ Si
- ◯ No

Fecha de elaboraciÃ³n ______________________

Fecha de elaboraciÃ³n ______________________

Pruebas de parÃisitos

Â¿Se realizo la prueba de parÃisitos?
- ◯ Si
- ◯ No

Fecha en que se realizo la prueba de parÃisitos ________________________________

Â¿Resultado de prueba de parÃisitos?
- ◯ Helmintos
- ◯ Protozoos
- ◯ Ambos
- ◯ Negativo

Observaciones ________________________________

Cuestionario De Hogar

Record ID ________________________________

Fecha en que se realiza la encuesta ________________________________

I. Buenos d&iacuteas/tardes, mi nombre es (NOMBRE PERSONA HABLANDO) y vengo hoy con mi compa&ntildeero de trabajo (NOMBRE COMPA&NtildeERO DEL TRABAJO). Estamos visitando su casa el d&iacutea de hoy porque estamos realizando la segunda fase del estudio sobre la resistencia a los antibi&oacuteticos. &iquestSer&iacutea posible hablar con ______________ (nombrar al representante del hogar que entrevistamos anteriormente)?

- ◯ a. Si- continuar
- ◯ b. No, no est&aacute el representante del hogar. Preguntar a qu&eacute hora pueden regresar para hablar con la persona que particip&oacute anteriormente.
- ◯ c.Persona se niega a participar en la segunda fase- terminar.

1. &iquestCu&aacutentas personas en total viven en esta vivienda (incluir ni&ntildeos, ancianos, personas discapacitadas, etc.)? ________________________________

Genero(1):
- ◯ 1. Masculino
- ◯ 2. Femenino

Edad(1): ________________________________

Genero(2):
- ◯ 1. Masculino
- ◯ 2. Femenino

Edad(2): ________________________________

Genero(3):
- ◯ 1. Masculino
- ◯ 2. Femenino

Edad(3): ________________________________

Genero(4):
- ◯ 1. Masculino
- ◯ 2. Femenino

Edad(4): ________________________________

Genero(5):
- ◯ 1. Masculino
- ◯ 2. Femenino

Edad(5): ________________________________

Genero(6):
- ◯ 1. Masculino
- ◯ 2. Femenino

Edad(6): ________________________________

Genero(7):
- ◯ 1. Masculino
- ◯ 2. Femenino

Edad(7): ________________________________

Genero(8):
- ◯ 1. Masculino
- ◯ 2. Femenino

Edad(8): ________________________________

Genero(9):
- ◯ 1. Masculino
- ◯ 2. Femenino

Edad(9): ______________________

Genero(10):
- ◯ 1. Masculino
- ◯ 2. Femenino

Edad(10): ______________________

Genero(11):
- ◯ 1. Masculino
- ◯ 2. Femenino

Edad(11): ______________________

Genero(12):
- ◯ 1. Masculino
- ◯ 2. Femenino

Edad(12): ______________________

Genero(13):
- ◯ 1. Masculino
- ◯ 2. Femenino

Edad(13): ______________________

Genero(14):
- ◯ 1. Masculino
- ◯ 2. Femenino

Edad(14): ______________________

Genero(15):
- ◯ 1. Masculino
- ◯ 2. Femenino

Edad(15): ______________________

Genero(16):
- ◯ 1. Masculino
- ◯ 2. Femenino

Edad(16): ______________________

Genero(17):
- ◯ 1. Masculino
- ◯ 2. Femenino

Edad(17): ______________________

Genero(18):
- ◯ 1. Masculino
- ◯ 2. Femenino

Edad(18): ______________________

Genero(19):
- ◯ 1. Masculino
- ◯ 2. Femenino

Edad(19): ______________________

Genero(20):
- ◯ 1. Masculino
- ◯ 2. Femenino

Edad(20): ______________________

INFORMACIoN DEMOGR&AacuteFICA del representante del hogar

1. C&oacutedigo / identificaci&oacuten ______________________

2. Localidad
- ◯ Coatepeque
- ◯ San Juan Ostuncalco

3. Coatepeque

◯ 1.El Jardin
◯ 2.La Union

3. San Juan Ostuncalco

◯ 1.Casco Urbano
◯ 2.Monrovia

4. &iquestTiene usted en su vivienda Electricidad?

◯ Si
◯ No
◯ No sabe
◯ Se reh&uacutesa a responder

5.&iquestTiene usted en su vivienda Computadora?

◯ Si
◯ No
◯ No sabe
◯ Se reh&uacutesa a responder

Secci&oacuten 2 ENCUESTADOR LEA: Muchas gracias por su tiempo. Ahora quiero hacerle unas preguntas sobre varias condiciones en su hogar. Estas preguntas las hacemos en cada casa que visitamos.

6. Los miembros de su familia tienen animales que viven libremente afuera de la casa? (MARQUE UNO)

◯ Si
◯ No
◯ No sabe
◯ Se reh&uacutesa a responder

7. Que animales tienen? (MARQUE TODAS LAS QUE APLIQUEN, INDICAR CUANTOS ANIMALES DE CADA TIPO)

☐ a. Perros
☐ b. Gatos
☐ c. Pollos
☐ d. Pavos
☐ e. Patos
☐ f. Gansos
☐ g. Otros p&aacutejaros
☐ h. Cerdos
☐ i. Vacas
☐ j. Ovejas
☐ k. Cabras
☐ l. Otros animales
☐ m. No sabe
☐ n. Se reh&uacutesa a responder

Cual otro animal? ________________________________

Qu&eacute otro p&aacutejaro? ________________________________

En la siguiente secci&oacuten de la encuesta le preguntaremos sobre el uso de medicamentos, especialmente, el uso de antibi&oacuteticos con sus animales. Los antibi&oacuteticos se definen como medicamentos usados para matar las bacterias. Las bacterias son microorganismos que causan infecciones. Los antibi&oacuteticos se venden en las farmacias y agropecuarias de Guatemala y algunas veces en tiendas y supermercados. El Ministerio de Salud P&uacuteblica tambi&eacuten proporciona antibi&oacuteticos a los que tienen una receta de un m&eacutedico del MSPAS. Los antibi&oacuteticos m&aacutes comunes son amoxicilina y tetraciclina (mostrar ejemplos adquiridos del ministerio de salud y tiendas cercanas) y frecuentemente se recomiendan despu&eacutes de visitar al doctor. Algunas veces, las personas deciden tomar antibi&oacuteticos sin tener la recomendaci&oacuten del doctor o veterinario.

PERROS
Cuantos Perros? ________________________________

Desde la &uacuteltima visita, &iquestles ha dado antibi&oacuteticos a su/sus perro/perros?

- ◯ a. Si
- ◯ b. No
- ◯ c. No sabe
- ◯ d. Se reh&uacutesa a responder

DATOS MEDICINA PERROS
Nombre de la Medicina/Antibiotico ______________________________

Caja de la medicina o botella vista (si/no)

- ◯ Si
- ◯ No

Se puede leer el nombre?

- ◯ Si
- ◯ No

&iquestCon qu&eacute frecuencia les da esta medicina a sus animales?

- ◯ Cada mes
- ◯ Cada tres meses
- ◯ Cada 6 meses
- ◯ Cada a&ntildeo
- ◯ Cada vez que es necesario
- ◯ No sabe
- ◯ No responde

GATOS
Cuantos Gatos? ______________________________

Desde la &uacuteltima visita, &iquestles ha dado antibi&oacuteticos a su/sus gato/gatos?

- ◯ a. Si
- ◯ b. No
- ◯ c. No sabe
- ◯ d. Se reh&uacutesa a responder

DATOS MEDICINA GATOS
Nombre de la Medicina/Antibiotico ______________________________

Caja de la medicina o botella vista (si/no)

- ◯ Si
- ◯ No

Se puede leer el nombre?

- ◯ Si
- ◯ No

&iquestCon qu&eacute frecuencia les da esta medicina a sus animales?

- ◯ Cada mes
- ◯ Cada tres meses
- ◯ Cada 6 meses
- ◯ Cada a&ntildeo
- ◯ Cada vez que es necesario
- ◯ No sabe
- ◯ No responde

PAVOS
Cuantos Pavos? ______________________________

Desde la &uacuteltima visita, &iquestles ha dado antibi&oacuteticos a su/sus pavo/pavos?

- ◯ a. Si
- ◯ b. No
- ◯ c. No sabe
- ◯ d. Se reh&uacutesa a responder

DATOS MEDICINA PAVOS
Nombre de la Medicina/Antibiotico ______________________________

Caja de la medicina o botella vista (si/no)

- ◯ Si
- ◯ No

Se puede leer el nombre?

- ◯ Si
- ◯ No

&iquestCon qu&eacute frecuencia les da esta medicina a sus animales?

- ◯ Cada mes
- ◯ Cada tres meses
- ◯ Cada 6 meses
- ◯ Cada a&ntildeo
- ◯ Cada vez que es necesario
- ◯ No sabe
- ◯ No responde

Despu&eacutes de darle antibi&oacuteticos, &iquestha dejado de tomar la leche, comer los huevos o comer la carne del animal indicado, despu&eacutes de darle antibi&oacuteticos?

- ◯ SI
- ◯ No

&iquestDurante cuanto tiempo ha dejado de tomarla? (Numero de d&iacuteas) ______________________________

POLLOS
Cuantos Pollos? ______________________________

Desde la &uacuteltima visita, &iquestles ha dado antibi&oacuteticos a su/sus pollo/pollos?

- ◯ a. Si
- ◯ b. No
- ◯ c. No sabe
- ◯ d. Se reh&uacutesa a responder

DATOS MEDICINA POLLOS
Nombre de la Medicina/Antibiotico ______________________________

Caja de la medicina o botella vista (si/no)

- ◯ Si
- ◯ No

Se puede leer el nombre?

- ◯ Si
- ◯ No

&iquestCon qu&eacute frecuencia les da esta medicina a sus animales?

- ◯ Cada mes
- ◯ Cada tres meses
- ◯ Cada 6 meses
- ◯ Cada a&ntildeo
- ◯ Cada vez que es necesario
- ◯ No sabe
- ◯ No responde

Despu&eacutes de darle antibi&oacuteticos, &iquestha dejado de tomar la leche, comer los huevos o comer la carne del animal indicado, despu&eacutes de darle antibi&oacuteticos?

- ◯ SI
- ◯ No

&iquestDurante cuanto tiempo ha dejado de tomarla? (Numero de d&iacuteas) ______________________________

PATOS
Cuantos Patos? ______________________________

Desde la &uacuteltima visita, &iquestles ha dado antibi&oacuteticos a su/sus patos/patos?

- ◯ a. Si
- ◯ b. No
- ◯ c. No sabe
- ◯ d. Se reh&uacutesa a responder

DATOS MEDICINA PATOS
Nombre de la Medicina/Antibiotico ______________________

Caja de la medicina o botella vista (si/no)
- ◯ Si
- ◯ No

Se puede leer el nombre?
- ◯ Si
- ◯ No

&iquestCon qu&eacute frecuencia les da esta medicina a sus animales?
- ◯ Cada mes
- ◯ Cada tres meses
- ◯ Cada 6 meses
- ◯ Cada a&ntildeo
- ◯ Cada vez que es necesario
- ◯ No sabe
- ◯ No responde

Despu&eacutes de darle antibi&oacuteticos, &iquestha dejado de tomar la leche, comer los huevos o comer la carne del animal indicado, despu&eacutes de darle antibi&oacuteticos?
- ◯ SI
- ◯ No

&iquestDurante cuanto tiempo ha dejado de tomarla? (Numero de d&iacuteas) ______________________

GANSO
Cuantos Ganso? ______________________

Desde la &uacuteltima visita, &iquestles ha dado antibi&oacuteticos a su/sus ganso/gansos?
- ◯ a. Si
- ◯ b. No
- ◯ c. No sabe
- ◯ d. Se reh&uacutesa a responder

DATOS MEDICINA GANSOS
Nombre de la Medicina/Antibiotico ______________________

Caja de la medicina o botella vista (si/no)
- ◯ Si
- ◯ No

Se puede leer el nombre?
- ◯ Si
- ◯ No

&iquestCon qu&eacute frecuencia les da esta medicina a sus animales?
- ◯ Cada mes
- ◯ Cada tres meses
- ◯ Cada 6 meses
- ◯ Cada a&ntildeo
- ◯ Cada vez que es necesario
- ◯ No sabe
- ◯ No responde

Despu&eacutes de darle antibi&oacuteticos, &iquestha dejado de tomar la leche, comer los huevos o comer la carne del animal indicado, despu&eacutes de darle antibi&oacuteticos?
- ◯ SI
- ◯ No

&iquestDurante cuanto tiempo ha dejado de tomarla? (Numero de d&iacuteas) ______________________

PAJAROS
Cuantos Pajaros? __________________________________

Desde la &uacuteltima visita, &iquestles ha dado antibi&oacuteticos a su/sus pajaro/pajaros?
- ◯ a. Si
- ◯ b. No
- ◯ c. No sabe
- ◯ d. Se reh&uacutesa a responder

DATOS MEDICINA PAJAROS
Nombre de la Medicina/Antibiotico __________________________________

Caja de la medicina o botella vista (si/no)
- ◯ Si
- ◯ No

Se puede leer el nombre?
- ◯ Si
- ◯ No

&iquestCon qu&eacute frecuencia les da esta medicina a sus animales?
- ◯ Cada mes
- ◯ Cada tres meses
- ◯ Cada 6 meses
- ◯ Cada a&ntildeo
- ◯ Cada vez que es necesario
- ◯ No sabe
- ◯ No responde

Despu&eacutes de darle antibi&oacuteticos, &iquestha dejado de tomar la leche, comer los huevos o comer la carne del animal indicado, despu&eacutes de darle antibi&oacuteticos?
- ◯ SI
- ◯ No

&iquestDurante cuanto tiempo ha dejado de tomarla? (Numero de d&iacuteas) __________________________________

CERDOS
Cuantos Cerdos? __________________________________

Desde la &uacuteltima visita, &iquestles ha dado antibi&oacuteticos a su/sus cerdo/cerdos?
- ◯ a. Si
- ◯ b. No
- ◯ c. No sabe
- ◯ d. Se reh&uacutesa a responder

DATOS MEDICINA CERDOS
Nombre de la Medicina/Antibiotico __________________________________

Caja de la medicina o botella vista (si/no)
- ◯ Si
- ◯ No

Se puede leer el nombre?
- ◯ Si
- ◯ No

&iquestCon qu&eacute frecuencia les da esta medicina a sus animales?
- ◯ Cada mes
- ◯ Cada tres meses
- ◯ Cada 6 meses
- ◯ Cada a&ntildeo
- ◯ Cada vez que es necesario
- ◯ No sabe
- ◯ No responde

Despu&eacutes de darle antibi&oacuteticos, &iquestha dejado de comer la carne del animal indicado, despu&eacutes de darle antibi&oacuteticos?	◯ SI<br>◯ No
&iquestDurante cuanto tiempo ha dejado de tomarla? (Numero de d&iacuteas)	______________________
VACAS<br>Cuantos Vacas?	______________________
Desde la &uacuteltima visita, &iquestles ha dado antibi&oacuteticos a su/sus vaca/vacas?	◯ a. Si<br>◯ b. No<br>◯ c. No sabe<br>◯ d. Se reh&uacutesa a responder
DATOS MEDICINA VACAS<br>Nombre de la Medicina/Antibiotico	______________________
Caja de la medicina o botella vista (si/no)	◯ Si<br>◯ No
Se puede leer el nombre?	◯ Si<br>◯ No
&iquestCon qu&eacute frecuencia les da esta medicina a sus animales?	◯ Cada mes<br>◯ Cada tres meses<br>◯ Cada 6 meses<br>◯ Cada a&ntildeo<br>◯ Cada vez que es necesario<br>◯ No sabe<br>◯ No responde
Despu&eacutes de darle antibi&oacuteticos, &iquestha dejado de tomar la leche, comer la carne del animal indicado, despu&eacutes de darle antibi&oacuteticos?	◯ SI<br>◯ No
&iquestDurante cuanto tiempo ha dejado de tomarla? (Numero de d&iacuteas)	______________________
OVEJAS<br>Cuantos Ovejas?	______________________
Desde la &uacuteltima visita, &iquestles ha dado antibi&oacuteticos a su/sus oveja/ovejas?	◯ a. Si<br>◯ b. No<br>◯ c. No sabe<br>◯ d. Se reh&uacutesa a responder
DATOS MEDICINA OVEJAS<br>Nombre de la Medicina/Antibiotico	______________________
Caja de la medicina o botella vista (si/no)	◯ Si<br>◯ No
Se puede leer el nombre?	◯ Si<br>◯ No

&iquestCon qu&eacute frecuencia les da esta medicina a sus animales?

- ◯ Cada mes
- ◯ Cada tres meses
- ◯ Cada 6 meses
- ◯ Cada a&ntildeo
- ◯ Cada vez que es necesario
- ◯ No sabe
- ◯ No responde

Despu&eacutes de darle antibi&oacuteticos, &iquestha dejado de tomar la leche o comer la carne del animal indicado, despu&eacutes de darle antibi&oacuteticos?

- ◯ SI
- ◯ No

&iquestDurante cuanto tiempo ha dejado de tomarla? (Numero de d&iacuteas) ________________________________

CABRAS
Cuantos Cabras? ________________________________

Desde la &uacuteltima visita, &iquestles ha dado antibi&oacuteticos a su/sus cabra/cabras?

- ◯ a. Si
- ◯ b. No
- ◯ c. No sabe
- ◯ d. Se reh&uacutesa a responder

DATOS MEDICINA CABRAS
Nombre de la Medicina/Antibiotico ________________________________

Caja de la medicina o botella vista (si/no)

- ◯ Si
- ◯ No

Se puede leer el nombre?

- ◯ Si
- ◯ No

&iquestCon qu&eacute frecuencia les da esta medicina a sus animales?

- ◯ Cada mes
- ◯ Cada tres meses
- ◯ Cada 6 meses
- ◯ Cada a&ntildeo
- ◯ Cada vez que es necesario
- ◯ No sabe
- ◯ No responde

Despu&eacutes de darle antibi&oacuteticos, &iquestha dejado de tomar la leche o comer la carne del animal indicado, despu&eacutes de darle antibi&oacuteticos?

- ◯ SI
- ◯ No

&iquestDurante cuanto tiempo ha dejado de tomarla? (Numero de d&iacuteas) ________________________________

OTROS ANIMALES
Cuantos Otros Animales? ________________________________

Desde la &uacuteltima visita, &iquestles ha dado antibi&oacuteticos a su/sus otro animales?

- ◯ a. Si
- ◯ b. No
- ◯ c. No sabe
- ◯ d. Se reh&uacutesa a responder

DATOS MEDICINA OTROS ANIMALES
Nombre de la Medicina/Antibiotico ________________________________

Caja de la medicina o botella vista (si/no)

- ◯ Si
- ◯ No

Se puede leer el nombre?

- ◯ Si
- ◯ No

&iquestCon qu&eacute frecuencia les da esta medicina a sus animales?

- ◯ Cada mes
- ◯ Cada tres meses
- ◯ Cada 6 meses
- ◯ Cada a&ntildeo
- ◯ Cada vez que es necesario
- ◯ No sabe
- ◯ No responde

Despu&eacutes de darle antibi&oacuteticos, &iquestha dejado de tomar la leche o comer la carne del animal indicado, despu&eacutes de darle antibi&oacuteticos?

- ◯ SI
- ◯ No

&iquestDurante cuanto tiempo ha dejado de tomarla? (Numero de d&iacuteas)

____

10. Tiene alg&uacuten animal que vive adentro de la casa?

- ◯ a. Si
- ◯ b. No

11. Por favor indique el tipo y numero de animales que viven libremente adentro de la casa. (MARQUE TODOS LOS QUE APLIQUEN indicando cantidad)

- ☐ a. Perros
- ☐ b. Gatos
- ☐ c. Pollos
- ☐ d. Pavos
- ☐ e. Patos
- ☐ f. Gansos
- ☐ g. Otros p&aacutejaros
- ☐ h. Cerdos
- ☐ i. Vacas
- ☐ j. Ovejas
- ☐ k. Cabras
- ☐ l. Otros animales
- ☐ m. No sabe
- ☐ n. Se reh&uacutesa a responder

Cual otro animal?

____

PERROS
Cuantos Perros?

____

&iquestEn los &uacuteltimos 6 meses les ha dado antibi&oacuteticos a su/sus perro/perros?

- ◯ a. Si
- ◯ b. No
- ◯ c. No sabe
- ◯ d. Se reh&uacutesa a responder

DATOS MEDICINA PERROS
Nombre de la Medicina/Antibiotico

____

Caja de la medicina o botella vista (si/no)

- ◯ Si
- ◯ No

Se puede leer el nombre?

- ◯ Si
- ◯ No

&iquestCon qu&eacute frecuencia les da esta medicina a sus animales?

- ◯ Cada mes
- ◯ Cada tres meses
- ◯ Cada 6 meses
- ◯ Cada a&ntildeo
- ◯ Cada vez que es necesario
- ◯ No sabe
- ◯ No responde

&iquestEn los &uacuteltimos 6 meses ha dejado de tomar la leche, comer los huevos o comer la carne del animal indicado, despu&eacutes de darle antibi&oacuteticos?

- ◯ SI
- ◯ No

&iquestDurante cuanto tiempo ha dejado de tomarla? (Numero de d&iacuteas) ______________________________

GATOS
Cuantos Gatos? ______________________________

&iquestEn los &uacuteltimos 6 meses les ha dado antibi&oacuteticos a su/sus gato/gatos?

- ◯ a. Si
- ◯ b. No
- ◯ c. No sabe
- ◯ d. Se reh&uacutesa a responder

DATOS MEDICINA GATOS
Nombre de la Medicina/Antibiotico ______________________________

Caja de la medicina o botella vista (si/no)

- ◯ Si
- ◯ No

&iquestCon qu&eacute frecuencia les da esta medicina a sus animales?

- ◯ Cada mes
- ◯ Cada tres meses
- ◯ Cada 6 meses
- ◯ Cada a&ntildeo
- ◯ Cada vez que es necesario
- ◯ No sabe
- ◯ No responde

Se puede leer el nombre?

- ◯ Si
- ◯ No

&iquestEn los &uacuteltimos 6 meses ha dejado de tomar la leche, comer los huevos o comer la carne del animal indicado, despu&eacutes de darle antibi&oacuteticos?

- ◯ SI
- ◯ No

&iquestDurante cuanto tiempo ha dejado de tomarla? (Numero de d&iacuteas) ______________________________

PAVOS
Cuantos Pavos? ______________________________

&iquestEn los &uacuteltimos 6 meses les ha dado antibi&oacuteticos a su/sus pavo/pavos?

- ◯ a. Si
- ◯ b. No
- ◯ c. No sabe
- ◯ d. Se reh&uacutesa a responder

DATOS MEDICINA PAVOS
Nombre de la Medicina/Antibiotico ________________________________

Caja de la medicina o botella vista (si/no)
- ◯ Si
- ◯ No

Se puede leer el nombre?
- ◯ Si
- ◯ No

&iquestCon qu&eacute frecuencia les da esta medicina a sus animales?
- ◯ Cada mes
- ◯ Cada tres meses
- ◯ Cada 6 meses
- ◯ Cada a&ntildeo
- ◯ Cada vez que es necesario
- ◯ No sabe
- ◯ No responde

&iquestEn los &uacuteltimos 6 meses ha dejado de tomar la leche, comer los huevos o comer la carne del animal indicado, despu&eacutes de darle antibi&oacuteticos?
- ◯ SI
- ◯ No

&iquestDurante cuanto tiempo ha dejado de tomarla? (Numero de d&iacuteas) ________________________________

POLLOS
Cuantos Pollos? ________________________________

&iquestEn los &uacuteltimos 6 meses les ha dado antibi&oacuteticos a su/sus pollo/pollos?
- ◯ a. Si
- ◯ b. No
- ◯ c. No sabe
- ◯ d. Se reh&uacutesa a responder

DATOS MEDICINA POLLOS
Nombre de la Medicina/Antibiotico ________________________________

Caja de la medicina o botella vista (si/no)
- ◯ Si
- ◯ No

Se puede leer el nombre?
- ◯ Si
- ◯ No

&iquestCon qu&eacute frecuencia les da esta medicina a sus animales?
- ◯ Cada mes
- ◯ Cada tres meses
- ◯ Cada 6 meses
- ◯ Cada a&ntildeo
- ◯ Cada vez que es necesario
- ◯ No sabe
- ◯ No responde

&iquestEn los &uacuteltimos 6 meses ha dejado de tomar la leche, comer los huevos o comer la carne del animal indicado, despu&eacutes de darle antibi&oacuteticos?
- ◯ SI
- ◯ No

&iquestDurante cuanto tiempo ha dejado de tomarla? (Numero de d&iacuteas) ______________________

PATOS
Cuantos Patos? ______________________

&iquestEn los &uacuteltimos 6 meses les ha dado antibi&oacuteticos a su/sus patos/patos?
- a. Si
- b. No
- c. No sabe
- d. Se reh&uacutesa a responder

DATOS MEDICINA PATOS
Nombre de la Medicina/Antibiotico ______________________

Caja de la medicina o botella vista (si/no)
- Si
- No

Se puede leer el nombre?
- Si
- No

&iquestCon qu&eacute frecuencia les da esta medicina a sus animales?
- Cada mes
- Cada tres meses
- Cada 6 meses
- Cada a&ntildeo
- Cada vez que es necesario
- No sabe
- No responde

&iquestEn los &uacuteltimos 6 meses ha dejado de tomar la leche, comer los huevos o comer la carne del animal indicado, despu&eacutes de darle antibi&oacuteticos?
- SI
- No

&iquestDurante cuanto tiempo ha dejado de tomarla? (Numero de d&iacuteas) ______________________

GANSO
Cuantos Ganso? ______________________

&iquestEn los &uacuteltimos 6 meses les ha dado antibi&oacuteticos a su/sus ganso/gansos?
- a. Si
- b. No
- c. No sabe
- d. Se reh&uacutesa a responder

DATOS MEDICINA GANSOS
Nombre de la Medicina/Antibiotico ______________________

Caja de la medicina o botella vista (si/no)
- Si
- No

Se puede leer el nombre?
- Si
- No

&iquestCon qu&eacute frecuencia les da esta medicina a sus animales?

- ◯ Cada mes
- ◯ Cada tres meses
- ◯ Cada 6 meses
- ◯ Cada a&ntildeo
- ◯ Cada vez que es necesario
- ◯ No sabe
- ◯ No responde

&iquestEn los &uacuteltimos 6 meses ha dejado de tomar la leche, comer los huevos o comer la carne del animal indicado, despu&eacutes de darle antibi&oacuteticos?

- ◯ SI
- ◯ No

&iquestDurante cuanto tiempo ha dejado de tomarla? (Numero de d&iacuteas) __________________________________

PAJAROS
Cuantos Pajaros? __________________________________

&iquestEn los &uacuteltimos 6 meses les ha dado antibi&oacuteticos a su/sus pajaro/pajaros?

- ◯ a. Si
- ◯ b. No
- ◯ c. No sabe
- ◯ d. Se reh&uacutesa a responder

DATOS MEDICINA PAJAROS
Nombre de la Medicina/Antibiotico __________________________________

Caja de la medicina o botella vista (si/no)

- ◯ Si
- ◯ No

Se puede leer el nombre?

- ◯ Si
- ◯ No

&iquestCon qu&eacute frecuencia les da esta medicina a sus animales?

- ◯ Cada mes
- ◯ Cada tres meses
- ◯ Cada 6 meses
- ◯ Cada a&ntildeo
- ◯ Cada vez que es necesario
- ◯ No sabe
- ◯ No responde

&iquestEn los &uacuteltimos 6 meses ha dejado de tomar la leche, comer los huevos o comer la carne del animal indicado, despu&eacutes de darle antibi&oacuteticos?

- ◯ SI
- ◯ No

&iquestDurante cuanto tiempo ha dejado de tomarla? (Numero de d&iacuteas) __________________________________

&iquestEn los &uacuteltimos 6 meses les ha dado antibi&oacuteticos a su/sus cerdo/cerdos?

- ◯ a. Si
- ◯ b. No
- ◯ c. No sabe
- ◯ d. Se reh&uacutesa a responder

CERDOS
Cuantos Cerdos? __________________________________

DATOS MEDICINA CERDOS
Nombre de la Medicina/Antibiotico ________________________________

Caja de la medicina o botella vista (si/no)
- ◯ Si
- ◯ No

Se puede leer el nombre?
- ◯ Si
- ◯ No

&iquestCon qu&eacute frecuencia les da esta medicina a sus animales?
- ◯ Cada mes
- ◯ Cada tres meses
- ◯ Cada 6 meses
- ◯ Cada a&ntildeo
- ◯ Cada vez que es necesario
- ◯ No sabe
- ◯ No responde

&iquestEn los &uacuteltimos 6 meses ha dejado de tomar la leche, comer los huevos o comer la carne del animal indicado, despu&eacutes de darle antibi&oacuteticos?
- ◯ SI
- ◯ No

&iquestDurante cuanto tiempo ha dejado de tomarla? (Numero de d&iacuteas) ________________________________

VACAS
Cuantos Vacas? ________________________________

&iquestEn los &uacuteltimos 6 meses les ha dado antibi&oacuteticos a su/sus vaca/vacas?
- ◯ a. Si
- ◯ b. No
- ◯ c. No sabe
- ◯ d. Se reh&uacutesa a responder

DATOS MEDICINA VACAS
Nombre de la Medicina/Antibiotico ________________________________

Caja de la medicina o botella vista (si/no)
- ◯ Si
- ◯ No

Se puede leer el nombre?
- ◯ Si
- ◯ No

&iquestCon qu&eacute frecuencia les da esta medicina a sus animales?
- ◯ Cada mes
- ◯ Cada tres meses
- ◯ Cada 6 meses
- ◯ Cada a&ntildeo
- ◯ Cada vez que es necesario
- ◯ No sabe
- ◯ No responde

&iquestEn los &uacuteltimos 6 meses ha dejado de tomar la leche, comer los huevos o comer la carne del animal indicado, despu&eacutes de darle antibi&oacuteticos?
- ◯ SI
- ◯ No

&iquestDurante cuanto tiempo ha dejado de tomarla? (Numero de d&iacuteas) ________________________________

OVEJAS
Cuantos Ovejas? ________________________________

&iquestEn los &uacuteltimos 6 meses les ha dado antibi&oacuteticos a su/sus oveja/ovejas?
- ◯ a. Si
- ◯ b. No
- ◯ c. No sabe
- ◯ d. Se reh&uacutesa a responder

DATOS MEDICINA OVEJAS
Nombre de la Medicina/Antibiotico ________________________________

Caja de la medicina o botella vista (si/no)
- ◯ Si
- ◯ No

Se puede leer el nombre?
- ◯ Si
- ◯ No

&iquestCon qu&eacute frecuencia les da esta medicina a sus animales?
- ◯ Cada mes
- ◯ Cada tres meses
- ◯ Cada 6 meses
- ◯ Cada a&ntildeo
- ◯ Cada vez que es necesario
- ◯ No sabe
- ◯ No responde

&iquestEn los &uacuteltimos 6 meses ha dejado de tomar la leche, comer los huevos o comer la carne del animal indicado, despu&eacutes de darle antibi&oacuteticos?
- ◯ SI
- ◯ No

&iquestDurante cuanto tiempo ha dejado de tomarla? (Numero de d&iacuteas) ________________________________

CABRAS
Cuantos Cabras? ________________________________

&iquestEn los &uacuteltimos 6 meses les ha dado antibi&oacuteticos a su/sus cabra/cabras?
- ◯ a. Si
- ◯ b. No
- ◯ c. No sabe
- ◯ d. Se reh&uacutesa a responder

DATOS MEDICINA CABRA
Nombre de la Medicina/Antibiotico ________________________________

Caja de la medicina o botella vista (si/no)
- ◯ Si
- ◯ No

Se puede leer el nombre?
- ◯ Si
- ◯ No

&iquestCon qu&eacute frecuencia les da esta medicina a sus animales?

- ◯ Cada mes
- ◯ Cada tres meses
- ◯ Cada 6 meses
- ◯ Cada a&ntildeo
- ◯ Cada vez que es necesario
- ◯ No sabe
- ◯ No responde

&iquestEn los &uacuteltimos 6 meses ha dejado de tomar la leche, comer los huevos o comer la carne del animal indicado, despu&eacutes de darle antibi&oacuteticos?

- ◯ SI
- ◯ No

&iquestDurante cuanto tiempo ha dejado de tomarla? (Numero de d&iacuteas) __________________________________

OTRO ANIMALES
Cuantos otros animales? __________________________________

&iquestEn los &uacuteltimos 6 meses les ha dado antibi&oacuteticos a su/sus otros animales?

- ◯ a. Si
- ◯ b. No
- ◯ c. No sabe
- ◯ d. Se reh&uacutesa a responder

DATOS MEDICINA OTROS ANIMALES
Nombre de la Medicina/Antibiotico __________________________________

Caja de la medicina o botella vista (si/no)

- ◯ Si
- ◯ No

Se puede leer el nombre?

- ◯ Si
- ◯ No

&iquestCon qu&eacute frecuencia les da esta medicina a sus animales?

- ◯ Cada mes
- ◯ Cada tres meses
- ◯ Cada 6 meses
- ◯ Cada a&ntildeo
- ◯ Cada vez que es necesario
- ◯ No sabe
- ◯ No responde

&iquestEn los &uacuteltimos 6 meses ha dejado de tomar la leche, comer los huevos o comer la carne del animal indicado, despu&eacutes de darle antibi&oacuteticos?

- ◯ SI
- ◯ No

&iquestDurante cuanto tiempo ha dejado de tomarla? (Numero de d&iacuteas) __________________________________

12.&iquestLos animales a veces se quedan dentro de los cuartos en donde duermen los miembros de la familia(ya sea de d&iacutea o de noche)? (MARQUE UNO)

- ◯ a. Si
- ◯ b. No
- ◯ c. No sabe
- ◯ d. Se reh&uacutesa a responder

13.Por favor indique &iquestQu&eacute animales se quedan a veces adentro de los cuartos donde duermen miembros de la familia (ya sea de noche o de d&iacutea)?(MARQUE TODOS LOS QUE APLIQUEN indicando cantidad)

- ☐ a. Perros
- ☐ b. Gatos
- ☐ c. Pollos
- ☐ d. Pavos
- ☐ e. Patos
- ☐ f. Gansos
- ☐ g. Otros p&aacutejaros
- ☐ h. Cerdos
- ☐ i. Vacas
- ☐ j. Ovejas
- ☐ k. Cabras
- ☐ l. Otros animales
- ☐ m. No sabe
- ☐ n. Se reh&uacutesa a responder

a. Perros ______________________________

b. Gatos ______________________________

c. Pollos ______________________________

d. Pavos ______________________________

e. Patos ______________________________

f. Gansos ______________________________

g. Otros p&aacutejaros ______________________________

h. Cerdos ______________________________

i. Vacas ______________________________

j. Ovejas ______________________________

k. Cabras ______________________________

l. Otros, especificar ______________________________

Cuestionario Adultos

I. Buenos d&iacuteas/tardes, mi nombre es (NOMBRE PERSONA HABLANDO) y vengo hoy con mi compa&ntildeero de trabajo (NOMBRE COMPA&NtildeERO DEL TRABAJO). Estamos visitando su casa el d&iacutea de hoy porque estamos realizando la segunda fase del estudio sobre la resistencia a los antibi&oacuteticos. &iquestSer&iacutea posible hablar con [NOMBRE DE LA PERSONA QUE PARTICIP&Oacute ANTERIORMENTE] para hacerle unas preguntas y colectar nuevamente la muestra de heces?	○ a.Si- continuar al II<br>○ b. No, [NOMBRE DE LA PERSONA ELIGIDA] no est&aacute. Preguntar a qu&eacute hora pueden regresar para hablar con la persona que particip&oacute anteriormente..<br>○ c.Persona se niega a participar en la segunda fase.
1. Usted esta enfermo el d&iacutea de hoy con diarrea, fiebre aguda o una enfermedad respiratoria?	○ a. S&iacute- Finalizado. Deber&aacute regresar a hablar con este participante en una semana.<br>○ b. No
2. Usted esta tomando alg&uacuten medicamento el d&iacutea de hoy?	○ a. S&iacute<br>○ b. No
3. Nos puede ense&ntildear que medicamento est&aacute tomando(la caja, sobre o blister)?	○ a. S&iacute<br>○ b. No, no lo puede mostrar
3.1 Nos puede decir cual medicamento esta tomando?	○ a. S&iacute. Consultar el listado para ver si se trata de un antibi&oacutetico. Si est&aacute en la lista de antibi&oacuteticos no puede participar hoy; usted deber&aacute regresar a hablar con este participante cuando ya no est&eacute tomando antibi&oacutetico (preguntarle cu&aacutendo). Si no est&aacute en la lista, llamar a Puri o Brooke para aclarar si se trata de un antibi&oacutetico o no.<br>○ b. No, no lo puede decir. Finalizado.
3.2. &iquestEl medicamento que est&aacute tomando hoy es un antibi&oacutetico?	○ Si. Regresar al cabo de una semana.<br>○ No

INFORMACI&OacuteN DEMOGR&AacuteFICA del representante del hogar

1. Codigo/identificacion (xxx-xx)	________________________
Latitud	________________________
Longitud	________________________
2. Localidad	○ Coatepeque<br>○ San Juan Ostuncalco
a. Coatepeque (listado definido de opciones)	○ a. El Jardin<br>○ b. La Union
b. San Juan Ostuncalco (listado definido de opciones)	○ 1.Casco Urbano<br>○ 2.Monrovia
3. Sexo	○ a. Masculino<br>○ b. Femenino

I. Enfermedades Previas-Diarrea, Febriles y Respiratoria: Ahora quisiera preguntarle sobre eventos previos de enfermedad diarreica, febril o respiratoria..

4. &iquestEn los &uacuteltimos 14 d&iacuteas, ha tenido diarrea?	◯ a. Si<br>◯ b. No<br>◯ c. No sabe<br>◯ d. Se reh&uacutesa a responder
5. Tom&oacute alg&uacuten medicamento para tratar este episodio de diarrea/asientos?	◯ a. Si<br>◯ b. No<br>◯ c. No sabe<br>◯ d. Se reh&uacutesa a responder
6. &iquestQue medicamentos tom&oacute para tratar este episodio de diarrea/asientos?	☐ a. Antibi&oacuteticos<br>☐ b. Analg&eacutesicos (medicamentos para el dolor)<br>☐ c. Desparasitantes/antihelm&iacutenticos<br>☐ d. Remedios de hierbas, especificar<br>☐ e. Otros,<br>☐ f. Ninguno
Nombre del otro medicamento	________________________________
Remedios de hierbas:	________________________________
7. &iquestEn los &uacuteltimos 14 d&iacuteas, ha tenido fiebre?	◯ a. Si<br>◯ b. No<br>◯ c. No sabe<br>◯ d. Se reh&uacutesa a responder
8. Tom&oacute alg&uacuten medicamento para tratar este episodio de fiebre?	◯ a. Si<br>◯ b. No<br>◯ c. No sabe<br>◯ d. Se reh&uacutesa a responder
9. &iquestQue medicamentos tom&oacute para tratar este episodio de fiebre?	☐ a. Antibi&oacuteticos<br>☐ b. Analg&eacutesicos (medicamentos para el dolor)<br>☐ c. Desparasitantes/antihelm&iacutenticos<br>☐ d. Remedios de hierbas, especificar<br>☐ e. Otros<br>☐ f. Ninguno
Nombre del otro medicamento	________________________________
Remedios de hierbas:	________________________________
10. En los &uacuteltimos 14 d&iacuteas, &iquestha tenido gripe, tos o alg&uacuten sintoma respiratorio?	◯ a. Si<br>◯ b. No<br>◯ c. No sabe<br>◯ d. Se reh&uacutesa a responder
11. Tom&oacute alg&uacuten medicamento para tratar este episodio de gripe/tos?	◯ a. Si<br>◯ b. No<br>◯ c. No sabe<br>◯ d. Se reh&uacutesa a responder
12. &iquestQue medicamentos tom&oacute para tratar este episodio de gripe/tos?	☐ a. Antibi&oacuteticos<br>☐ b. Analg&eacutesicos (medicamentos para el dolor)<br>☐ c. Desparasitantes/antihelm&iacutenticos<br>☐ d. Remedios de hierbas, especificar<br>☐ e. Otros<br>☐ f. Ninguno
Nombre del otro medicamento	________________________________
Remedios de hierbas:	________________________________

II. Historia de uso antibi&oacutetico

En la siguiente secci&oacuten de la encuesta le preguntaremos sobre el uso de medicamentos, especialmente, el uso de antibi&oacuteticos. Los antibi&oacuteticos se definen como medicamentos usados para matar las bacterias. Las bacterias son microorganismos que causan infecciones. Los antibi&oacuteticos se venden en las farmacias de Guatemala y algunas veces en tiendas y supermercados. El Ministerio de Salud P&uacuteblica tambi&eacuten proporciona antibi&oacuteticos a los que tienen una receta de un m&eacutedico del MSPAS. Los antibi&oacuteticos m&aacutes comunes son amoxicilina y tetraciclina (mostrar ejemplos adquiridos del ministerio de salud y tiendas cercanas) y frecuentemente se recomiendan despu&eacutes de visitar al doctor. Algunas veces, las personas deciden tomar antibi&oacuteticos sin tener la recomendaci&oacuten del doctor.

13.&iquestGuard&oacute la bolsa de medicamentos que le entregamos la &uacuteltima vez que estuvimos aqu&iacute?

- ◯ a. Si
- ◯ b. No
- ◯ c. No sabe
- ◯ d. Se reh&uacutesa a responder

&iquestCu&aacutentos medicamentos hay en la bolsa (Cajas, empaques individuales, bl&iacutester, jarabe, etc.)? ________________________________

14. Documentaci&oacuten de medicamentos y foto: ENCUESTADOR: Por favor anote los datos de cada empaque o medicamento individual que est&aacute en la bolsa.

MEDICAMENTO 1
Especifique Marca? ________________________________

Especifique Principio Activo? ________________________________

Especifique Forma farmac&eacuteutica (tableta / c&aacutepsula / jarabe / inyetable / supositorio / otro)? ________________________________

Especifique Dosis? ________________________________

MEDICAMENTO 2
Especifique Marca? ________________________________

Especifique Principio Activo? ________________________________

Especifique Forma farmac&eacuteutica (tableta / c&aacutepsula / jarabe / inyetable / supositorio / otro)? ________________________________

Especifique Dosis? ________________________________

MEDICAMENTO 3
Especifique Marca? ________________________________

Especifique Principio Activo? ________________________________

Especifique Forma farmac&eacuteutica (tableta / c&aacutepsula / jarabe / inyetable / supositorio / otro)? ________________________________

Especifique Dosis? ________________________________

MEDICAMENTO 4
Especifique Marca? ________________________________

Especifique Principio Activo? ________________________________

Especifique Forma farmac&eacuteutica (tableta / c&aacutepsula / jarabe / inyetable / supositorio / otro)? ______________________

Especifique Dosis? ______________________

MEDICAMENTO 5
Especifique Marca? ______________________

Especifique Principio Activo? ______________________

Especifique Forma farmac&eacuteutica (tableta / c&aacutepsula / jarabe / inyetable / supositorio / otro)? ______________________

Especifique Dosis? ______________________

Ahora le har&eacute unas preguntas sobre los antibi&oacuteticos que est&aacuten en la bolsa (ENCUESTADOR: Corroborar con la lista de antibi&oacuteticos.); si no hay bolsa o en la bolsa no hay antibi&oacuteticos le pedir&eacute que me indique si ha tomado alguno de estos antibi&oacuteticos desde la &uacuteltima vez que estuvimos aqu&iacute (ense&ntildear &aacutelbum).

15. &iquestHa tomado antibi&oacuteticos desde la &uacuteltima vez que estuvimos aqu&iacute? (Incluyendo los de la bolsa)

- ◯ a. Si
- ◯ b. No
- ◯ c. No sabe
- ◯ d. Se reh&uacutesa a responder

&iquestCu&aacutentos antibi&oacuteticos ha tomado desde la &uacuteltima vez que estuvimos aqu&iacute, incluyendo los de la bolsa y otros que no haya guardado en la bolsa? ______________________

16. Para cada antibi&oacutetico complete la siguiente tabla:

DATOS MEDICINA No 1
Nombre de la medicina (1) ______________________

&iquestSe examin&oacute paquete o botella?

- ◯ Si
- ◯ No

&iquestEl nombre del medicamento se puede leer?

- ◯ Si
- ◯ No

La ultima vez que tom&oacute un antibi&oacutetico, para qu&eacute enfermedad lo tom&oacute? (puede seleccionar mas de una)

- ☐ a. Nariz congestionada
- ☐ b. Tos
- ☐ c. Dolor de cabeza
- ☐ d. Fiebre
- ☐ e. V&oacutemitos
- ☐ f. Diarrea
- ☐ g. Heridas de la piel
- ☐ h. Malaria
- ☐ i. VIH
- ☐ j. Zika
- ☐ k. Dengue
- ☐ l. Chikungunya
- ☐ m. Tuberculosis
- ☐ n. Otro. Especificar.
- ☐ o. No Sabe
- ☐ p. Se reh&uacutesa a responder

Otro ______________________

&iquestLe recetaron este medicamento en un puesto/centro/cl&iacutenica privada de salud?

- ◯ Si
- ◯ No
- ◯ No se
- ◯ No Responde

Indique en qu&eacute lugar se le recet&oacute/recomend&oacute.

- ◯ Centro de salud MSPAS
- ◯ Puesto de salud/Cl&iacutenica privada

Si no, &iquestC&oacutemo sabia qu&eacute medicamento comprar?

- ◯ Amigo o familiar
- ◯ Experiencia previa
- ◯ Persona en la farmacia
- ◯ M&eacutedico
- ◯ Otro

Especifique otro... __________________________________

&iquestEn donde consigui&oacute el medicamento?

- ◯ Farmacia
- ◯ Tienda
- ◯ Super
- ◯ MSPAS
- ◯ Amigo o familiar
- ◯ Comadronas
- ◯ Curanderos
- ◯ Otros

&iquestCuantos d&iacuteas tomo el medicamento? __________________________________

&iquestCon que frecuencia toma este medicamento?

- ◯ Cada mes
- ◯ Cada tres meses
- ◯ Cada 6 meses
- ◯ Cada a&ntildeo
- ◯ Cada vez que es necesario
- ◯ No sabe
- ◯ No responde

Ademas de usted, &iquestuso otra persona o animal este medicamento?

- ◯ SI
- ◯ No

&iquestQu&eacute otra persona o animal us&oacute este medicamento? __________________________________

DATOS MEDICINA No 2
Nombre de la medicina (2) __________________________________

&iquestSe examin&oacute paquete o botella?

- ◯ Si
- ◯ No

&iquestEl nombre del medicamento se puede leer?

- ◯ Si
- ◯ No

La ultima vez que tom&oacute un antibi&oacutetico, para qu&eacute enfermedad lo tom&oacute? (puede seleccionar mas de una)

- ☐ a. Nariz congestionada
- ☐ b. Tos
- ☐ c. Dolor de cabeza
- ☐ d. Fiebre
- ☐ e. V&oacutemitos
- ☐ f. Diarrea
- ☐ g. Heridas de la piel
- ☐ h. Malaria
- ☐ i. VIH
- ☐ j. Zika
- ☐ k. Dengue
- ☐ l. Chikungunya
- ☐ m. Tuberculosis
- ☐ n. Otro. Especificar.
- ☐ o. No Sabe
- ☐ p. Se reh&uacutesa a responder

Otro ______________________________

&iquestLe recetaron este medicamento en un puesto/centro/cl&iacutenica privada de salud?

- ◯ Si
- ◯ No
- ◯ No se
- ◯ No Responde

Indique en qu&eacute lugar se le recet&oacute/recomend&oacute.

- ◯ Centro de salud MSPAS
- ◯ Puesto de salud/Cl&iacutenica privada

Si no, &iquestC&oacutemo sabia qu&eacute medicamento comprar?

- ◯ Amigo o familiar
- ◯ Experiencia previa
- ◯ Persona en la farmacia
- ◯ M&eacutedico
- ◯ Otro

Especifique otro... ______________________________

&iquestEn donde consigui&oacute el medicamento?

- ◯ Farmacia
- ◯ Tienda
- ◯ Super
- ◯ MSPAS
- ◯ Amigo o familiar
- ◯ Comadronas
- ◯ Curanderos
- ◯ Otros

&iquestCuantos d&iacuteas tomo el medicamento? ______________________________

&iquestCon que frecuencia toma este medicamento?

- ◯ Cada mes
- ◯ Cada tres meses
- ◯ Cada 6 meses
- ◯ Cada a&ntildeo
- ◯ Cada vez que es necesario
- ◯ No sabe
- ◯ No responde

Ademas de usted, &iquestuso otra persona o animal este medicamento?

- ◯ SI
- ◯ No

&iquestQu&eacute otra persona o animal us&oacute este medicamento? ______________________________

DATOS MEDICINA No 3
Nombre de la medicina (3) ________________________________

&iquestSe examin&oacute paquete o botella?
- ◯ Si
- ◯ No

&iquestEl nombre del medicamento se puede leer?
- ◯ Si
- ◯ No

La ultima vez que tom&oacute un antibi&oacutetico, para qu&eacute enfermedad lo tom&oacute? (puede seleccionar mas de una)
- ☐ a. Nariz congestionada
- ☐ b. Tos
- ☐ c. Dolor de cabeza
- ☐ d. Fiebre
- ☐ e. V&oacutemitos
- ☐ f. Diarrea
- ☐ g. Heridas de la piel
- ☐ h. Malaria
- ☐ i. VIH
- ☐ j. Zika
- ☐ k. Dengue
- ☐ l. Chikungunya
- ☐ m. Tuberculosis
- ☐ n. Otro. Especificar.
- ☐ o. No Sabe
- ☐ p. Se reh&uacutesa a responder

Otro ________________________________

&iquestLe recetaron este medicamento en un puesto/centro/cl&iacutenica privada de salud?
- ◯ Si
- ◯ No
- ◯ No se
- ◯ No Responde

Indique en qu&eacute lugar se le recet&oacute/recomend&oacute.
- ◯ Centro de salud MSPAS
- ◯ Puesto de salud/Cl&iacutenica privada

Si no, &iquestC&oacutemo sabia qu&eacute medicamento comprar?
- ◯ Amigo o familiar
- ◯ Experiencia previa
- ◯ Persona en la farmacia
- ◯ M&eacutedico
- ◯ Otro

Especifique otro... ________________________________

&iquestEn donde consigui&oacute el medicamento?
- ◯ Farmacia
- ◯ Tienda
- ◯ Super
- ◯ MSPAS
- ◯ Amigo o familiar
- ◯ Comadronas
- ◯ Curanderos
- ◯ Otros

&iquestCuantos d&iacuteas tomo el medicamento? ________________________________

&iquestCon que frecuencia toma este medicamento?

- ◯ Cada mes
- ◯ Cada tres meses
- ◯ Cada 6 meses
- ◯ Cada a&ntildeo
- ◯ Cada vez que es necesario
- ◯ No sabe
- ◯ No responde

Ademas de usted, &iquestuso otra persona o animal este medicamento?

- ◯ SI
- ◯ No

&iquestQu&eacute otra persona o animal us&oacute este medicamento? __________________________________

III. Exposici&oacuten a hospitales y servicios de salud: Ahora quisiera hacerle unas preguntas sobre si ha visitado el hospital o servicio de salud.

17. &iquestUsted trabaja en el hospital?

- ◯ a.Si
- ◯ b.No

18. &iquestEn los &uacuteltimos 3 meses, ha visitado el hospital por alguna raz&oacuten?

- ◯ a. Si
- ◯ b. No
- ◯ c. No sabe
- ◯ d. Reh&uacutesa

19. &iquestPor que visito el hospital?

- ◯ a. Para hacer un tramite
- ◯ b. para visitar a un paciente internado/Acompa&ntildear a un familiar
- ◯ c. Para un chequeo m&eacutedico rutinario
- ◯ d. Para recibir un tratamiento
- ◯ e. Porque estaba enfermo y me internaron
- ◯ f. Otro
- ◯ g. No sabe
- ◯ h. Se reh&uacutesa a responder

Otro __________________________________

20. En los &uacuteltimos 3 meses, &iquestha visitado el centro de salud por alguna raz&oacuten?

- ◯ a. Si
- ◯ b. No
- ◯ c. No sabe
- ◯ d. Se reh&uacutesa a responder

21. &iquestPor que visito el centro de salud?

- ◯ a. Para hacer un tramite
- ◯ b. para visitar a un paciente internado/Acompa&ntildear a un familiar
- ◯ c. Para un chequeo m&eacutedico rutinario
- ◯ d. para recibir un tratamiento
- ◯ e. porque estaba enferma y me internaron
- ◯ f. Otro, especificar
- ◯ g. No sabe
- ◯ h. Se reh&uacutesa a responder

Otro __________________________________

Cuestionario Niño menor de 5 Años

I.Gracias por su participaci&oacuten en el proyecto. Como le comentamos al principio, tambi&eacuten estamos interesados en la participaci&oacuten en la segunda fase de ______________ (el mismo ni&ntildeo que particip&oacute la primera vez). &iquestPodemos hablar con _______________ (nombre de la persona que dio el consentimiento por el ni&ntildeo)?

- ◯ a.Si- continuar a la pregunta 1.
- ◯ b. No, no est&aacute la persona que dio el consentimiento por el/la ni&ntildeo(a). Preguntar a qu&eacute hora pueden regresar para hablar con la persona que particip&oacute anteriormente.
- ◯ c. Persona se niega a participar en la segunda fase- terminar.

1. &iquestEl ni&ntildeo est&aacute enfermo el d&iacutea de hoy con diarrea, fiebre aguda o una enfermedad respiratoria severa?

- ◯ a. S&iacute- Solicitar al participante que le llame cuando el/la ni&ntildeo(a) se mejore. De no recibir una llamada, deber&aacute regresar a hablar con este participante en una semana.
- ◯ b. No

2. &iquestEl ni&ntildeo est&aacute tomando alg&uacuten medicamento el d&iacutea de hoy?

- ◯ a. S&iacute
- ◯ b. No

3. Nos puede ense&ntildear qu&eacute medicamento est&aacute tomando el/la ni&ntildeo(a) (la caja, sobre o bl&iacutester)?

- ◯ S&iacute, consultar el listado para ver si se trata de un antibi&oacutetico. Si est&aacute en la lista de antibi&oacuteticos no puede participar hoy; usted deber&aacute regresar a hablar con el padre o madre de este participante cuando ya no est&eacute tomando antibi&oacutetico (preguntarle cu&aacutendo). Si no est&aacute en la lista, llamar a Puri y Brooke para aclarar si se trata de un antibi&oacutetico o no.
- ◯ No, no lo puede mostrar

3.1 &iquestNos puede decir cual medicamento esta tomando?

- ◯ a. S&iacute, consultar el listado para ver si se trata de un antibi&oacutetico. Si est&aacute en la lista de antibi&oacuteticos no puede participar hoy; usted deber&aacute regresar a hablar con el padre o madre de este participante cuando ya no est&eacute tomando antibi&oacutetico (preguntarle cu&aacutendo). Si no est&aacute en la lista, llamar a Puri y Brooke para aclarar si se trata de un antibi&oacutetico o no.
- ◯ b. No, no lo puede mostrar.

3.2. &iquestEl medicamento que est&aacute tomando hoy es un antibi&oacutetico?

- ◯ Si
- ◯ No

INFORMACI&OacuteN DEMOGR&AacuteFICA del ni&ntildeo participante

1. Codigo/identificacion (xxx-xx) __________________________________

Latitud __________________________________

Longitud __________________________________

2. Localidad

- ◯ Coatepeque
- ◯ San Juan Ostuncalco

a. Coatepeque (listado definido de opciones)

- ◯ a. El Jardin
- ◯ b. La Union

b. San Juan Ostuncalco (listado definido de opciones)

- ◯ 1.Casco Urbano
- ◯ 2.Monrovia

3. Sexo

◯ a. Masculino
◯ b. Femenino

I.Pr&aacutecticas de la vivienda incluyendo posesi&oacuten de animales, m&eacutetodos de cocci&oacuten y dieta

Secci&oacuten 1 ENCUESTADOR LEA: Muchas gracias. Ahora quiero hacerle unas preguntas sobre varias condiciones en su hogar, que aplican al ni&ntildeo. Estas preguntas las hacemos en cada casa que visitamos, y a cada participante que encuestamos. Comencemos con preguntas sobre la comida del ni&ntildeo.

4. &iquestEl ni&ntildeo come carne?

◯ a. Si
◯ b. No
◯ c. No sabe
◯ d. Se reh&uacutesa a responder

5. &iquestQu&eacute tipo de carne? (puede responder a varios)

☐ a. Pollo
☐ b. Carne de cerdo
☐ c. Carne de res
☐ d. Pescado
☐ e. Conejo
☐ f. Chompipe
☐ g. Pato
☐ h. Otro, indique
☐ i. No sabe
☐ j. Se reh&Uacutesa a responder

&iquestQu&eacute otro tipo de carne? ______________________________

6. Indique la procedencia de cada tipo de carne que el ni&ntildeo come

☐ a. Criado en la casa
☐ b. Comprado en el mercado
☐ c. Comprado en el supermercado
☐ d. Comprado en la despensa
☐ e. Comprado en la tienda
☐ f. Otro, especificar
☐ g. No sabe
☐ h. Se reh&uacutesa a responder

Otro ______________________________

7. &iquestEl ni&ntildeo toma leche?

◯ a. Si
◯ b. No
◯ c. No sabe
◯ d. Se reh&uacutesa a responder

8. &iquestQu&eacute tipo de leche toma? (MARQUE TODOS LOS QUE APLIQUEN)

☐ a. Leche Materna
☐ b. Leche de vaca (en fresco)
☐ c. Leche en polvo
☐ d. Leche en caja
☐ e. Leche de cabra (en fresco)
☐ f. Leche en bolsa
☐ g. Otro

Otro ______________________________

9. &iquestTrata (o prepara) de alguna forma la leche de vaca o cabra (en fresco) antes de tomarla?

- a. Si
- b. No
- c. No sabe
- d. Se reh&uacutesa a responder

10. &iquestHierve la leche antes de tomarla?

- a. Si
- b. No
- c. No sabe
- d. Se reh&uacutesa a responder

II.Enfermedades Previas-Diarrea y exposici&oacuten a hospitales: Ahora quisiera preguntarle sobre eventos previos de enfermedad diarreica del ni&ntildeo.

11. &iquestEn los &uacuteltimos 14 d&iacuteas, ha tenido diarrea el ni&ntildeo?

- a. Si
- b. No
- c. No sabe
- d. Se reh&uacutesa a responder

12. &iquestEl ni&ntildeo tom&oacute alg&uacuten medicamento para tratar este episodio de diarrea/asientos?

- a. Si
- b. No
- c. No sabe
- d. Se reh&uacutesa a responder

13. &iquestQue medicamentos tom&oacute el ni&ntildeo para tratar este episodio de diarrea/asientos?

- a. Antibi&oacuteticos
- b. Analg&eacutesicos (medicamentos para el dolor)
- c. Desparasitantes/antihelm&iacutenticos
- d. Remedios de hierbas, especificar
- e. Otros,
- f. Ninguno

Nombre del otro medicamento ________________________________

Remedios de hierbas: ________________________________

14. &iquestEn los &uacuteltimos 14 d&iacuteas, ha tenido fiebre el ni&ntildeo?

- a. Si
- b. No
- c. No sabe
- d. Se reh&uacutesa a responder

15. &iquestEl ni&ntildeo tom&oacute alg&uacuten medicamento para tratar este episodio de fiebre?

- a. Si
- b. No
- c. No sabe
- d. Se reh&uacutesa a responder

16. &iquestQue medicamentos tom&oacute el ni&ntildeo para tratar este episodio de fiebre?

- a. Antibi&oacuteticos
- b. Analg&eacutesicos (medicamentos para el dolor)
- c. Desparasitantes/antihelm&iacutenticos
- d. Remedios de hierbas, especificar
- e. Otros
- f. Ninguno

Nombre del otro medicamento ________________________________

Remedios de hierbas: ________________________________

17. En los &uacuteltimos 14 d&iacuteas, &iquestha tenido el ni&ntildeo gripe, tos o alg&uacuten s&iacutentoma respiratorio?

- a. Si
- b. No
- c. No sabe
- d. Se reh&uacutesa a responder

18. &iquestEl ni&ntildeo tom&oacute alg&uacuten medicamento para tratar este episodio de gripe/tos?

◯ a. Si
◯ b. No
◯ c. No sabe
◯ d. Se reh&uacutesa a responder

19. &iquestQue medicamentos tom&oacute el ni&ntildeo para tratar este episodio de gripe/tos?

☐ a. Antibi&oacuteticos
☐ b. Analg&eacutesicos (medicamentos para el dolor)
☐ c. Desparasitantes/antihelm&iacutenticos
☐ d. Remedios de hierbas, especificar
☐ e. Otros
☐ f. Ninguno

Nombre del otro medicamento ______________________________

Remedios de hierbas: ______________________________

III.Historia de uso antibi&oacutetico

En la siguiente secci&oacuten de la encuesta le preguntaremos sobre el uso de medicamentos, especialmente, el uso de antibi&oacuteticos por el ni&ntildeo. Los antibi&oacuteticos se definen como medicamentos usados para matar las bacterias. Las bacterias son microorganismos que causan infecciones. Los antibi&oacuteticos se venden en las farmacias de Guatemala y algunas veces en tiendas y supermercados. El Ministerio de Salud P&uacuteblica tambi&eacuten proporciona antibi&oacuteticos a los que tienen una receta de un m&eacutedico del MSPAS. Los antibi&oacuteticos m&aacutes comunes son amoxicilina y tetraciclina (mostrar ejemplos adquiridos del ministerio de salud y tiendas cercanas) y frecuentemente se recomiendan despu&eacutes de visitar al doctor. Algunas veces, las personas deciden tomar o darles antibi&oacuteticos a sus ni&ntildeos sin tener la recomendaci&oacuten del doctor.

20. &iquestGuard&oacute la bolsa de medicamentos que le entregamos la &uacuteltima vez que estuvimos aqu&iacute?

◯ a. Si
◯ b. No
◯ c. No sabe
◯ d. Se reh&uacutesa a responder

&iquestCu&aacutentos medicamentos hay en la bolsa (Cajas, empaques individuales, bl&iacutester, jarabe, etc.)? ______________________________

21. Documentaci&oacuten de medicamentos y foto: ENCUESTADOR: Por favor anote los datos de cada empaque o medicamento individual que est&aacute en la bolsa.

MEDICAMENTO 1
Especifique Marca? ______________________________

Especifique Principio Activo? ______________________________

Especifique Forma farmac&eacuteutica (tableta / c&aacutepsula / jarabe / inyetable / supositorio / otro)? ______________________________

Especifique Dosis? ______________________________

MEDICAMENTO 2
Especifique Marca? ______________________________

Especifique Principio Activo? ________________

Especifique Forma farmac&eacuteutica (tableta / c&aacutepsula / jarabe / inyetable / supositorio / otro)? ________________

Especifique Dosis? ________________

MEDICAMENTO 3
Especifique Marca? ________________

Especifique Principio Activo? ________________

Especifique Forma farmac&eacuteutica (tableta / c&aacutepsula / jarabe / inyetable / supositorio / otro)? ________________

Especifique Dosis? ________________

MEDICAMENTO 4
Especifique Marca? ________________

Especifique Principio Activo? ________________

Especifique Forma farmac&eacuteutica (tableta / c&aacutepsula / jarabe / inyetable / supositorio / otro)? ________________

Especifique Dosis? ________________

MEDICAMENTO 5
Especifique Marca? ________________

Especifique Principio Activo? ________________

Especifique Forma farmac&eacuteutica (tableta / c&aacutepsula / jarabe / inyetable / supositorio / otro)? ________________

Especifique Dosis? ________________

Ahora le har&eacute unas preguntas sobre los antibi&oacuteticos que est&aacuten en la bolsa (ENCUESTADOR: Corroborar con la lista de antibi&oacuteticos.); si no hay bolsa o en la bolsa no hay antibi&oacuteticos le pedir&eacute que me indique si ha tomado alguno de estos antibi&oacuteticos desde la &uacuteltima visita (ense&ntildear &aacutelbum).

22. &iquestEl ni&ntildeo ha tomado antibi&oacuteticos desde la &uacuteltima visita? (Incluyendo los de la bolsa)

- ◯ a. Si
- ◯ b. No
- ◯ c. No sabe
- ◯ d. Se reh&uacutesa a responder

&iquestCu&aacutentos antibi&oacuteticos ha tomado desde la &uacuteltima visita, incluyendo los de la bolsa y otros que no haya guardado en la bolsa? ________________

23. Para cada antibi&oacutetico complete la siguiente tabla:

DATOS MEDICINA No 1
Nombre de la medicina (1) ________________

&iquestSe examin&oacute paquete o botella?

- ◯ Si
- ◯ No

&iquestEl nombre del medicamento se puede leer?
- ◯ Si
- ◯ No

La ultima vez que tomo un antibi&oacutetico, para que enfermedad lo tomo? (puede seleccionar mas de una)
- ☐ a. Nariz congestionada
- ☐ b. Tos
- ☐ c. Dolor de cabeza
- ☐ d. Fiebre
- ☐ e. V&oacutemitos
- ☐ f. Diarrea
- ☐ g. Heridas de la piel
- ☐ h. Malaria
- ☐ i. VIH
- ☐ j. Zika
- ☐ k. Dengue
- ☐ l. Chikungunya
- ☐ m. Tuberculosis
- ☐ n. Otro. Especificar.
- ☐ o. No Sabe
- ☐ p. Se reh&uacutesa a responder

Otro ________________________________

&iquestLe recetaron este medicamento en un puesto/centro/cl&iacutenica privada de salud?
- ◯ Si
- ◯ No
- ◯ No se
- ◯ No Responde

&iquestEn que lugar se le receto?
- ◯ Centro de salud MSPAS
- ◯ Puesto de salud/Cl&iacutenica privada

Si no, &iquestComo sabia que medicamento comprar?
- ◯ Amigo o familiar
- ◯ Experiencia previa
- ◯ Persona en la farmacia
- ◯ M&eacutedico
- ◯ Otro

Especifique otro... ________________________________

&iquestEn donde consigui&oacute el medicamento?
- ◯ Farmacia
- ◯ Tienda
- ◯ Super
- ◯ MSPAS
- ◯ Amigo o familiar
- ◯ Comadronas
- ◯ Curanderos
- ◯ Otros

&iquestCuantos d&iacuteas tomo el medicamento? ________________________________

&iquestCon que frecuencia toma este medicamento?
- ◯ Cada mes
- ◯ Cada tres meses
- ◯ Cada 6 meses
- ◯ Cada a&ntildeo
- ◯ Cada vez que es necesario
- ◯ No sabe
- ◯ No responde

Ademas de usted, &iquestuso otra persona o animal este medicamento?
- ◯ SI
- ◯ No

&iquestQu&eacute otra persona us&oacute este medicamento? ________________________________

DATOS MEDICINA No 2
Nombre de la medicina (2) ________________________________

&iquestSe examin&oacute paquete o botella?
- ◯ Si
- ◯ No

&iquestEl nombre del medicamento se puede leer?
- ◯ Si
- ◯ No

La ultima vez que tomo un antibi&oacutetico, para que enfermedad lo tomo? (puede seleccionar mas de una)
- ☐ a. Nariz congestionada
- ☐ b. Tos
- ☐ c. Dolor de cabeza
- ☐ d. Fiebre
- ☐ e. V&oacutemitos
- ☐ f. Diarrea
- ☐ g. Heridas de la piel
- ☐ h. Malaria
- ☐ i. VIH
- ☐ j. Zika
- ☐ k. Dengue
- ☐ l. Chikungunya
- ☐ m. Tuberculosis
- ☐ n. Otro. Especificar.
- ☐ o. No Sabe
- ☐ p. Se reh&uacutesa a responder

Otro ________________________________

&iquestLe recetaron este medicamento en un puesto/centro/cl&iacutenica privada de salud?
- ◯ Si
- ◯ No
- ◯ No se
- ◯ No Responde

Indique en qu&eacute lugar se le recet&oacute/recomend&oacute
- ◯ Centro de salud MSPAS
- ◯ Puesto de salud/Cl&iacutenica privada

Si no, &iquestComo sabia que medicamento comprar?
- ◯ Amigo o familiar
- ◯ Experiencia previa
- ◯ Persona en la farmacia
- ◯ M&eacutedico
- ◯ Otro

Especifique otro... ________________________________

&iquestEn donde consigui&oacute el medicamento?
- ◯ Farmacia
- ◯ Tienda
- ◯ Super
- ◯ MSPAS
- ◯ Amigo o familiar
- ◯ Comadronas
- ◯ Curanderos
- ◯ Otros

&iquestCuantos d&iacuteas tomo el medicamento? ________________________________

&iquestCon que frecuencia toma este medicamento?

- ◯ Cada mes
- ◯ Cada tres meses
- ◯ Cada 6 meses
- ◯ Cada a&ntildeo
- ◯ Cada vez que es necesario
- ◯ No sabe
- ◯ No responde

Ademas de usted, &iquestuso otra persona o animal este medicamento?

- ◯ SI
- ◯ No

&iquestQu&eacute otra persona us&oacute este medicamento? __________________________________

DATOS MEDICINA No 3
Nombre de la medicina (3) __________________________________

&iquestSe examin&oacute paquete o botella?

- ◯ Si
- ◯ No

&iquestEl nombre del medicamento se puede leer?

- ◯ Si
- ◯ No

La ultima vez que tomo un antibi&oacutetico, para que enfermedad lo tomo? (puede seleccionar mas de una)

- ☐ a. Nariz congestionada
- ☐ b. Tos
- ☐ c. Dolor de cabeza
- ☐ d. Fiebre
- ☐ e. V&oacutemitos
- ☐ f. Diarrea
- ☐ g. Heridas de la piel
- ☐ h. Malaria
- ☐ i. VIH
- ☐ j. Zika
- ☐ k. Dengue
- ☐ l. Chikungunya
- ☐ m. Tuberculosis
- ☐ n. Otro. Especificar.
- ☐ o. No Sabe
- ☐ p. Se reh&uacutesa a responder

Otro __________________________________

&iquestLe recetaron este medicamento en un puesto/centro/cl&iacutenica privada de salud?

- ◯ Si
- ◯ No
- ◯ No se
- ◯ No Responde

Indique en qu&eacute lugar se le recet&oacute/recoend&oacute.

- ◯ Centro de salud MSPAS
- ◯ Puesto de salud/Cl&iacutenica privada

Si no, &iquestComo sabia que medicamento comprar?

- ◯ Amigo o familiar
- ◯ Experiencia previa
- ◯ Persona en la farmacia
- ◯ M&eacutedico
- ◯ Otro

Especifique otro... __________________________________

&iquestEn donde consigui&oacute el medicamento?

- ◯ Farmacia
- ◯ Tienda
- ◯ Super
- ◯ MSPAS
- ◯ Amigo o familiar
- ◯ Comadronas
- ◯ Curanderos
- ◯ Otros

&iquestCuantos d&iacuteas tomo el medicamento? ________________________________

&iquestCon que frecuencia toma este medicamento?

- ◯ Cada mes
- ◯ Cada tres meses
- ◯ Cada 6 meses
- ◯ Cada a&ntildeo
- ◯ Cada vez que es necesario
- ◯ No sabe
- ◯ No responde

Ademas de usted, &iquestuso otra persona o animal este medicamento?

- ◯ SI
- ◯ No

&iquestQu&eacute otra persona us&oacute este medicamento? ________________________________

24. &iquestEn los &uacuteltimos 3 meses, &iquestel ni&ntildeo ha visitado el hospital por alguna raz&oacuten?

- ◯ a. Si
- ◯ b. No
- ◯ c. No sabe
- ◯ d. Reh&uacutesa

25. &iquestPor que visito el ni&ntildeo el hospital?

- ◯ a. Para hacer un tramite acompa&ntildeando a un adulto
- ◯ b. para visitar a un paciente internado/Acompa&ntildear a un familiar
- ◯ c. Para un chequeo m&eacutedico rutinario
- ◯ d. Para recibir un tratamiento
- ◯ e. Porque estaba enfermo y me internaron
- ◯ f. Otro
- ◯ g. No sabe
- ◯ h. Se reh&uacutesa a responder

Otro ________________________________

26. En los &uacuteltimos 3 meses, &iquestel ni&ntildeo ha visitado el centro de salud por alguna raz&oacuten?

- ◯ a. Si
- ◯ b. No
- ◯ c. No sabe
- ◯ d. Se reh&uacutesa a responder

27. &iquestPor que visito el ni&ntildeo el centro de salud?

- ◯ a. Para hacer un tramite
- ◯ b. para visitar a un paciente internado/Acompa&ntildear a un familiar
- ◯ c. Para un chequeo m&eacutedico rutinario
- ◯ d. para recibir un tratamiento
- ◯ e. porque estaba enferma y me internaron
- ◯ f. Otro, especificar
- ◯ g. No sabe
- ◯ h. Se reh&uacutesa a responder

Otro ________________________________

Consentimiento Informado

CONSENTIMIENTO INFORMADO VERBAL PARA LA REVISI&OacuteN DE LOS RESULTADOS DE LABORATORIO CON LOS PARTICIPANTES

Hola, mi nombre es _____ y estoy hoy aqu&iacute para darle los resultados del examen de par&aacutesitos que le hicimos a la muestra de heces que recolectamos para el proyecto AMR-UVG. Me gustar&iacutea pedirle permiso para revisar los resultados de este examen con usted, para asegurarnos que entiende lo que dicen los resultados y responder a las preguntas que usted pueda tener sobre la b&uacutesqueda de atenci&oacuten en los servicios de salud, en el caso de que lo necesitara. No voy a copiar los resultados de su examen, los resultados son estrictamente confidenciales. No voy a compartir su informaci&oacuten personal de salud con nadie en la comunidad ni de mi equipo a no ser que usted me lo pida.

Revisar los resultados va a tomar aproximadamente 10 minutos. &iquestMe permite revisarlos con usted?

- ◯ S&iacute. Revisar con el participante.
- ◯ No. Dar el sobre sellado sin revisar los resultados. Explicar que alguien del centro o puesto de salud puede ayudar a interpretar el significado de los resultados.

Muestras de laboratorio Adultos

C&oacutedigo ________________________________

Lugar de origen
- ◯ La Union
- ◯ El jardin
- ◯ San Juan Ostuncalco
- ◯ Monrovia

Muestra de Heces
- ◯ Si
- ◯ No

Fecha y hora de recolecci&oacuten (Trabajador de Campo) ________________________________

Fecha y hora de recepci&oacuten en sitio de Hospital ________________________________

Procesamiento en laboratorio

Heces entera
- ◯ Si
- ◯ No

Fecha de elaboraci&oacuten ________________________________

Recepci&oacuten Laboratorio UVG ________________________________

Heces liquida almacenada en crio tubo
- ◯ Si
- ◯ No

Fecha de elaboraci&oacuten ________________________________

Recepci&oacuten Laboratorio UVG ________________________________

Placa MacConkey para aislamiento de E. coli
- ◯ Si
- ◯ No

Fecha de elaboraci&oacuten ________________________________

Recepci&oacuten Laboratorio UVG ________________________________

Placa de 96 pocillos con caldo LB
- ◯ Si
- ◯ No

Fecha de elaboraci&oacuten ________________________________

Recepci&oacuten Laboratorio UVG ________________________________

Placas MacConkey con antibi&oacuteticos
- ◯ Si
- ◯ No

Fecha de elaboraci&oacuten ________________________________

Fecha de elaboraci&oacuten ________________________________

Pruebas de parásitos

¿Se realizo la prueba de parásitos? ◯ Si
◯ No

Fecha en que se realizo la prueba de parásitos ________________________________

¿Resultado de prueba de parásitos? ◯ Helmintos
◯ Protozoos
◯ Ambos
◯ Negativo

Observaciones ________________________________

Muestras de laboratorio Menores de 5 Años

C&oacutedigo ________________________________

Lugar de origen
- ◯ La Union
- ◯ El jardin
- ◯ San Juan Ostuncalco
- ◯ Monrovia

Muestra de Heces
- ◯ Si
- ◯ No

Fecha y hora de recolecci&oacuten (Trabajador de Campo) ________________________________

Fecha y hora de recepci&oacuten en sitio de Hospital ________________________________

Procesamiento en laboratorio

Heces entera
- ◯ Si
- ◯ No

Fecha de elaboraci&oacuten ________________________________

Recepci&oacuten Laboratorio UVG ________________________________

Heces liquida almacenada en crio tubo
- ◯ Si
- ◯ No

Fecha de elaboraci&oacuten ________________________________

Recepci&oacuten Laboratorio UVG ________________________________

Placa MacConkey para aislamiento de E. coli
- ◯ Si
- ◯ No

Fecha de elaboraci&oacuten ________________________________

Recepci&oacuten Laboratorio UVG ________________________________

Placa de 96 pocillos con caldo LB
- ◯ Si
- ◯ No

Fecha de elaboraci&oacuten ________________________________

Recepci&oacuten Laboratorio UVG ________________________________

Placas MacConkey con antibi&oacuteticos
- ◯ Si
- ◯ No

Fecha de elaboraci&oacuten ________________________________

Fecha de elaboraci&oacuten ________________________________

Pruebas de parásitos

¿Se realizó la prueba de parásitos?
◯ Si
◯ No

Fecha en que se realizo la prueba de parásitos __________________________________

¿Resultado de prueba de parásitos?
◯ Helmintos
◯ Protozoos
◯ Ambos
◯ Negativo

Observaciones __________________________________
