# Supplementary material for: Antibiotic use and hygiene interact to influence the distribution of antimicrobial-resistant bacteria in low-income communities in Guatemala
Source: Sci Rep. 2020 Aug 13;10:13767. doi: 10.1038/s41598-020-70741-4 (PMC7426860; doi:10.1038/s41598-020-70741-4)
Supplement: Supplementary file 3 [file 41598_2020_70741_MOESM3_ESM.docx]

**Antibiotic use and hygiene interact to influence the distribution of antimicrobial-resistant bacteria in low-income communities in Guatemala**

**Supplement**

1. **Materials and Methods**

**1.1 Participant sampling**

Participants were included if they had lived in the residence for at least one year (or for children less than one, since the time of birth) and were planning on living in the household for the duration of the study. Participants were not included if they were taking antibiotics on the day of visit, or if they were actively experiencing diarrheal, acute febrile or respiratory illness at the time of the survey. Each household was provided a 2.3 kg bag of rice to compensate their time (all consenting households received the rice even if they elected to not finish the study procedures). The participant stool sample was also analyzed for presence of parasites (service provided by MSPAS) and a confidential report with follow-up instructions was provided to each participant or guardian (follow up medical service provided by MSPAS).

**1.2 Household, adult and child questionnaires and stool sample collection**

Questionnaires were administered to the household representative and to the adult participant and child participant-guardian (where applicable) in phase 1. The same participants enrolled in phase 1 were approached for participation in phase 2 (See Supplemental “Questionnaires”).Questionnaires were designed to identify environmental, socio-economic, behavioral, and demographic variables including antibiotic use (people and animals) and recent episodes of acute illness (2 weeks before study enrollment, or during the 3 months that passed between study phases). These survey instruments were based on similar studies ^1,2^ and included components from water sanitation and health (“WASH”) studies ^3–7^. Participants were instructed to pass stool into any clean and dry container, directly into the study-kit stool collection container, or onto the Kraft paper provided in the study kit (see Supplemental “Instructions for Stool Collection”).

**1.3 Fecal Sampling**

Approximately 1 gram of fecal material was re-suspended in 9 mL of sterile saline solution and thoroughly mixed to obtain a homogeneous suspension. Next, a 10-fold dilution series was prepared (transferring 100 μl volume) for a total of 5 dilutions. Subsequently, 7-10 sterile glass beads and 100 μl of fluid from the last three dilutions were plated to individual MacConkey agar (Merck Millipore, Darmstadt, Germany) plates. After inoculation, glass beads were collected and agar plates were incubated at 37˚C for 16-20 h. Up to 48 colonies per sample with morphology consistent with *E. coli* ^8^ were transferred from MacConkey agar plates into individual wells of 96-well plates containing 200 μl of Luria-Bertani broth (Difco^TM^ LB Broth Lennox, Sparks, MD USA). Only clearly-separated colonies were selected. Where insufficient colonies were available, new plates were prepared from samples that were stored at 4˚C. No further biochemical or nucleic acid markers were used to confirm identity of *E. coli*, although prior work with this same methodology confirmed that 90.7% of presumptive *E. coli* (n = 183) from human stool samples were correctly identified ^9^. After overnight incubation at 37˚C, sterile phosphate buffered glycerol was added into each well (15% vol/vol, final volume of glycerol) and 96-well plates were stored at ‑80˚C until shipped to the Center for Health Studies (Universidad del Valle de Guatemala, Guatemala City) for breakpoint assays.

**1.4 Breakpoint assays**

Susceptibility to different antibiotics was assessed as previously described ^10^. Briefly, 150-mm MacConkey agar plates were prepared with no antibiotic or with one antibiotic as follows: ampicillin (Amp), 32 μg/ml; amoxicillin (Amx), 32 μg/ml; ceftazidime (Caz), 8 μg/ml; chloramphenicol (Chl), 32 μg/ml; ciprofloxacin (Cip), 4 μg/ml; kanamycin (Kan), 64 μg/ml; streptomycin (Str), 16 μg/ml; sulfamethoxazole (Sul), 512 μg/ml; tetracycline (Tet), 16 μg/ml; and trimethoprim (Tri), 8 μg/ml. Antibiotic concentrations were guided by the CLSI standards for *Enterobacteriaceae*, except for streptomycin, which was previously determined by estimating the MIC for the susceptible K-12 strain of *E. coli* used in our study ^10^. All antibiotics were purchased from Sigma-Aldrich (St Louis, MO).

Breakpoint assays were conducted by transferring *E. coli* isolates from thawed culture in LB broth onto the antibiotic-containing media using a 96-pin replicator (Boekel, Scientific, Fisher Scientific). *E. coli* strains K-12 (negative control, susceptible to all antibiotics tested), and strain H4H (positive control, resistant to all of the antibiotics tested save ciprofloxacin ^11^) were used when testing each 96-well plate. After 24-hour incubation at 37˚C, presence of a colony indicated that the isolate was resistant, while no growth was interpreted as susceptible ^10,12^. An agar plate containing no antibiotics was used to confirm isolate viability. The prevalence of antibiotic resistance was estimated by dividing the number of isolates resistant to a given antibiotic by the total number of isolates plated from the same specimen (up to 48).

**1.5 Validation of breakpoint assay results**

We used the Kirby Bauer disc diffusion assay (CLSI, 2016) as a comparator for the breakpoint assay. Antibiotic discs were purchased through Hardy Diagnostics (Sagnta Maria, CA) and included ampicillin (10 ug), ciprofloxacin (5 ug), streptomycin (10 ug), chloramphenicol (30 ug), kanamycin (30 ug), ceftazidime (30 ug), trimethoprim (5 ug), and tetracycline (30 ug). For this comparison amoxicillin/clavulante (20ug/10mg) and sulfisoxazole (1mg) were substituted for amoxicillin and sulfamethoxazole, respectively. Ninety-nine *E. coli* isolates selected from a convenience sample of 41 individuals (1-7 isolates per stool sample) were compared. Isolates were adjusted to 0.5 McFarland turbidity (with 0.85% sterile NaCl), spread onto Mueller Hinton agar, and allowed to dry briefly before applying antibiotic discs. Zone sizes (mm) were recorded after 16-20 h incubation at 37˚C. An *E. coli* ATCC 25922 strain was used as a negative control and *E. coli* strain H4H ^11^ was used as a positive control. “Intermediate” zone sizes were classified as susceptible when comparing with breakpoint assay results. Pearson`s correlation coefficient was used to compare the proportion of resistant isolates for ten different antibiotics between two assays (Figure S1).

**Figure S1.** **Pearson’s correlation coefficient comparing the proportion of resistant isolates (n=99 total) as determined by breakpoint assay (red) and Kirby-Bauer (blue) for ten different antibiotics.** Antibiotics included amp (ampicillin), amx (amoxicillin), caz (ceftazidime), chl (chloramphenicol), cip, (ciprofloxacin) and kan (kanamycin), str, (streptomycin), sul (sulfamethoxazole), tet (tetracycline), tri (trimethoprim). MDR indicates resistance to three or more classes of antibiotics

**1.6 Variable Description**

Age was included as a binary variable (1=adult, 0=child) given the tendency for younger individuals to exhibit higher prevalence in both humans ^13–15^ and livestock ^16,17^. Whether the household was located in a rural or urban area was retained given evidence of a higher prevalence of antimicrobial resistance in urban areas ^18–20^ ^21^. We developed scaled variables for antibiotic use and for household hygiene. Composite scales were used to summarize indicators that could succinctly describe variables most likely associated to antimicrobial transmission and selection (see Table 1 in main manuscript for description of these items). Each hygiene item was given the same weighted and scales reflect the sum of the binary factors (1=yes, a practice was followed or 0 = no, a practice was not followed)

**1.7 Model specification**

Model specification proceeded by first partitioning resistance phenotypes by the variance within and between individuals. A random intercept model (“baseline” or “unconditional” model) was specified to justify a multilevel modeling approach through calculation of the intra-class correlation (ICC). The ICC ranged from 0.44 (Amx) to 0.72 (Cip). An ICC of 0.72, for example, indicates that about 72% of the variance in resistance is between individuals while around 28% is within individuals. Alternatively, the ICC can be interpreted as the extent to which isolates within the same individual are more alike compared with isolates from another individual^22^. For Cip, this means that resistance profiles of isolates within the same individual are 72% more similar relative to isolates within another individual and so should not be considered independent observations^22,23^. .

**1.8 Model Fit**

Model fit was assessed using McKelvey and Zavoina Pseudo R^2^, a goodness-of-fit measure that is based on variance decomposition of the estimated logits and has been recommended as measure for logistic multilevel models ^24–26^. Most values of McKelvey and Zavoina Pseudo R^2^ for the fixed and random effects were 0.3 or above, indicating good model fit. See Table S2 McKelvey and Zavoina Pseudo R^2^ values for estimations using both the fixed- and random-effects, the fixed-effects only, and the intraclass correlations.

**Table S1. Correlates of antibiotic resistance with interaction between antibiotic use and hygiene.** Across all antibiotics, the number of observations is 21,256 and the number of groups (households) is 274. Coefficients are provided with 95% confidence intervals. See Table 1 for variable definitions.

| Antibiotics | AB Use  Yes=1 | Household  hygiene scale | AB X Hygiene interaction | Participant  had diarrhea | Household  boiled milk | Adult/child  Adult=1 | Rural/urban  Urban=1 |
| --- | --- | --- | --- | --- | --- | --- | --- |
| Ampicillin | 0.25***  (0.12 - 0.53) | 0.58***  (0.51 - 0.66) | 1.36***  (1.19 - 1.55) | 1.33***  (1.13 - 1.56) | 0.83  (0.52 - 1.32) | 0.38***  (0.24 - 0.62) | 1.60*  (0.99 - 2.58) |
| Amoxicillin | 0.26***  (0.12 - 0.56) | 0.63***  (0.56 - 0.72) | 1.37***  (1.20 - 1.57) | 1.19**  (1.01 - 1.39) | 0.69  (0.44 - 1.08) | 0.43***  (0.27 - 0.67) | 1.22  (0.77 - 1.92) |
| Ceftazidime | 0.00***  (0.00 - 0.00) | 0.17***  (0.11 - 0.26) | 5.84***  (3.83 - 8.92) | 3.51***  (2.27 - 5.43) | 0.31**  (0.12 - 0.81) | 0.91  (0.36 - 2.29) | 4.02***  (1.42 - 11.36) |
| Chloramphenicol | 1.12  (0.34 - 3.69) | 0.75***  (0.61 - 0.92) | 0.97  (0.78 - 1.20) | 0.63***  (0.45 - 0.87) | 0.42***  (0.23 - 0.79) | 0.97  (0.52 - 1.80) | 1.68  (0.89 - 3.20) |
| Ciprofloxacin | 0.01***  (0.00 - 0.08) | 0.30***  (0.22 - 0.42) | 2.30***  (1.70 - 3.10) | 2.50***  (1.87 - 3.34) | 0.33***  (0.15 - 0.74) | 1.87  (0.83 - 4.23) | 3.18***  (1.35 - 7.49) |
| Kanamycin | 0.03***  (0.00 - 0.17) | 1.02  (0.80 - 1.31) | 1.42**  (1.05 - 1.91) | 4.81***  (3.26 - 7.11) | 0.84  (0.43 - 1.65) | 1.26  (0.63 - 2.50) | 1.37  (0.66 - 2.85) |
| Streptomycin | 0.16***  (0.07 - 0.36) | 0.60***  (0.53 - 0.69) | 1.53***  (1.32 - 1.76) | 0.84**  (0.70 - 0.99) | 0.73  (0.45 - 1.20) | 0.37***  (0.22 - 0.61) | 1.09  (0.66 - 1.80) |
| Sulfamethoxazole | 0.03***  (0.01 - 0.07) | 0.33***  (0.28 - 0.39) | 2.07***  (1.78 - 2.41) | 1.58***  (1.33 - 1.88) | 0.94  (0.53 - 1.66) | 0.41***  (0.23 - 0.73) | 1.42  (0.80 - 2.53) |
| Tetracycline | 0.18***  (0.08 - 0.38) | 0.59***  (0.52 - 0.68) | 1.46***  (1.28 - 1.68) | 1.18*  (1.00 - 1.40) | 0.62**  (0.39 - 0.98) | 0.47***  (0.29 - 0.75) | 0.82  (0.51 - 1.31) |
| Trimethroprim | 0.55  (0.25 - 1.20) | 0.63***  (0.55 - 0.72) | 1.15*  (1.00 - 1.32) | 1.28***  (1.08 - 1.50) | 0.93  (0.59 - 1.49) | 0.41***  (0.26 - 0.66) | 1.26  (0.78 - 2.03) |
| MDR | 0.05***  (0.02 - 0.12) | 0.41***  (0.35 - 0.48) | 1.84***  (1.59 - 2.13) | 1.39***  (1.17 - 1.64) | 0.67  (0.39 - 1.14) | 0.31***  (0.18 - 0.55) | 1.56  (0.90 - 2.69) |

*** P < 0.01, ** P < 0.05, * P < 0.1

b See Table 1 for definitions.

c Constant indicates the predicted mean OR when all variables are 0.

**Table S2. Fit Indices for models.** McKelvey and Zavoina Pseudo R^2^ and Intraclass Correlations.

| **Model** | **antibiotic** | **McKelvey&Zavoina-Pseudo-R2**  **Fixed Effects Only** | **McKelvey&Zavoina-Pseudo-R2**  **Fixed and Random effects** | **Intra-Class-Correlation (Level 2)** |
| --- | --- | --- | --- | --- |
| Model 1: No Interaction | Ampicillin | 0.1312 | 0.4306 | 0.4574 |
|  | Amoxicillin | 0.0906 | 0.4063 | 0.4414 |
|  | Ceftazifime | 0.2409 | 0.6263 | 0.6755 |
|  | Cholramphenicol | 0.2161 | 0.5657 | 0.606 |
|  | Ciprofloxacin | 0.3268 | 0.6862 | 0.7119 |
|  | Kanamycin | 0.1607 | 0.5457 | 0.5893 |
|  | Streptomycin | 0.0905 | 0.4653 | 0.4999 |
|  | Sulfamethoxazole | 0.2427 | 0.4899 | 0.4979 |
|  | Tetracycline | 0.1135 | 0.4368 | 0.4633 |
|  | Trimethoprim | 0.1417 | 0.4363 | 0.4602 |
|  | MDR | 0.1979 | 0.498 | 0.5175 |
| Model 2: Interaction between Antibiotic use and Hygiene | Ampicillin | 0.1564 | 0.4338 | 0.4574 |
|  | Amoxicillin | 0.1236 | 0.4105 | 0.4414 |
|  | Ceftazifime | 0.5269 | 0.7402 | 0.6755 |
|  | Cholramphenicol | 0.1065 | 0.54 | 0.606 |
|  | Ciprofloxacin | 0.4055 | 0.7053 | 0.7119 |
|  | Kanamycin | 0.1857 | 0.5602 | 0.5893 |
|  | Streptomycin | 0.1428 | 0.4712 | 0.4999 |
|  | Sulfamethoxazole | 0.3349 | 0.5034 | 0.4979 |
|  | Tetracycline | 0.1511 | 0.4402 | 0.4633 |
|  | Trimethoprim | 0.1492 | 0.4372 | 0.4602 |
|  | MDR | 0.2812 | 0.5083 | 0.5175 |

**Table S3. Correlation matrix: wealth scale, household hygiene scale and consumption of milk**

|  | Wealth Scale | Household Drinks Milk | Household hygiene scale |
| --- | --- | --- | --- |
| Wealth Scale | 1.00 |  |  |
| Household Drinks Milk | 0.133* | 1.00 |  |
| Household hygiene scale | 0.558* | 0.128* | 1.00 |

*p<0.05

References

1. Caudell, M. A. *et al.* Antimicrobial Use and Veterinary Care among Agro-Pastoralists in Northern Tanzania. *PLOS ONE* **12**, e0170328 (2017).

2. Omulo, S. *et al.* Evidence of superficial knowledge regarding antibiotics and their use: Results of two cross-sectional surveys in an urban informal settlement in Kenya. *PLOS ONE* **12**, e0185827 (2017).

3. Jarquin, C. *et al.* Salmonella on Raw Poultry in Retail Markets in Guatemala: Levels, Antibiotic Susceptibility, and Serovar Distribution. *Journal of Food Protection* **78**, 1642–1650 (2015).

4. Eisenhauer, I. F. *et al.* Estimating the Risk of Domestic Water Source Contamination Following Precipitation Events. *American Journal of Tropical Medicine and Hygiene* **94**, 1403–1406 (2016).

5. Eisenhauer, I. F., Celada, M. A. & Carlton, E. J. In search of safe and sufficient water: a portrait of household wells in rural Guatemala. *International Journal of Epidemiology* **45**, 677–682 (2016).

6. Enneman, A., Hernández, L., Campos, R., Vossenaar, M. & Solomons, N. W. Dietary characteristics of complementary foods offered to Guatemalan infants vary between urban and rural settings. *Nutrition Research* **29**, 470–479 (2009).

7. Guarnieri, M. J. *et al.* Effects of Woodsmoke Exposure on Airway Inflammation in Rural Guatemalan Women. *PLoS ONE* **9**, e88455 (2014).

8. Omulo, S. *et al.* The impact of fecal sample processing on prevalence estimates for antibiotic-resistant Escherichia coli. *Journal of Microbiological Methods* **136**, 71–77 (2017).

9. Caudell, M. A. *et al.* Identification of risk factors associated with carriage of resistant Escherichia coli in three culturally diverse ethnic groups in Tanzania: a biological and socioeconomic analysis. *The Lancet Planetary Health* **2**, e489–e497 (2018).

10. Lyimo, B., Buza, J., Subbiah, M., Smith, W. & Call, D. R. Comparison of antibiotic resistant Escherichia coli obtained from drinking water sources in northern Tanzania: a cross-sectional study. *BMC Microbiology* **16**, (2016).

11. Call, D. R. *et al.* blaCMY-2-Positive IncA/C Plasmids from Escherichia coli and Salmonella enterica Are a Distinct Component of a Larger Lineage of Plasmids. *Antimicrobial Agents and Chemotherapy* **54**, 590–596 (2010).

12. Subbiah, M., Top, E. M., Shah, D. H. & Call, D. R. Selection Pressure Required for Long-Term Persistence of blaCMY-2-Positive IncA/C Plasmids. *Applied and Environmental Microbiology* **77**, 4486–4493 (2011).

13. Dyar, O. J. *et al.* High prevalence of antibiotic resistance in commensal Escherichia coli among children in rural Vietnam. *BMC infectious diseases* **12**, 92 (2012).

14. Kalter, H. D. *et al.* Risk factors for antibiotic-resistant Escherichia coli carriage in young children in Peru: community-based cross-sectional prevalence study. *The American journal of tropical medicine and hygiene* **82**, 879–888 (2010).

15. Bartoloni, A. *et al.* Patterns of antimicrobial use and antimicrobial resistance among healthy children in Bolivia. *Tropical medicine & international health* **3**, 116–123 (1998).

16. Butaye, P., Devriese, L. A., Goossens, H., Ieven, M. & Haesebrouck, F. Enterococci with Acquired Vancomycin Resistance in Pigs and Chickens of Different Age Groups. *Antimicrobial Agents and Chemotherapy* **43**, 365–366 (1999).

17. Berge, A., Atwill, E. R. & Sischo, W. Assessing antibiotic resistance in fecal Escherichia coli in young calves using cluster analysis techniques. *Preventive veterinary medicine* **61**, 91–102 (2003).

18. Walson, J. L., Marshall, B., Pokhrel, B. M., Kafle, K. K. & Levy, S. B. Carriage of Antibiotic-Resistant Fecal Bacteria in Nepal Reflects Proximity to Kathmandu. *The Journal of Infectious Diseases* **184**, 1163–1169 (2001).

19. Eisenberg, J. N. S. *et al.* In-roads to the spread of antibiotic resistance: regional patterns of microbial transmission in northern coastal Ecuador. *Journal of the Royal Society Interface* **9**, 1029–1039 (2012).

20. Najjuka, C. F., Kateete, D. P., Kajumbula, H. M., Joloba, M. L. & Essack, S. Y. Antimicrobial susceptibility profiles of Escherichia coli and Klebsiella pneumoniae isolated from outpatients in urban and rural districts of Uganda. *BMC Research Notes* **9**, 235 (2016).

21. Mathai, E. *et al.* Antimicrobial resistance surveillance among commensal Escherichia coli in rural and urban areas in Southern India. *Tropical Medicine & International Health* **13**, 41–45 (2008).

22. Peugh, J. L. A practical guide to multilevel modeling. *Journal of School Psychology* **48**, 85–112 (2010).

23. Muthén, B. O. Multilevel Covariance Structure Analysis. *Sociological Methods & Research* **22**, 376–398 (1994).

24. Hox, J. J. *Multilevel analysis: Techniques and applications*. (Routledge, 2010).

25. Langer, W. *How to assess the fit of multilevel logit models with Stata?* https://EconPapers.repec.org/RePEc:boc:dsug17:05 (2017).

26. McKelvey, R. D. & Zavoina, W. A statistical model for the analysis of ordinal level dependent variables. *Journal of mathematical sociology* **4**, 103–120 (1975).
