# Supplementary material for: Antibiotic use and hygiene interact to influence the distribution of antimicrobial-resistant bacteria in low-income communities in Guatemala
Source: Sci Rep. 2020 Aug 13;10:13767. doi: 10.1038/s41598-020-70741-4 (PMC7426860; doi:10.1038/s41598-020-70741-4)
Supplement: Supplementary file 4 [file 41598_2020_70741_MOESM4_ESM.pdf]

COMMUNITY-BASED ASSESSMENT OF ANTIBIOTIC RESISTANT *ESCHERICHIA COLI* IN QUETZALTENANGO, GUATEMALA

**Read this information to participant and leave for the participants´reference

Thank you for being in our study! We would like to collect stool from you and one child less than 5 years old in the household. We want to test the stool for the presence of antibiotic-resistant bacteria and parasites. To complete this analysis we will take the stool to a lab in Guatemala City. Within two weeks we will provide you with the results of the parasite. If the test is positive we will provide you with the medication needed for treatment. By providing this stool sample, you will help us to learn more about antibiotic resistance in Quetzaltenango.

PLEASE READ ALL DIRECTIONS FIRST. PLEASE FOLLOW THEM CAREFULLY.

If you have not signed the consent form, please read and sign the form before proceeding. We will collect this form when we pick up the stool

1. This stool collection kit contains the following items:

- 2 plastic cups with screw top lids (called stool cups)
- 2 wooden sticks
- 2 pieces of crepe paper
- 1 lunch bag
- 1 pair of disposable gloves
- 1 small bottle of sanitizing gel for your hands
- Two bags for disposal of used collection materials

IMPORTANT: Please wash your hands before beginning.

DO NOT pass stool into the toilet or latrine.
DO NOT urinate on the stool or into the stool cup.
DO NOT allow anything to mix with the stool.

1. Wash your hands

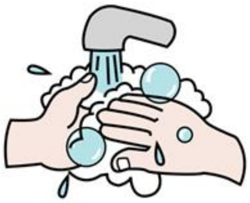

2. Pass stool into any clean and dry container, directly into the container, or on the crepe paper found in this kit.

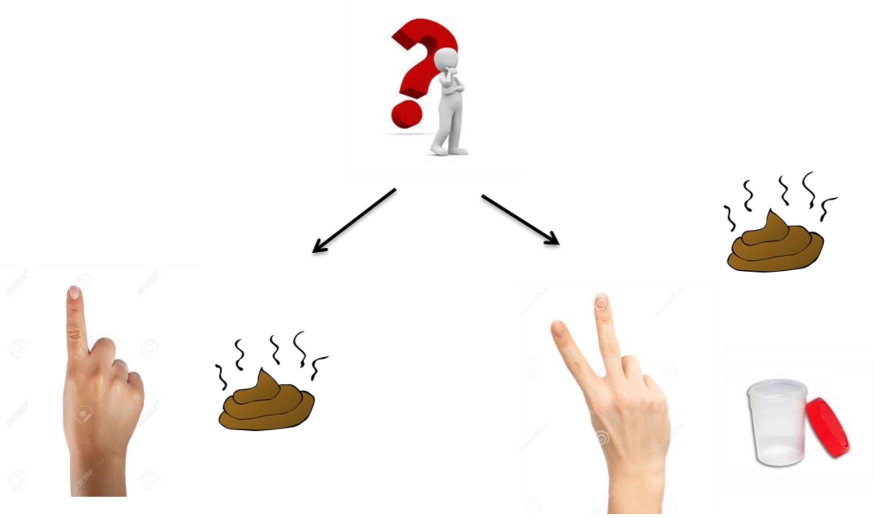

3. Put on the gloves

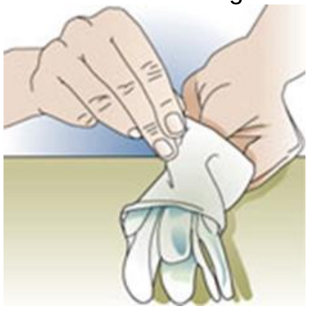

4. Open the stool cup. Use the stick to scoop stool into the labeled cup. Fill the cup up to about half way full if you can.

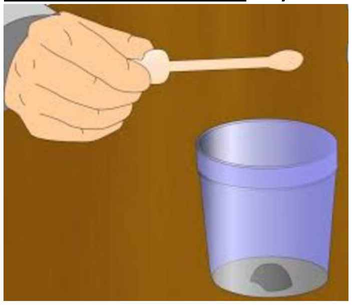

5. Screw the lid tightly on the cup.

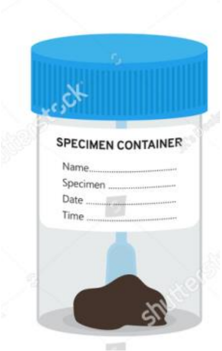

6. Put the cup into the plastic zip lock bag

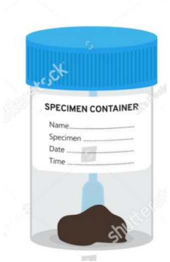

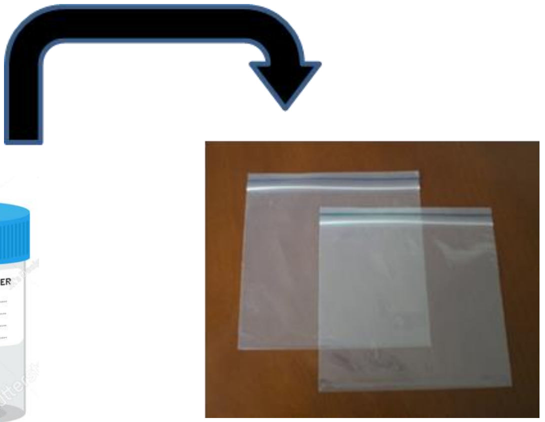

7. Put the cup/bag into the lunch bag

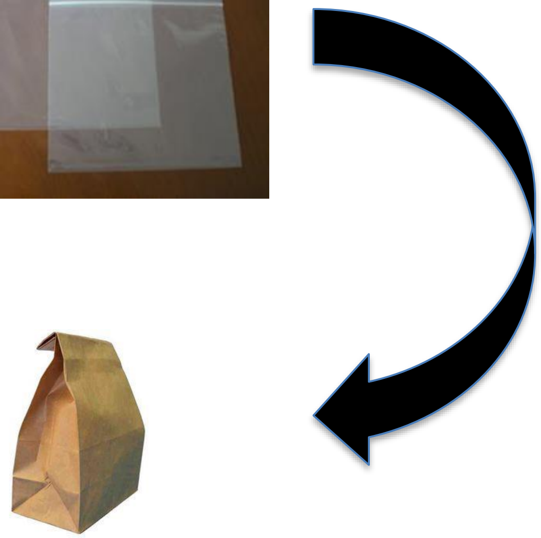

8. Discard excess stool where you normally defecate. Discard all other supplies in the garbage. If you prefer, or if it is easier for you, you can place used materials into a bag (provided) and the team that visits you from this study will dispose of these materials tomorrow.

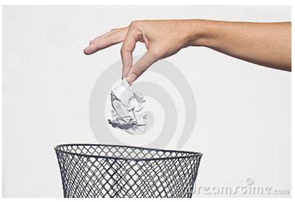

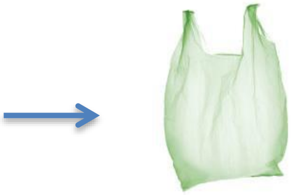

9. Apply sanitizing gel to your hands to ensure they are clean after collecting the sample.

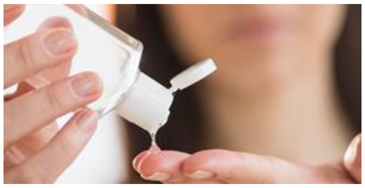

10. Store the filled stool cup in a cool dry place until we can pick it up.

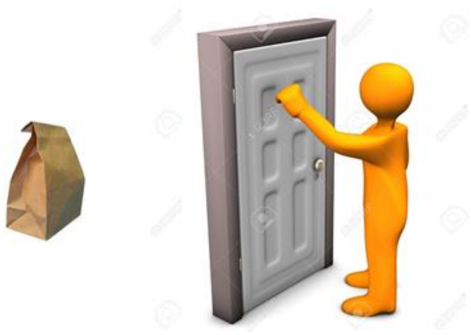

HELPFUL HINTS FOR CHILDREN IN DIAPERS

PLEASE TRY TO COLLECT THE STOOL AS YOU SEE YOUR CHILD HAVING A BOWEL MOVEMENT. WE DO NOT WANT THE STOOL TO SIT IN THE DIAPER FOR VERY LONG.

We will return tomorrow to pick up your stool. We would like the freshest stool you can give us. Please use one cup to collect the first stool you pass.

We will deliver a bag to you when we return to collect the medications you take during the months of November, December, January, and February, until we return.

THANK YOU
