# Supplementary material for: Antibiotic use and hygiene interact to influence the distribution of antimicrobial-resistant bacteria in low-income communities in Guatemala
Source: Sci Rep. 2020 Aug 13;10:13767. doi: 10.1038/s41598-020-70741-4 (PMC7426860; doi:10.1038/s41598-020-70741-4)
Supplement: Supplementary file 5 [file 41598_2020_70741_MOESM5_ESM.pdf]

Evaluación comunitaria de *Escherichia coli resistente a los* antibióticos en Quetzaltenango, Guatemala

**Leer la información en este informe al participante y dejar la hoja de información como recordatorio*:
¡Gracias por participar en nuestro estudio! Quisiéramos colectar una muestra de popó de usted y un niño menor de 5 años en la casa. Queremos examinar la muestra de popó para saber si tiene bacterias resistentes a antibióticos y para determinar si tiene parásitos. Vamos a llevar la muestra de popó a la capital para hacer las pruebas necesarias. Le notificaremos de los resultados de la prueba de parásitos dentro de 2 semanas de la entrega de la muestra. Si tiene parásitos, le daremos la medicina necesaria en el puesto de salud. Al darnos la muestra de popó, nos ayuda a aprender más sobre la resistencia antibiótica en el departamento de Quetzaltenango.

Por favor, lea todas las indicaciones antes de comenzar. SÍRVASE SEGUIRLAS CUIDADOSAMENTE.

.

Cada kit de recolección de popó contiene los siguientes elementos:

- 1 vaso de plástico con tapa de rosca (llamados vasos de popó)
- 1 palo de madera
- 1 pieza de papel craft
- 1 bolsa de papel opaca
- 1 par de guantes desechables
- 1 bolsa plástica
- 1 frasco de gel sanitario para limpiar las manos
- 1 bolsa de basura

IMPORTANTE: Por favor lávese las manos antes de comenzar.

NO recoja el popó de dentro del inodoro.
NO orine en el bote donde colocará el popó.
NO permita que nada se mezcle con el popó.

Instrucciones para el participante

1. Lávese las manos antes de colectar la muestra

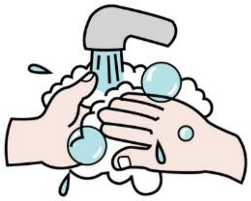

2. De preferencia, haga popó dentro del bote o si siente que no puede, hágalo sobre el papel craft.

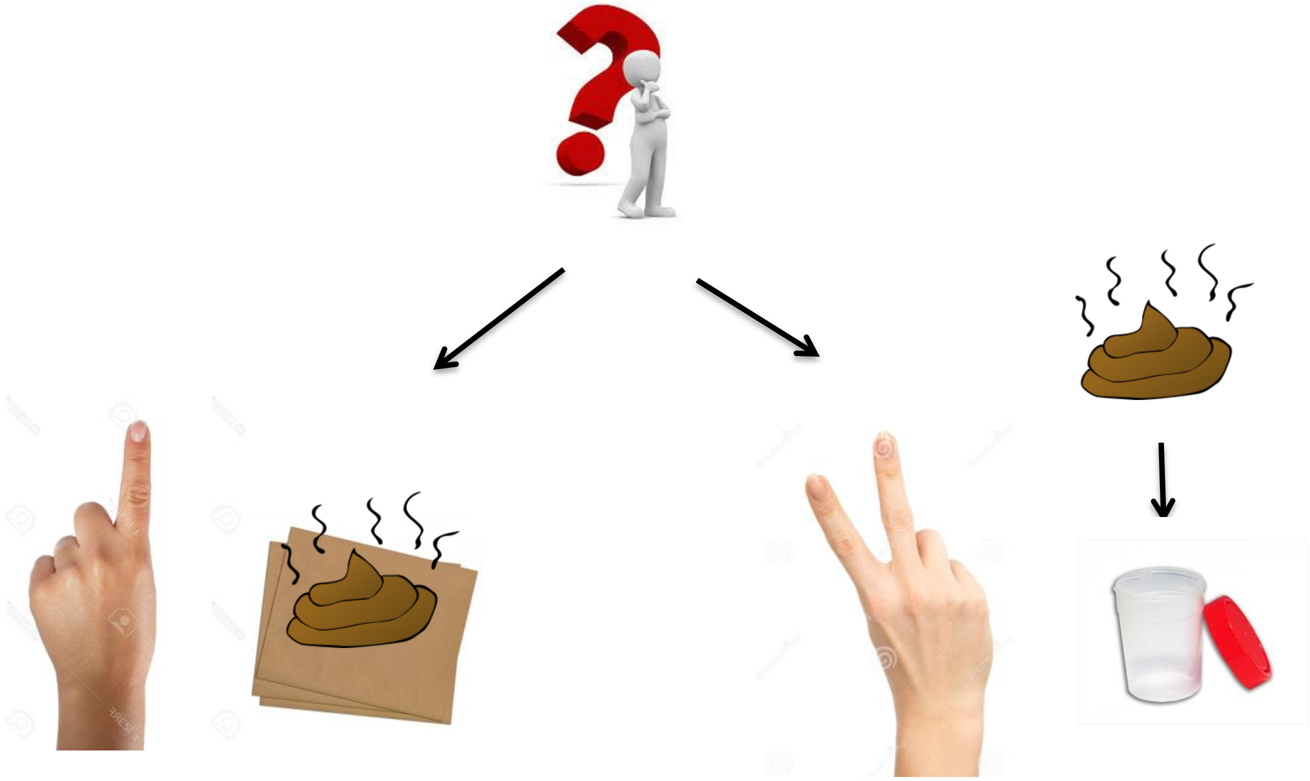

3. Póngase los guantes.

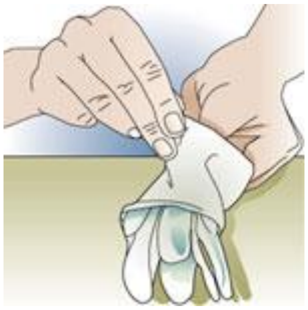

4. Abra el bote para el popó y recoja con el palito un poco de su popó y métalo en el bote hasta llenar hasta la mitad del bote.

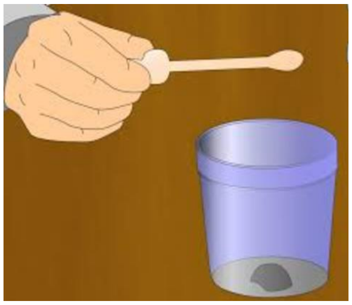

5. Cierre el bote muy bien con la tapadera.

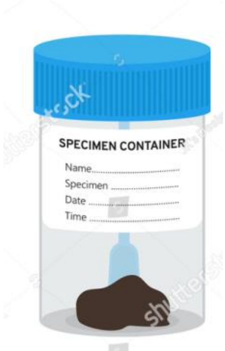

6. Meta el bote bien cerrado en la bolsa plástica.

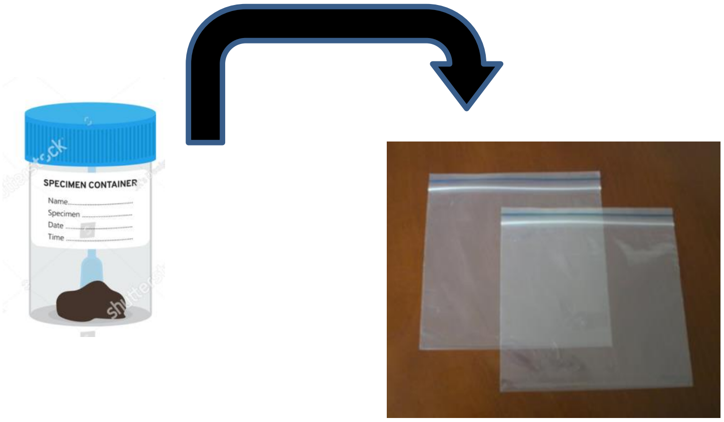

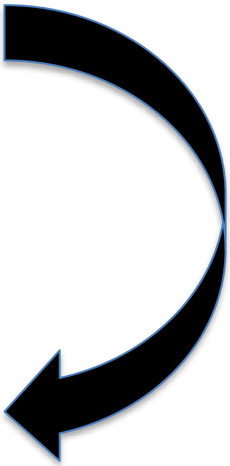

7. Meta la bolsa plástica con el bote, dentro de la bolsa de papel.

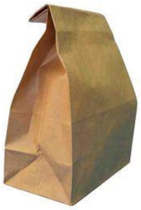

8. Tire el exceso de popó donde normalmente hace popó. Deseche todos los demás materiales en la basura. Si lo prefiere, o si es más fácil para usted, puede colocar los materiales sucios y ya usados en la bolsa que le entregamos a usted dentro del kit de recolección.

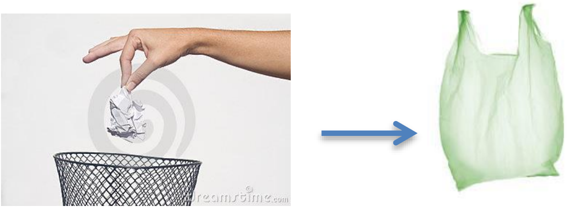

9. Lávese las manos y aplíquese el gel sanitario en las manos para asegurarse que estén limpias las manos.

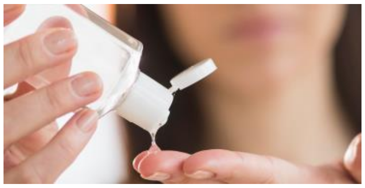

10. Guarde el vaso lleno de popó en un lugar seco y fresco hasta que un trabajador del estudio pase a recogerla (al final de la tarde o a la mañana siguiente).

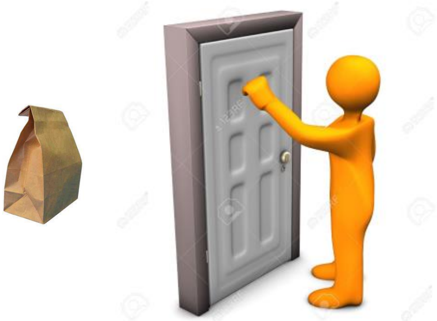

**CONSEJOS ÚTILES PARA NIÑOS EN PAÑALES**
POR FAVOR TRATE DE RECOGER EL POPÓ CUANDO USTED VEA QUE SU HIJO DEFECÓ. NO QUEREMOS QUE EL POPÓ PERMANEZCAN EN EL PAÑAL DURANTE MUCHO TIEMPO.

Regresaremos más tarde para recoger la muestra de popó. Nos gustaría una muestra que sea lo más fresca posible que pudiera darnos.
Le vamos a entregar una bolsa a usted cuando regresemos para colectar los medicamentos que toma durante los meses de Noviembre, Diciembre, Enero, Febrero, hasta que regresemos

¡GRACIAS!
